# Immediate and Sustained Outcomes and Moderators Associated With Metacognitive Training for Psychosis: A Systematic Review and Meta-analysis

**DOI:** 10.1001/jamapsychiatry.2022.0277

**Published:** 2022-03-23

**Authors:** Danielle Penney, Geneviève Sauvé, Daniel Mendelson, Élisabeth Thibaudeau, Steffen Moritz, Martin Lepage

**Affiliations:** 1 Douglas Mental Health University Institute, Montréal, Québec, Canada; 2 Department of Psychology, Université du Québec à Montréal, Montréal, Québec, Canada; 3 Department of Education and Pedagogy, Université du Québec à Montréal, Montréal, Québec, Canada; 4 Department of Psychology, McGill University, Montréal, Québec, Canada; 5 Department of Psychiatry, McGill University, Montréal, Québec, Canada; 6 Department of Psychiatry and Psychotherapy, University Medical Center Hamburg, Hamburg, Germany

## Abstract

**Importance:**

A substantial increase in the number of trials examining metacognitive training (MCT) for psychosis necessitates an updated examination of the outcomes associated with MCT.

**Objectives:**

To review the immediate and sustained associations of MCT with proximal (directly targeted) and distal (indirectly influenced) outcomes and assess treatment- and participant-related moderators to identify the potential factors associated with the expected heterogeneity of effect sizes.

**Data Sources:**

Eleven electronic databases were searched from 2007 to June 3, 2021 (alert until September 10, 2021). Reference lists of earlier meta-analyses and included reports were screened.

**Study Selection:**

Reports examined MCT and included participants with schizophrenia spectrum and related psychotic disorders (1045 reports identified; 281 assessed). There were no age, sex, gender, race and ethnicity, language, or study design restrictions. Two reviewers performed the selection of studies to be analyzed.

**Data Extraction and Synthesis:**

The Preferred Reporting Items for Systematic Reviews and Meta-analyses reporting guideline was followed. Data were extracted by 3 reviewers and pooled using random effects models. Hedges *g* effect sizes were computed. The Mixed-Methods Appraisal tool was used to assess study quality.

**Main Outcomes and Measures:**

Proximal outcomes were global positive symptoms, delusions, hallucinations, and cognitive biases. Distal outcomes were self-esteem, negative symptoms, quality of life, well-being, and functioning. Immediate and sustained outcomes were examined. Meta-regressions, subgroup, and sensitivity analyses assessed moderators.

**Results:**

This systematic review and meta-analysis included 43 studies (46 reports). Forty reports were synthesized in meta-analysis (N=1816 participants) and 6 reports were included in narrative review. In the studies examined, MCT was associated with positive symptoms (*g* = 0.50; 95% CI, 0.34-0.67), delusions (*g* = 0.69; 95% CI, 0.45-0.93), hallucinations (*g* = 0.26; 95% CI, 0.11-0.40), cognitive biases (*g* = 0.16; 95% CI, 0.03-0.29), self-esteem (*g* = 0.17; 95% CI, 0.03-0.31), negative symptoms (*g* = 0.23; 95% CI, 0.10-0.37), and functioning (*g* = 0.41; 95% CI, 0.12-0.69). These associations were maintained up to 1 year. The quality of life effect size was nonsignificant (*g* = 0.20; 95% CI, −0.07 to 0.47); only 1 study assessed well-being. Publication year was associated with moderated hallucinations (β = 0.04; 95% CI, 0.00-0.07). Overall, narrative review results corroborated meta-analytic findings.

**Conclusions and Relevance:**

In this meta-analysis, MCT for psychosis was associated with benefits up to 1 year postintervention in several treatment contexts. These findings suggest that MCT may merit integration in treatment guidelines for schizophrenia.

## Key Points

**Question:**

What are the immediate and sustained outcomes associated with metacognitive training (MCT) for psychosis, and are there specific treatment- or participant-related moderators of associations?

**Findings:**

This systematic review and meta-analysis of 43 studies (40 reports synthesized in meta-analysis, N=1816; 6 reports included in narrative review) on individuals with schizophrenia spectrum and related psychotic disorders found MCT was associated with reduced delusions, hallucinations, and cognitive biases. Metacognitive training was also associated with reduced negative symptoms and improved self-esteem and functioning.

**Meaning:**

The findings of this study suggest that MCT is an accessible evidence-based intervention, deliverable by a variety of mental health care professionals, and appears to be ready for large-scale implementation; MCT may merit inclusion in clinical guideline recommendations for the treatment of individuals with schizophrenia.

## Teaser

This systematic review and meta-analysis examines the use of metacognitive training for psychosis to help in treatment of individuals with psychosis.

## Introduction

Schizophrenia spectrum disorders are commonly considered the most severe psychiatric illnesses, profoundly affecting individuals, their families and caregivers, and society.^1,2^ Positive symptoms (hallucinations, delusions, and conceptual disorganization) represent the defining feature of schizophrenia spectrum disorders^3^ and figure predominantly in related psychotic disorders. Despite advancements in pharmacotherapy with antipsychotic medication, approximately 80% of people with schizophrenia spectrum disorders experience recurrent or persistent symptoms.^4,5^

Metacognitive interventions, such as metacognitive training for psychosis (MCT),^6,7^ metacognitive therapy,^8,9^ and metacognitive insight and reflection therapy,^10^ are psychological treatments aimed at improving metacognitive function, which may help to mitigate persistent symptoms and positive symptoms more generally. Metacognitive training for psychosis is the most widely investigated among these interventions and combines psychoeducation, cognitive bias modification, and strategy teaching.^7^

The intervention is low threshold: in lieu of directly targeting psychotic symptoms, MCT uses an indirect approach by promoting awareness of cognitive biases. Such biases are maladaptive thinking styles common to psychosis (eg, jumping to conclusions, belief inflexibility, and overconfidence in judgments) and are hypothesized to contribute to the formation and maintenance of positive symptoms, particularly delusions.^11,12^ Metacognitive training for psychosis thus aims to plant doubt in delusional beliefs through raising awareness of cognitive biases^7,13^ and aims to raise service engagement by proposing work on this less-confrontational objective first, which is likely to facilitate the therapeutic alliance and more direct work on psychotic symptoms.^13^

Metacognitive training has several important features as a brief (8-10 module) intervention. All therapeutic materials are available at no cost and are culturally sensitive (currently available in 37 languages). It is deliverable both as a group or individual intervention (MCT+),^11^ and given that modules are not successive, new group members may engage at any time. Furthermore, MCT is presented in a flexible manualized slide format with accompanying at-home activity sheets, which minimizes preparation and increases accessibility and adherence for less-experienced facilitators.^13^

To our knowledge, 8 meta-analyses have assessed MCT since its development in 2007.^14,15,16,17,18,19,20,21^ Previous studies report that MCT is acceptable at a large effect size (ES)^14^ and reduces delusions and other positive symptoms, with ES values ranging from small to moderate at postintervention^15,16,17,18^ and follow-up.^15^ Meta-analyses have also observed small to moderate reductions in cognitive biases^17^ and moderate improvements in insight.^17,19^ Two meta-analyses failed to observe significant ES values for MCT^20,21^; there is debate regarding whether conservative exclusion criteria and nonexhaustive search strategies may have contributed to these inconsistencies.^14,22,23,24^ One meta-analysis observed that neither an active control intervention nor the intervention delivery type statistically significantly moderated outcomes on delusions and other positive symptoms.^14^ Another reported that MCT+ (compared with group MCT) as well as studies published in Eastern compared with Western countries were statistically significant moderators.^15^ However, results were based on a small number of studies (n = 11) and were not maintained at follow-up.

Given the considerable influence that meta-analyses have on policy and international treatment guideline recommendations, it is necessary to rigorously address inconsistent findings, reassess specific intervention or participant-related moderators that may enhance outcomes, and update the literature as evidence accumulates. At least a dozen international studies focusing on psychotic symptoms have been published since the prior meta-analyses, for example, Chen et al,^25^ Acuña et al,^26^ and Tanoue et al.^27^ Together, these considerations provide the impetus for the present study.

Outcomes for this systematic review and meta-analysis are organized following a proximal-distal framework. Proximal outcomes include those directly targeted by MCT. Distal outcomes are those not directly targeted by MCT, but may be either directly or indirectly associated with improvement in proximal outcomes. Distal outcomes are identified as secondary clinical or person-centered variables often assessed in MCT trials, but not previously or thoroughly assessed in past meta-analyses. In this study, outcomes were examined quantitatively and qualitatively, from preintervention to postintervention and follow-up, which to our knowledge, is a novel contribution. Specific aims were to assess the immediate and sustained outcomes of MCT associated with improving proximal outcomes (global positive symptoms, delusions, hallucinations, and cognitive biases) and distal outcomes (self-esteem, negative symptoms, quality of life [QOL], well-being, social and global functioning) not thoroughly assessed in prior meta-analyses, and examine possible treatment- and participant-related moderators (risk of bias, type of analyses, study design, comparator type, diagnosis, intervention delivery format, manual adherence, number of sessions, facilitator training and credentials, year of report publication, age, sex, gender, medication, and duration of illness) to identify the potential causes of expected heterogeneity of ES values.

## Methods

The study protocol was registered on the PROSPERO database (CRD 42021259291) and the Preferred Reporting Items for Systematic Reviews and Meta-analyses (PRISMA) reporting guideline was followed.^28^ Detailed methods (eAppendix 1) are available in the Supplement.

**Figure 1.**
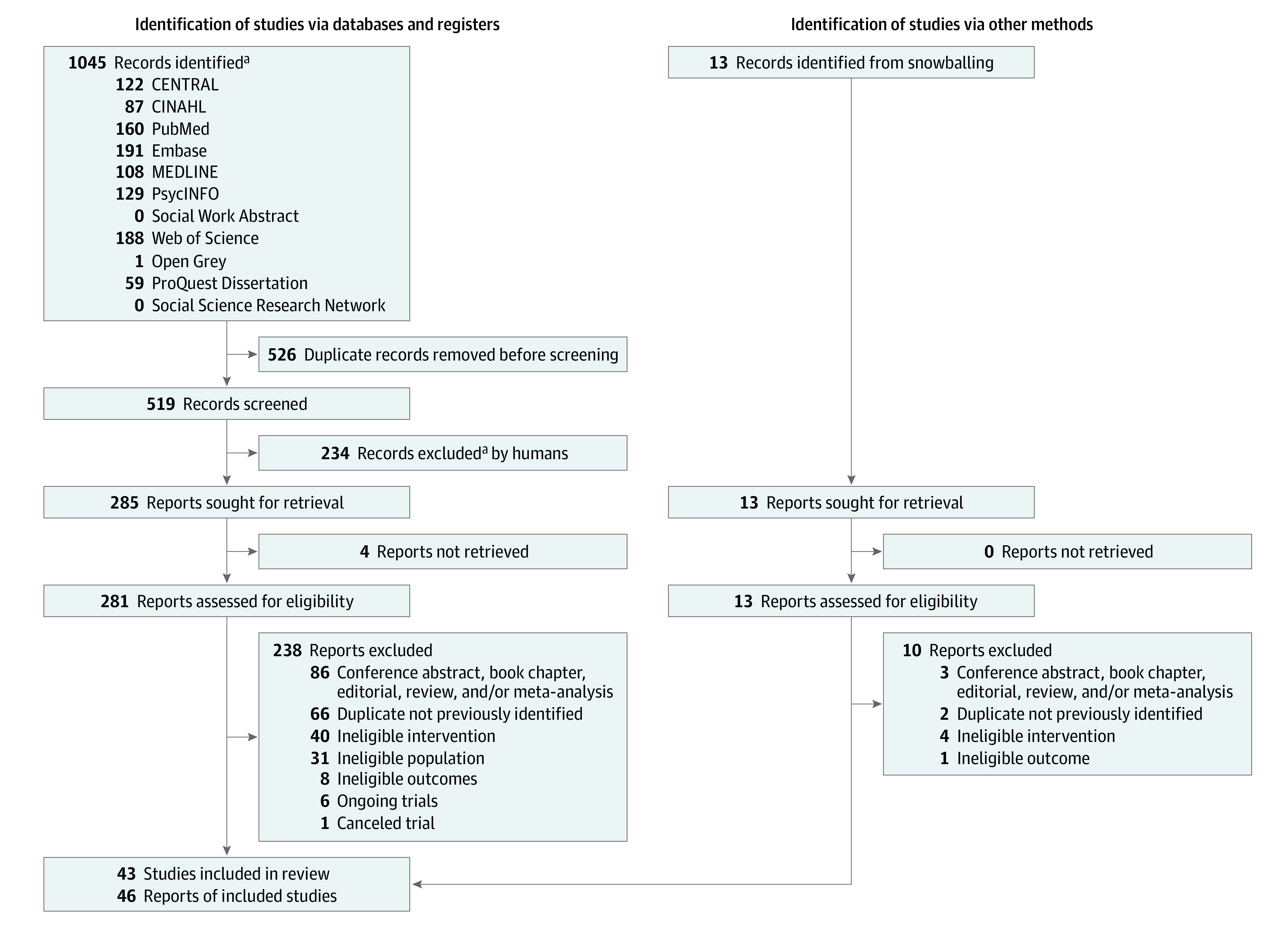
PRISMA 2020 Flow Diagram for New Systematic Reviews Which Included Searches of Databases, Registers, and Other Sources ^a^The title and/or abstract of a report indexed in a database or website.

The search was conducted on material published from 2007 to June 3, 2021, using 11 electronic databases: Cochrane Central Register of Controlled Trials, CINAHL (EBSCO), PubMed, Embase (Ovid), MEDLINE (Ovid), PsycINFO (Ovid), Social Work Abstracts (Ovid), and Web of Science. Grey literature was searched using OpenGrey, ProQuest Dissertations, and Social Science Research Network eLibrary. The search strategy is presented in eTable 1 in the Supplement. The PRISMA terms are *report* (a document providing information about a particular study, ie, a scientific paper), *record* (the title and/or abstract of a report indexed in a database or website), and *study* (a unique investigation or clinical trial). A systematic review and meta-analysis might have multiple reports, records, and studies (Figure 1). Searches were restricted to records published following the first MCT for psychosis publication (2007).^6^ The search did not include language restrictions or restrictions based on study design. The bibliographies of retrieved systematic reviews and meta-analyses and included reports were screened for additional reports. The codeveloper of MCT (S.M.) verified the comprehensiveness of the search results and, to mitigate conflict of interest, was not involved in study or report selection, data extraction, quality control, or analyses. Search updates were performed via automatic alert for the Web of Science database until September 10, 2021.

Figure 1 presents the report selection flowchart. Records were screened for eligibility by 2 of us (D.P. and D.M.), and discrepancies were resolved by another one of us (É.T.) until majority agreement was reached. Included reports were published in peer-reviewed journals; books and conference abstracts were excluded unless supplemental data were retrieved (for conference abstracts) from the author. Studies had to include participants with a diagnosis of schizophrenia spectrum or related psychotic disorder, and there were no sex, gender, race and ethnicity, or age restrictions. Studies also had to administer the original version or adaptations of MCT for psychosis (eTable 2 and eTable 6 in the Supplement). Acceptable adaptations included variability in the number of sessions, number of sessions per week, and session duration. Both individual and group formats were considered.

Data extraction was performed by 3 of us (D.P., D.M., and É.T.), and another of us (G.S.) reviewed 10% of the extracted data for accuracy. Discrepancies were resolved via majority agreement among the 4 reviewers. Data from the most recent report were selected when multiple reports corresponded to the same study.

### Proximal and Distal Outcome Measures

Only reports that investigated selected proximal (global positive symptoms, delusions, hallucinations, cognitive biases) and/or distal (self-esteem, negative symptoms, QOL, well-being, social and global functioning) outcomes were included. eTable 3 in the Supplement displays a comprehensive list of extracted variables. All measures and time points compatible with selected outcomes were sought.

### Methodologic Quality Assessment

Two of us (D.P. and D.M.) independently assessed study risk of bias using the Mixed Methods Appraisal Tool, version 2018.^29^ Methodological quality criteria and results are presented in eTable 4 in the Supplement. Interrater agreement on 10% of assessments was 85.71%. Disagreements were resolved between the 2 authors following examination and discussion of the Mixed Methods Appraisal Tool criteria.

### Data Synthesis Procedure

Selected outcomes were synthesized with separate meta-analyses using Comprehensive Meta-analysis, version 3.0 (Biostat). Reports were eligible for quantitative synthesis if they reported sample sizes, means (SDs), percentages, and/or ES values with a measure of variance (eg, 95% CIs), for pretreatment and posttreatment outcome measures. Meta-analyses were not limited to randomized clinical trials (RCTs); the rationale was guided by Shrier et al,^30^ Borenstein et al,^31^ and Efthimiou et al,^32^ who suggest that if studies address a common question (treatment effects on the same outcomes), limiting meta-analyses to RCTs is arbitrary; the process of randomization does not infer study quality (the extent that a study yields an unbiased estimate of effect). Meta-analyses based on non-RCTs typically yield ES estimates similar to those assessing RCTs.^30^ Therefore, we assessed study design as a moderator of MCT effectiveness and ran separate meta-analyses on proximal and distal outcomes using only RCTs to verify whether results were comparable. Information for studies and reports included in the systematic review but ineligible for the quantitative synthesis (n = 6) are displayed in Table 1^33,36,48,54,57,61^ and results are outlined in a narrative review. To conduct meta-analyses, Hedges *g* ES values were computed using the extracted data and were pooled for reports assessing multiple follow-up time points or for scales measuring the same outcome.

**Table 1.** Main Study Characteristics^a^

Source	Country	Design	Group type	Sample size, No.	Sex ratio, M:F	Age, mean (SD), y	Illness stage	DOI, mean (SD), y
Acuña et al,^26^ 2021^b^	Chile	RCT	MCT	25	14:11	27.52 (8.42)	NR	NR
			TAU	21	18:3	25.71 (4.72)		
Aghotor et al,^33^ 2010^c^	Germany	RCT	MCT	14	12:4^d^	28.9 (8.3)	NR	NR
			Active (newspaper discussion group)	12	8:6^d^	32.6 (12.1)		
Andreou et al,^11^ 2017	Germany	RCT	MCT+	46	21:25	36.91 (12.5)	NR	NR
			Active (CR+)	46	30:16	35.59 (13.1)		
Andreou et al,^11^ 2017	Germany	Cohort^e^	MCT	22	16:7^d^	36.85 (12.6)	NR	NR
Balzan et al,^34^ 2014	Australia	Non-RCT	MCT+	14	11:3	38.00 (8.11)	MEP	15.89 (8.51)
			TAU	14	9:5	35.21 (8.27)		9.71 (4.60)
Balzan et al,^35^ 2019^b^	Australia	RCT	MCT+	27	15:12	35.37 (9.84)	MEP	9.85 (8.47)
			Active (CR)	27	17:10	39.04 (7.48)		12.37 (7.95)
Briki et al,^36^ 2014^c^	France	Case series	MCT	7	3:4	29 (NR)	NR	NR^f^
Briki et al,^37^ 2014	France	RCT	MCT	25	16:9	41.1 (8.1)	NR	14.6 (8.4)
			Active (supportive therapy)	25	17:8	41.1 (12.4)		17.8 (10.9)
Chen et al,^25^ 2021	China	RCT	MCT	58	24:34	55.28 (9.51)	MEP	22.69 (12.02)
			Other (community-based rehabilitation)	62	24:38	52.90 (12.14)		23.35 (12.70)
de Pinho et al,^38^ 2021	Portugal	RCT	MCT	26	14:13^d^	48.30 (9.89)	NR	NR
			TAU	26	16:13^d^	52.66 (7.14)		
Erawati et al,^39^ 2014^b^	Indonesia	Non-RCT	MCT+	26	16:10	37.07 (10.75)	NR	NR
			TAU	26	15:11	42.00 (12.46)		
Favrod et al,^40^ 2011	Switzerland	Cross-sectional analytic study	MCT	18	11:7	41.8 (10.1)	NR	NR
Favrod et al,^41^ 2014	Switzerland	RCT	MCT	24	17:9^d^	36.85 (10.38)	NR	NR
			TAU	24	17:9^d^	36.58 (9.76)		
Z. Fekete, MA, personal communication, September 2021^b^	Hungary	RCT	MCT	23	11:12	44.22 (10.45)	MEP	16.16 (7.76)
			TAU	23	11:12	38.39 (10.41)	MEP	11.32 (8.74)
Ferwerda et al,^42^ 2010	Netherlands	Cohort	MCT	29	22:7	37.3 (9.1)	MEP	NR
Fujii et al,^43^ 2021	Japan	RCT, crossover	MCT	9	6:3	54.00 (7.6)	MEP	31.78 (6.16)
			TAU	8	4:4	54.50 (8.63)		33.38 (10.43)
Gawęda et al,^44^ 2015	Poland	RCT	MCT	23	11:12	50.41 (10.71)	MEP	22.96 (10.05)
			TAU	21	11:10	51.65 (10.25)		20.61 (11.30)
Ishikawa et al,^45^ 2020^b^	Japan	RCT	MCT	24	13:11	46.04 (8.37)	NR	19.58 (8.95)
			TAU	26	12:14	48.96 (8.54)		22.5 (8.84)
Kowalski et al,^46^ 2017	Poland	RCT	MCT, JTC	12	9:3	28 (5.41)	NR	6.42 (6.84)
			MCT, ToM	9	8:1	29.11 (4.43)		4.44 (1.81)
			Active (current events discussion)	10	5:5	31.7 (4.81)		8.30 (6.95)
Kumar et al,^47^ 2010^c^	India	RCT	MCT	8	8:0	31.50 (7.98)	NR	7.63 (7.74)
			TAU	8	8:0	34.13 (8.20)		6.50 (5.21)
Kumar et al,^48^ 2015	India	Case report	MCT+	1	0:1	36 (NA)	MEP	NR
Kuokkanen et al,^49^ 2014^b^	Finland	RCT	MCT	10	10:0	42.0 (10.4)	MEP	16.4 (10.3)
Kuokkanen et al,^50^ 2015			TAU	10	10:0	45.1 (14.3)		16.5 (9.2)
J.M. Lopez, PhD, personal communication, July 2021^b^	Spain	RCT	MCT	18	21:18^d^	45.6 (9.9)	Mixed (both first and multiple episodes)	NR
			Active (PE)	16	20:18^d^	49.8 (9.3)		
Moritz et al,^51^ 2011^a^	Germany	RCT, crossover	MCT	18	15:3	33.6 (8.8)	MEP	NR
			TAU	18	13:5	31.9 (7.0)		
Moritz et al,^13^ 2011	Germany	RCT	MCT	24	17:7	32.63 (12.48)	NR	2.96 (2.87)^g^
			Active (CR+)	24	14:10	35.46 (9.10)		3.59 (3.06)^g^
Moritz et al,^52^ 2013	Germany	RCT	MCT	76	45:31	36.82 (11.12)	Mixed (both first and multiple episodes)	NR
Moritz et al,^53^ 2014			Active (CR+)	74	49:25	32.68 (9.54)		
Moritz et al,^54^ 2018^c^								
Naughton et al,^55^ 2012	Ireland	Cohort	MCT	11	11:0	37.5 (10.6)	NR	NR
			Waitlist	8	8:0	35.62 (11.2)		
Ochoa et al,^56^ 2017^b^	Spain	RCT	MCT	65	44:21^d^	27.05 (7.94)	FEP	2.15 (2.01)
Salas-Sender et al,^57^ 2020^c^			Active (PE)	57	41:16^d^	28.21 (6.73)		2.46 (2.07)
Ochoa et al,^58^ 2020^b^	Spain	RCT	MCT+	24	26:10^d^	27.58 (6.72)	FEP	2.09 (NR)
			TAU	21	18:15^d^	29.50 (7.74)		2.66 (NR)
Park et al,^59^ 2020	South Korea	RCT	MCT	30	18:12	38.37 (9.05)	NR	13.70 (8.50)
			Active (educational material on social skills)	29	19:10	40.86 (7.34)		14.90 (8.67)
Pos et al,^60^ 2018	Netherlands	RCT	MCT	20	18:7^d^	23.59 (3.03)	FEP	NR
			Active (OT)	18	22:3^d^	23.08 (4.16)		
D. Raucher-Chéné, MD, personal communication, August 2021^b^	Canada	Cohort	MCT (virtual)	14	7:7	30.7 (9.4)	MEP	7.1 (7.3)
Schneider et al,^61^ 2018^c^	Germany	Cohort	MCT	176	94:82	35.2 (12.4)	NR	NR
Shan et al,^62^ 2021	China	RCT	MCT	19	12:7	26.05 (5.81)	NR	NR
			Other (recreational activities)	20	15:5	22.75 (4.38)		
Simón-Expósito et al,^63^ 2019	Spain	Non-RCT	MCT	11	NR	42.82 (7.5)	MEP	21.55 (8.26)
			TAU	11		47.27 (12.63)		24.36 (11.48)
So et al,^64^ 2015	Hong Kong	RCT, crossover	MCT+	23	12:11	32.35 (12.87)	NR	NR
			Waitlist	21	12:9	35.62 (10.89)		
Ho-Wai So et al,^65^ 2021	Hong Kong	RCT	MCT	27	12:15	42.78 (14.54)	NR	NR
			TAU	29	18:11	40.21 (13.27)		
Tanoue et al,^27^ 2021^b^	Japan	Cross-sectional analytic study	MCT	22	10:12	49.4 (10.4)	MEP	22.5 (9.5)
Ussorio et al,^66^ 2016	Italy	Cross-sectional analytic study^h^	MCT	56	41:15	22.3 (4.6)	FEP	1.31 (5.35)
van Oosterhout et al,^67^ 2014	Netherlands	RCT	MCT	75	54:21	38.3 (11.1)	NR	NR
			TAU	79	56:23	36.8 (8.7)		
Yildiz et al,^68^ 2018	Turkey	RCT	MCT	10	6:4	33.1 (10.7)	NR	13.6 (6.1)
			Active (PSST)	10	7:3	37.4 (4.6)		13.2 (8.4)
Zalzala et al,^69^ 2019^b^	United States	RCT	MCT	16	9:7^d^	31.50 (6.06)	NR	10.85 (5.71)
			Active (healthy living group)	16	9:8^d^	32.27 (6.28)		9.13 (7.80)

Abbreviations: CR, cognitive remediation; CR+, individual cognitive remediation; DOI, duration of illness; FEP, first episode of psychosis; MCT, metacognitive training; MEP, multiple episodes of psychosis; NA, not available; NR, not reported; OT, occupational therapy; PE, group psychoeducation; PSST, psychosocial skills training; RCT, randomized clinical trial; TAU, treatment as usual.

^a^ Total studies, 43; total reports, 46. eAppendix 2 in the Supplement provides the complete reference list of included reports. Studies reporting on overlapping trials are grouped; reports are grouped and represent 1 study. Study design was based on Mixed Method Appraisal Tool guidelines.

^b^ Data provided by study author.

^c^ Included only in narrative review.

^d^ Sex ratios at baseline, with attrition unaccounted for.

^e^ Two patient groups: medication responders and nonresponders.

^f^ Data reported in histogram format and were not extractable.

^g^ Years since first admission.

^h^ Two patient groups: long and short duration of untreated illness.

### Moderator Analyses

Subgroups and *Q* statistics with significance tests were used for the following categorical variables: risk of bias, type of analyses, study design, comparator type, intervention delivery format, manual adherence, number of sessions, facilitator training, and facilitator credentials. Meta-regression analyses were performed for continuous variables (diagnosis [% schizophrenia spectrum disorders], year of publication, age, sex [% male], medication, and duration of illness).

### Estimation of Evidence

Sensitivity analyses estimated the correlations between pretreatment and posttreatment scores when they were not reported.^70^ A conservative value of 0.7 was used when overall results were robust to the use of imputed correlations, as recommended by Rosenthal.^71^ Risk of publication bias was assessed via visual examination of the funnel plot by one of us (G.S.), the Egger asymmetry test,^72^ and the Rosenthal^73^ fail-safe N for all outcomes. Cochran *Q* statistic^74^ and the *I*^2^ index^75^ were calculated to estimate heterogeneity of ES values. A random-effects model was used given the anticipated differences between studies regarding test administration and MCT intervention features (eg, individual vs group format).^76^

## Results

Based on our criteria, 43 studies (46 reports) were included in the present review (eAppendix 2 in the Supplement); 30 were RCTs (70%), 11 were non-RCTs (25%), and 2 were quantitative descriptive studies (5%). Forty reports (N = 1816 participants) were synthesized with meta-analysis. Table 1 presents the main characteristics of included studies and reports (Z. Fekete, MA, personal communication, September 2021; J.M. Lopez, PhD, personal communication, July 2021; and D. Raucher-Chéné, MD, personal communication, August 2021)^11,13,25,26,27,33,34,35,36,37,38,39,40,41,42,43,44,45,46,47,48,49,50,51,52,53,54,55,56,57,58,59,60,61,62,63,64,65,66,67,68,69^ and Table 2 displays participant characteristics. Table 3^33,36,48,54,57,61^ and eAppendix 1 in the Supplement present the narrative review results of the 6 nonincluded studies and reports (eg, did not report ES values, secondary analyses). eTable 5 and eTable 6 in the Supplement present additional study and report characteristics, forest plots including all studies and reports by outcome (eFigure 1 in the Supplement), and a list of excluded and ongoing trials (eTable 7 in the Supplement).

**Table 2.** Participant Characteristics of Included Studies^a^

Characteristic	No. of studies reporting	Mean (SD) [range]
Age, y	43	36.89 (7.81) [22.30-55.28]
Duration of illness, y	22	13.05 (8.34) [1.31-32.53]
Chlorpromazine dose equivalent, mg	19	563.40 (324.77) [114.40-1519.40]
Male participants, %	41	63.19 (14.65) [41-100]
Schizophrenia spectrum disorder, %	41	94.24 (12.23) [59-100]
Other psychotic diagnosis, %	41	5.73 (12.22) [0-41]

^a^ Total studies, 43; total reports, 46. eTable 5 in the Supplement lists diagnoses of all included participants in each study.

**Table 3.** Narrative Review Results^a^

Source	Study goal	Outcomes of interest	Results
Aghotor et al,^33^ 2010	Assess MCT feasibility and preliminary efficacy	Positive symptoms; cognitive bias	Nonsignificant effect sizes for positive symptoms (*d* = 0.43) and cognitive bias (*d* = 0.31)
Briki et al,^36^ 2014	Effect of MCT on functioning	General and social functioning	Improvements in general and social functioning, reported graphically
Kumar et al,^48^ 2015	Effect of 12 sessions of MCT+	Positive and negative symptoms; general psychopathologic factors; belief conviction; social functioning	Improvements in positive and negative symptoms, general psychopathologic factors, interpersonal relationships, and social functioning; reductions in belief conviction
Moritz et al,^54^ 2018	Identify moderators of symptomatic outcome	Cognitive biases; cognitive insight; general psychopathologic factors; positive symptoms; QOL; self-esteem	Patients presenting low self-esteem, poor QOL, and social anxiety/withdrawal (per PANSS items N4 and G16) might benefit the most from MCT
Salas-Sender et al,^57^ 2020	Assess gender differences in response to MCT in FEP	Positive and negative symptoms; cognitive bias; functioning	Women showed larger improvements in personalizing bias and irrational beliefs related to dependence; men improved more on intolerance to frustration and JTC; no differences on positive or negative symptoms
Schneider et al,^61^ 2018	Effect of MCT following individual modules	Positive symptoms; cognitive bias	Improvement in positive symptoms (small ES) after MCT theory of mind module II; greatest cognitive bias reduction (small to medium ES) following module 3 (changing beliefs); increases in positive symptoms and cognitive bias severity following self-esteem (module 9) and mood (module 8) modules

Abbreviations: ES, effect size; FEP, first-episode psychosis; JTC, jumping to conclusions; MCT, metacognitive training; MCT+, individual MCT; PANSS, Positive and Negative Syndrome Scale; QOL, quality of life.

^a^ eAppendix 2 in the Supplement provides the complete reference list of included reports.

### Outcomes of MCT

**Figure 2.**
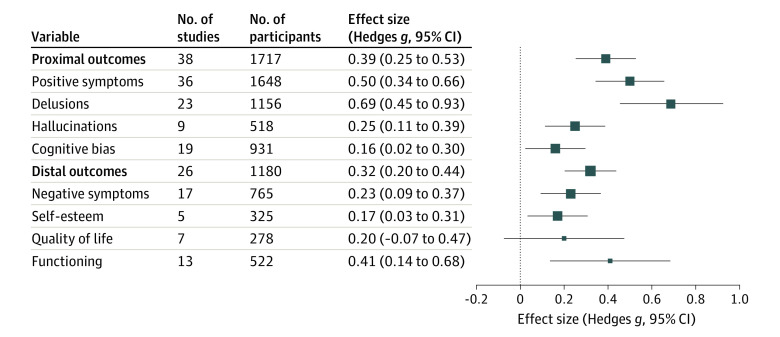
Effect Sizes of Metacognitive Training for Proximal and Distal Outcomes Square sizes represent the weight of the SE of the effect size. Higher precision studies (ie, a smaller SE) contribute to larger weights, and thus larger squares, than lower precision studies.

As shown in Figure 2, a small to moderate ES was observed for global proximal outcomes (ie, directly targeted by MCT: *g* = 0.39; 95% CI, 0.25-0.53; *P* < .001; 38 reports). When proximal outcomes were analyzed separately, global evaluations of positive symptoms reached a moderate ES (*g* = 0.50; 95% CI, 0.34-0.67; *P* < .001; 36 reports), the largest ES was obtained for delusions (*g* = 0.69; 95% CI, 0.45-0.93; *P* < .001; 23 reports), and small ES values were observed for hallucinations (*g* = 0.26; 95% CI, 0.11-0.40; *P* < .001; 9 reports) and cognitive biases (*g* = 0.16; 95% CI, 0.03-0.29; *P* < .001; 19 reports).

A small to moderate ES was also observed for distal outcomes (*g* = 0.31; 95% CI, 0.19-0.44; *P* < .001; 26 reports). Separate analyses revealed small but significant ES values for self-esteem (*g* = 0.17; 95% CI, 0.03-0.31; *P* = .01; 5 reports) and negative symptoms (*g* = 0.23; 95% CI, 0.10-0.37; *P* < .001; 17 reports); a small to moderate ES for functioning (*g* = 0.41; 95% CI, 0.12-0.69; *P* < .001; 13 reports); and a small, nonsignificant ES for QOL (*g* = 0.20; 95% CI, −0.07 to 0.47; *P* = .14; 7 reports). No changes in the direction of effect emerged for meta-analyses assessing MCT effectiveness (including only RCTs); however, analyses were underpowered for QOL and self-esteem and showed a trend for cognitive biases and functioning. Results on MCT effectiveness are located in eFigure 4 in the Supplement; eTable 14 in the Supplement presents ES comparisons between all study designs and RCTs only (ie, effectiveness) analyses.

### MCT Maintenance Effectiveness

Maintenance effectiveness (eTable 8 in the Supplement) was analyzed for RCTs by comparing the experimental and control groups on their difference scores between follow-up and posttreatment. Both groups maintained the therapeutic level reached at posttreatment until 1 year follow-up for all outcomes, evidenced by small, nonsignificant ES values for change over time (*g* values from 0.01 to 0.16; *P* values from .15 to .95). Thus, therapeutic gains made by the experimental group were steadily maintained. In additional analyses comparing the difference scores between follow-up and baseline for both groups, small to moderate ES values were obtained for proximal (*g* = 0.39; 95% CI, 0.16-0.61; *P* = .001; 14 reports) and distal (*g* = 0.30; 95% CI, 0.14-0.46; *P* = .001; 11 reports) outcomes. These results further indicate that net therapeutic gains remain significant even 1 year following MCT. Results pertaining to the maintenance of therapeutic effectiveness greater than 1 year are preliminary owing to an insufficient number of studies (eTable 8 in the Supplement).

### Moderator Analyses

Results of the moderator analyses are displayed in eTable 9, eTable 10, and eFigure 2 in the Supplement. The only significant moderator was year of publication, observed for hallucinations (β = 0.04; 95% CI, 0.00-0.07; *P* = .03). Larger ES values were reported in more recently published reports. Although some other moderators reached statistical significance, results are not interpretable owing to data not reported in subgroups or an insufficient number of reports per subgroup.

### Estimation of Evidence

Lower quality studies had significantly lower ES values for distal (between-group comparison, *Q*_4_ = 9.33; *P* = .05) but not proximal outcomes. Significant *Q* statistics for heterogeneity were obtained for most outcomes in the posttreatment − baseline analyses (eTable 11 in the Supplement). Similarly, *I*^2^ values suggest the presence of moderate to strong heterogeneity for proximal and distal outcomes in general, and global positive symptoms, delusions, QOL, and functioning. Significant findings on Egger tests for hallucinations, cognitive biases, self-esteem, negative symptoms, and QOL suggest the presence of publication bias (eTable 12 and eFigure 3 in the Supplement). Sensitivity analyses using different correlation values to estimate the level of association between scores of different time points reached comparable results (eTable 13 in the Supplement).

## Discussion

This comprehensive and methodologically rigorous meta-analysis facilitates a more precise estimate of the associations with and effectiveness of MCT with multiple outcomes and suggests MCT is a viable treatment for psychosis. The observed findings associated with positive symptoms exceed those reported in earlier meta-analyses.^14,15,16,17,18^ Larger ES values appeared to be predominantly associated with the inclusion of newer high-quality trials (eFigure 1 in the Supplement), but direction of the outcomes did not differ significantly when meta-analyses were restricted to RCTs. The magnitude of observed association with lowered delusions and hallucinations provides evidence to support the larger-scale implementation of MCT in the treatment of positive symptoms. Given the persistent and debilitating nature of positive symptoms, providing evidence that may help establish the viability of a low-threshold and accessible intervention that attenuates these symptoms is a key implication of this work.

Metacognitive training was also associated with improved distal outcomes, which we believe is a novel contribution. We observed small yet significant ES values for negative symptoms and self-esteem. The presence of low self-esteem and the persistent nature of negative symptoms are well established^77,78^ and are often directly targeted in psychological intervention. Improvements in these outcomes may be a contributing factor in the significant amelioration we observed in functioning, as in other studies,^79,80^ at postintervention. Metacognitive training also demonstrated effectiveness on negative symptoms. Results thus suggest the effectiveness of MCT with regard to global positive symptoms, delusions, hallucinations, and negative symptoms.

This meta-analysis also supports the sustained effectiveness of MCT, up to 1-year following the intervention, on all significant outcomes. The maintenance of treatment gains is critical in the long-term functioning of individuals with severe mental illness, and markedly so in psychotic disorders given the experience of persistent, debilitating symptoms. Most of these individuals are followed up in the public health system and often have limited personal resources to access private sector services.^81^ A durable, short-term, intervention (with group option) may therefore help to alleviate burden (ie, cost, specialized resources, waitlists) associated with the ongoing need for access to psychological services targeting persistent symptoms. Supporting the treatment gains of MCT thus has a pragmatic implication for care management.

The positive outcomes of MCT were observed regardless of age, sex, illness duration, and medication dosage. It can be successfully delivered by a variety of mental health practitioners, either as an 8-, 10-, or 16-session group or individual intervention. Such attributes, coupled with the establishment of treatment gains, align with broader implementation given the reality of cost-benefit mandates in the public mental health system, and yet remain compatible with person-centered models of care. Year of publication moderated the association between MCT and hallucinations, such that newer studies reported higher ES values. However, this finding should be interpreted with caution given the small number of included reports (n = 9). No other participant or treatment characteristic emerged as a moderator between MCT and any other proximal or distal outcome. Prior evidence suggests that women may improve more in general symptoms compared with men after MCT^57^; however, no sex-specific benefits were observed following meta-analysis.

It is important to position these findings within the broader context of evidence-based psychological interventions for psychosis. Cognitive behavioral therapy for psychosis and cognitive remediation are 2 well-established interventions. Meta-analyses examining cognitive behavioral therapy for psychosis have observed a small to moderate ES for delusions and small ES values for hallucinations, negative symptoms,^82^ and functioning.^83^ Similarly, prior cognitive remediation meta-analyses have reported small to moderate ES values for negative symptoms, global symptoms, and functioning.^84,85,86^ Thus, ES values observed for MCT appear similar to these other evidence-based interventions. The open-access availability of the intervention, combined with visual presentations and clinical e-training, makes MCT an accessible option for any mental health practitioner aspiring to deliver an evidence-based psychological intervention for psychosis.

Although ES values were significant for cognitive biases, noted benefits were lower than those observed in the Sauvé et al^17^ meta-analysis. Sauvé et al reported on a combination of 5 metacognitive interventions and their variants, which likely speaks to this discrepancy. Another potential explanation concerns the construct validity of popular standard measures of cognitive bias, such as the beads/fish tasks.^17,87^ Yet, in line with previous hypotheses,^11,12^ our findings suggest that MCT likely attenuates the overall outcomes associated with maladaptive thinking styles in the maintenance of positive symptoms, particularly given that symptoms are not directly addressed in the intervention, and evidenced by the significant reductions we observed in delusions and hallucinations. Hence, even a small reduction in cognitive biases is clinically meaningful.

The nonsignificant ES for QOL was unexpected given observed improvements in functioning, considerable reductions in psychotic symptom severity, and the negative association between QOL and psychotic symptoms.^88^ However, the QOL meta-analysis was likely underpowered with the inclusion of only 7 reports. A 3-year follow-up RCT assessing MCT efficacy revealed improvements in QOL and self-esteem compared with active control.^53^ These results were nonsignificant at 4-week and 6-month postintervention evaluation, perhaps speaking to a delayed effect of MCT on these outcomes. The variability of QOL domains assessed by the included measures (eg, impact of symptoms, well-being and satisfaction, general health status) may be another important factor accounting for null findings. Furthermore, our search did not yield sufficient studies to examine well-being (n = 1), although well-being is deemed a distinct construct.^89^ Given the importance of these outcomes in person-centered/patient-oriented recovery models,^90^ future trials would benefit from more precise examination and better operationalization of these constructs.

### Strengths and Limitations

This meta-analysis has strengths, including synthesizing more than 14 years of evidence, and represents what is, to our knowledge, the most comprehensive systematic review and meta-analysis evaluating the use and effectiveness of MCT. This also may be the first to assess distal outcomes, highlighting the apparent benefit of the intervention on negative symptoms, self-esteem, and functioning. Our approach addresses prior meta-analytic inconsistencies (ie, null results of MCT on proximal outcomes)^20,21^; methodological quality was investigated, and the findings were robust to sensitivity analyses.

This study also has limitations. We observed significant heterogeneity of ES values for studies assessing global positive symptoms, delusions, QOL, and functioning, although heterogeneity was not evident at follow-up.

The use of a random-effects model, which assumes that real ES values vary between studies, was implemented to mitigate this limitation.^31^ Publication bias was present for hallucinations, cognitive biases, negative symptoms, self-esteem, and QOL. A publication bias likely exists for cognitive biases; however, a publication bias for the other variables is unlikely given they were never identified in the literature as primary study outcomes. In addition, lower-quality studies had significantly lower ES values for distal outcomes and we could not include well-being in our quantitative review because it was assessed by only 1 study. Furthermore, the number of RCTs reporting a follow-up exceeding 1 year was insufficient to conduct reliable analyses across outcomes, and some moderator analyses were not interpretable owing to small subgroups or those with data not reported. Hence, important moderators and/or delayed effectiveness^53^ perhaps were not captured. Another limitation was noted with all analyses underpowered for QOL, and self-esteem was underpowered in the RCT-only meta-analysis.

## Conclusions

The findings of this systematic review and meta-analysis suggest that MCT is a beneficial and durable low-threshold intervention that can be flexibly delivered at minimal cost in a variety of contexts to individuals with psychotic disorders. Metacognitive training has also been associated with positive outcomes in different patient populations, such as those with borderline personality disorder, depression, and obsessive-compulsive disorder^16,27,91,92^; future meta-analyses might consider investigating MCT as a transdiagnostic treatment. The inclusion of several new high-quality international trials attests to the intervention’s accessibility, adaptability, and cultural sensitivity. These findings provide some evidence to consider MCT in international treatment guidelines and the focus may now shift toward implementation and cost-effectiveness trials in real-world clinical settings. In addition, the COVID-19 pandemic has exacerbated the need for virtual evidence-based psychological intervention delivery, especially among vulnerable populations. It may be useful for future work to also assess the feasibility, acceptability, and effectiveness of MCT as a virtually delivered^93^ intervention.

## Notes

Supplement. **eAppendix 1.** Detailed Methodology and Supplemental Results: Post Hoc Comparisons and Narrative Review
eReferences
**eAppendix 2.** References of Studies Included in the Systematic Review and Meta-analysis **eTable 1.** Systematic Review Search Strategy **eTable 2.** Core Modules of Metacognitive Training (MCT) for Psychosis **eTable 3.** Comprehensive List of Extracted Variables **eTable 4.** Quality Assessment of Included Studies Using the Mixed Methods Appraisal Tool **eTable 5.** Supplemental Description of Included Studies **eTable 6.** Metacognitive Training (MCT) Intervention Characteristics of Included Studies **eFigure 1.** Forest Plots by Outcome for the Pre-Post Comparisons **eTable 7.** List of Excluded Studies and Ongoing Trials **eTable 8.** Effect Sizes of Maintenance Effectiveness by Outcome **eTable 9.** Moderator and Subgroup Analyses of Study, Participant, and Treatment Characteristics on Proximal and Distal Outcomes for the Pre-Post Timepoints Comparison **eTable 10.** Moderator and Subgroup Analyses of Study, Participant, and Treatment Characteristics on Separate Outcomes for the Pre-Post Timepoints Comparison **eFigure 2.** Scatterplot of Publication Year Significantly Moderating Effect Sizes for Hallucinations in Pre-Post Comparison **eTable 11.** Heterogeneity Assessment by Outcome and Timepoints Comparison **eTable 12.** Rosenthal’s Fail-safe N and Tests for Asymmetry of Funnel Plots (Egger Test) by Outcome for the Pre-Post Comparison **eFigure 3.** Funnel Plots for Each Outcome for the Pre-Post Comparison **eTable 13.** Sensitivity Analyses for Proximal and Distal Outcomes Measured at Pre- and Post-intervention **eFigure 4.** Forest Plots by Outcome for the Pre-Post Comparisons Including Only Randomized Clinical Trials **eTable 14.** Effect Size Comparisons Between All Study Designs and RCT-Only Meta-analyses

## References

[1] Owen MJ, Sawa A, Mortensen PB. Schizophrenia. Lancet. 2016;388(10039):86-97. doi:10.1016/S0140-6736(15)01121-6 26777917 PMC4940219

[2] Chong HY, Teoh SL, Wu DB-C, Kotirum S, Chiou C-F, Chaiyakunapruk N. Global economic burden of schizophrenia: a systematic review. Neuropsychiatr Dis Treat. 2016;12:357-373. 26937191 10.2147/NDT.S96649 PMC4762470

[3] National Institute of Mental Health. Schizophrenia. Accessed May 12, 2021. https://www.nimh.nih.gov/health/topics/schizophrenia

[4] Saha S, Chant D, Welham J, McGrath J. A systematic review of the prevalence of schizophrenia. PLoS Med. 2005;2(5):e141. doi:10.1371/journal.pmed.0020141 15916472 PMC1140952

[5] American Psychiatric Association. Diagnostic and Statistical Manual of Mental Disorders (DSM-5). American Psychiatric Publications; 2013.

[6] Moritz S, Woodward TS. Metacognitive training for schizophrenia patients (MCT): a pilot study on feasibility, treatment adherence, and subjective efficacy. German J Psychiatry. 2007;10(3):69-78.

[7] Moritz S, Andreou C, Schneider BC, . Sowing the seeds of doubt: a narrative review on metacognitive training in schizophrenia. Clin Psychol Rev. 2014;34(4):358-366. doi:10.1016/j.cpr.2014.04.004 24866025

[8] Hutton P, Morrison AP, Wardle M, Wells A. Metacognitive therapy in treatment-resistant psychosis: a multiple-baseline study. Behav Cogn Psychother. 2014;42(2):166-185. doi:10.1017/S1352465812001026 23286558

[9] Wells A. Metacognitive Therapy for Anxiety and Depression. Guilford Press; 2011.

[10] Lysaker PH, Klion RE. Recovery, Meaning-Making, and Severe Mental Illness: A Comprehensive Guide to Metacognitive Reflection and Insight Therapy. Routledge; 2017. doi:10.4324/9781315447001

[11] Andreou C, Wittekind CE, Fieker M, . Individualized metacognitive therapy for delusions: a randomized controlled rater-blind study. J Behav Ther Exp Psychiatry. 2017;56:144-151. doi:10.1016/j.jbtep.2016.11.013 27919404

[12] Moritz S, Woodward TS, Balzan R. Is metacognitive training for psychosis effective? Expert Rev Neurother. 2016;16(2):105-107. doi:10.1586/14737175.2016.1135737 26694013

[13] Moritz S, Veckenstedt R, Randjbar S, Vitzthum F, Woodward TS. Antipsychotic treatment beyond antipsychotics: metacognitive intervention for schizophrenia patients improves delusional symptoms. Psychol Med. 2011;41(9):1823-1832. doi:10.1017/S0033291710002618 21275083

[14] Eichner C, Berna F. Acceptance and efficacy of metacognitive training (MCT) on positive symptoms and delusions in patients with schizophrenia: a meta-analysis taking into account important moderators. Schizophr Bull. 2016;42(4):952-962. doi:10.1093/schbul/sbv225 26748396 PMC4903058

[15] Liu YC, Tang CC, Hung TT, Tsai PC, Lin MF. The efficacy of metacognitive training for delusions in patients with schizophrenia: a meta-analysis of randomized controlled trials informs evidence-based practice. Worldviews Evid Based Nurs. 2018;15(2):130-139. doi:10.1111/wvn.12282 29489070

[16] Philipp R, Kriston L, Lanio J, . Effectiveness of metacognitive interventions for mental disorders in adults—a systematic review and meta-analysis (METACOG). Clin Psychol Psychother. 2019;26(2):227-240. doi:10.1002/cpp.2345 30456821

[17] Sauvé G, Lavigne KM, Pochiet G, Brodeur MB, Lepage M. Efficacy of psychological interventions targeting cognitive biases in schizophrenia: a systematic review and meta-analysis. Clin Psychol Rev. 2020;78:101854. doi:10.1016/j.cpr.2020.101854 32361339

[18] Jiang J, Zhang L, Zhu Z, Li W, Li C. Metacognitive training for schizophrenia: a systematic review. Shanghai Arch Psychiatry. 2015;27(3):149-157. 26300597 10.11919/j.issn.1002-0829.215065 PMC4526827

[19] Lopez-Morinigo J-D, Ajnakina O, Martínez AS-E, . Can metacognitive interventions improve insight in schizophrenia spectrum disorders? a systematic review and meta-analysis. Psychol Med. 2020;50(14):2289-2301. doi:10.1017/S0033291720003384 33050956 PMC7610184

[20] van Oosterhout B, Smit F, Krabbendam L, Castelein S, Staring AB, van der Gaag M. Metacognitive training for schizophrenia spectrum patients: a meta-analysis on outcome studies. Psychol Med. 2016;46(1):47-57. doi:10.1017/S0033291715001105 26190517

[21] Burlingame GM, Svien H, Hoppe L, Hunt I, Rosendahl J. Group therapy for schizophrenia: a meta-analysis. Psychotherapy (Chic). 2020;57(2):219-236. doi:10.1037/pst0000293 32478561

[22] Moritz S, Werner D, Menon M, Balzan R, Woodward T. Jumping to negative conclusions–a case of study-gathering bias? Psychol Med. 2016;46(1):59-61. doi:10.1017/S0033291715002068 26490783

[23] van Oosterhout B, Smit F, Krabbendam L, Castelein S, Staring AB, van der Gaag M. Letter to the Editor: Should we focus on quality or quantity in meta-analyses? Psychol Med. 2016;46(9):2003-2005. doi:10.1017/S003329171600009X 26888290

[24] Moritz S, Turner DT, Bechdolf A, . Group therapy for schizophrenia: why Burlingame et al. should redo their meta-analysis. Psychotherapy (Chic). Published online December 23, 2021. 10.1037/pst0000401 35666916

[25] Chen Q, Sang Y, Ren L, . Metacognitive training: a useful complement to community-based rehabilitation for schizophrenia patients in China. BMC Psychiatry. 2021;21(1):38. doi:10.1186/s12888-021-03039-y 33441093 PMC7805146

[26] Acuña V, Otto A, Cavieres A, Villalobos H. Efficacy of metacognitive training in a Chilean sample of people with schizophrenia [Spanish]. Rev Colomb Psiquiatr (Engl Ed). 2021;S0034-7450(21)00030-5. 36369153 10.1016/j.rcpeng.2020.12.002

[27] Tanoue H, Yoshinaga N, Hayashi Y, Ishikawa R, Ishigaki T, Ishida Y. Clinical effectiveness of metacognitive training as a transdiagnostic program in routine clinical settings: a prospective, multicenter, single-group study. Jpn J Nurs Sci. 2021;18(2):e12389. doi:10.1111/jjns.12389 33174673

[28] Page MJ, McKenzie JE, Bossuyt PM, . The PRISMA 2020 statement: an updated guideline for reporting systematic reviews. BMJ. 2021;372:n71. doi:10.1136/bmj.n71 33782057 PMC8005924

[29] Hong QN, Fàbregues S, Bartlett G, . The Mixed Methods Appraisal Tool (MMAT) version 2018 for information professionals and researchers. Educ Inf. 2018;34(4):285-291. doi:10.3233/EFI-180221

[30] Shrier I, Boivin J-F, Steele RJ, . Should meta-analyses of interventions include observational studies in addition to randomized controlled trials? a critical examination of underlying principles. Am J Epidemiol. 2007;166(10):1203-1209. doi:10.1093/aje/kwm189 17712019

[31] Borenstein M, Hedges LV, Higgins JPT, Rothstein HR. Introduction to Meta-analysis. John Wiley & Sons, Inc; 2011.

[32] Efthimiou O, Mavridis D, Debray TP, . GetReal Work Package 4: combining randomized and non-randomized evidence in network meta-analysis. Stat Med. 2017;36(8):1210-1226. doi:10.1002/sim.7223 28083901

[33] Aghotor J, Pfueller U, Moritz S, Weisbrod M, Roesch-Ely D. Metacognitive training for patients with schizophrenia (MCT): feasibility and preliminary evidence for its efficacy. J Behav Ther Exp Psychiatry. 2010;41(3):207-211. doi:10.1016/j.jbtep.2010.01.004 20167306

[34] Balzan RP, Delfabbro PH, Galletly CA, Woodward TS. Metacognitive training for patients with schizophrenia: preliminary evidence for a targeted, single-module programme. Aust N Z J Psychiatry. 2014;48(12):1126-1136. doi:10.1177/0004867413508451 24159051

[35] Balzan RP, Mattiske JK, Delfabbro P, Liu D, Galletly C. Individualized metacognitive training (MCT+) reduces delusional symptoms in psychosis: a randomized clinical trial. Schizophr Bull. 2019;45(1):27-36. doi:10.1093/schbul/sby152 30376124 PMC6293215

[36] Briki M, Vandel P, Haffen E, Sechter D. Metacognition training for schizophrenia: a French pilot study. J Neuropsychiatry Clin Neurosci. 2014;26(2):E32-E33. doi:10.1176/appi.neuropsych.13040090 24763784

[37] Briki M, Monnin J, Haffen E, . Metacognitive training for schizophrenia: a multicentre randomised controlled trial. Schizophr Res. 2014;157(1-3):99-106. doi:10.1016/j.schres.2014.06.005 24972754

[38] de Pinho LMG, Sequeira CADC, Sampaio FMC, Rocha NB, Ozaslan Z, Ferre-Grau C. Assessing the efficacy and feasibility of providing metacognitive training for patients with schizophrenia by mental health nurses: a randomized controlled trial. J Adv Nurs. 2021;77(2):999-1012. doi:10.1111/jan.14627 33222210

[39] Erawati E, Keliat BA, Helena N, Hamid A. The influence of metacognitive training on delusion severity and metacognitive ability in schizophrenia. J Psychiatr Ment Health Nurs. 2014;21(9):841-847. doi:10.1111/jpm.12130 24548341

[40] Favrod J, Maire A, Bardy S, Pernier S, Bonsack C. Improving insight into delusions: a pilot study of metacognitive training for patients with schizophrenia. J Adv Nurs. 2011;67(2):401-407. doi:10.1111/j.1365-2648.2010.05470.x 20955184

[41] Favrod J, Rexhaj S, Bardy S, . Sustained antipsychotic effect of metacognitive training in psychosis: a randomized-controlled study. Eur Psychiatry. 2014;29(5):275-281. doi:10.1016/j.eurpsy.2013.08.003 24176646

[42] Ferwerda J, de Boer K, van der Gaag M. Metacognitieve training voor patiënten met een psychotische kwetsbaarheid. Directieve Therapie. 2010;30(4):263-279. doi:10.1007/s12433-010-0240-y

[43] Fujii K, Kobayashi M, Funasaka K, Kurokawa S, Hamagami K. Effectiveness of metacognitive training for long-term hospitalized patients with schizophrenia: a pilot study with a crossover design. Asian J Occupational Ther. 2021;17(1):45-52. doi:10.11596/asiajot.17.45

[44] Gawęda Ł, Krężołek M, Olbryś J, Turska A, Kokoszka A. Decreasing self-reported cognitive biases and increasing clinical insight through meta-cognitive training in patients with chronic schizophrenia. J Behav Ther Exp Psychiatry. 2015;48:98-104. doi:10.1016/j.jbtep.2015.02.002 25775947

[45] Ishikawa R, Ishigaki T, Shimada T, . The efficacy of extended metacognitive training for psychosis: a randomized controlled trial. Schizophr Res. 2020;215:399-407. doi:10.1016/j.schres.2019.08.006 31471248

[46] Kowalski J, Pankowski D, Lew-Starowicz M, Gawęda Ł. Do specific metacognitive training modules lead to specific cognitive changes among patients diagnosed with schizophrenia? a single module effectiveness pilot study. Psychosis. 2017;9(3):254-259. doi:10.1080/17522439.2017.1300186

[47] Kumar D, Zia Ul Haq M, Dubey I, . Effect of meta-cognitive training in the reduction of positive symptoms in schizophrenia. Eur J Psychotherapy Counselling. 2010;12(2):149-158. doi:10.1080/13642537.2010.488875

[48] Kumar D, Rao MG, Raveendranathan D, Venkatasubramanian G, Varambally S, Gangadhar BN. Metacognitive training for delusion in treatment-resistant schizophrenia. Clin Schizophr Relat Psychoses. 2015;9(1):40-43. doi:10.3371/CSRP.KURA.031513 23518783

[49] Kuokkanen R, Lappalainen R, Repo-Tiihonen E, Tiihonen J. Metacognitive group training for forensic and dangerous non-forensic patients with schizophrenia: a randomised controlled feasibility trial. Crim Behav Ment Health. 2014;24(5):345-357. doi:10.1002/cbm.1905 24619628

[50] Kuokkanen R, Aho-Mustonen K, Muotka J, Lappalainen R, Tiihonen J. A pilot study of group administered metacognitive training (MCT) for schizophrenia patients in a high-security forensic setting: subjective training success and health-related quality of life. J Forensic Psychol Pract. 2015;15(4):344-362. doi:10.1080/15228932.2015.1053546

[51] Moritz S, Kerstan A, Veckenstedt R, . Further evidence for the efficacy of a metacognitive group training in schizophrenia. Behav Res Ther. 2011;49(3):151-157. doi:10.1016/j.brat.2010.11.010 21276962

[52] Moritz S, Veckenstedt R, Bohn F, . Complementary group metacognitive training (MCT) reduces delusional ideation in schizophrenia. Schizophr Res. 2013;151(1-3):61-69. doi:10.1016/j.schres.2013.10.007 24183707

[53] Moritz S, Veckenstedt R, Andreou C, . Sustained and “sleeper” effects of group metacognitive training for schizophrenia: a randomized clinical trial. JAMA Psychiatry. 2014;71(10):1103-1111. doi:10.1001/jamapsychiatry.2014.1038 25103718

[54] Moritz S, Menon M, Andersen D, Woodward TS, Gallinat J. Moderators of symptomatic outcome in metacognitive training for psychosis (MCT): who benefits and who does not? Cognit Ther Res. 2018;42(1):80-91. doi:10.1007/s10608-017-9868-3

[55] Naughton M, Nulty A, Abidin Z, Davoren M, O’Dwyer S, Kennedy HG. Effects of group metacognitive training (MCT) on mental capacity and functioning in patients with psychosis in a secure forensic psychiatric hospital: a prospective-cohort waiting list controlled study. BMC Res Notes. 2012;5(1):302. doi:10.1186/1756-0500-5-302 22709616 PMC3406956

[56] Ochoa S, López-Carrilero R, Barrigón ML, ; Spanish Metacognition Study Group. Randomized control trial to assess the efficacy of metacognitive training compared with a psycho-educational group in people with a recent-onset psychosis. Psychol Med. 2017;47(9):1573-1584. doi:10.1017/S0033291716003421 28166848

[57] Salas-Sender M, López-Carrilero R, Barajas A, ; The Spanish Metacognition Study Group. Gender differences in response to metacognitive training in people with first-episode psychosis. J Consult Clin Psychol. 2020;88(6):516-525. doi:10.1037/ccp0000468 31855037

[58] Ochoa S, Lopez-Carrilero R, Barrigon ML, . S34. Effectiveness of individual metacognitive training (MCT+) in first-episode psychosis. Schizophr Bull. 2020;46(suppl 1):S44. doi:10.1093/schbul/sbaa031.100

[59] Park S, Lee HK, Kim H. Effects of a Korean version of the metacognitive training program for outpatients with schizophrenia on theory of mind, positive symptoms, and interpersonal relationships. Behav Cogn Psychother. 2020;48(1):14-24. doi:10.1017/S1352465819000560 31625497

[60] Pos K, Meijer CJ, Verkerk O, Ackema O, Krabbendam L, de Haan L. Metacognitive training in patients recovering from a first psychosis: an experience sampling study testing treatment effects. Eur Arch Psychiatry Clin Neurosci. 2018;268(1):57-64. doi:10.1007/s00406-017-0833-7 28828697 PMC5778181

[61] Schneider BC, Cludius B, Lutz W, Moritz S, Rubel JA. An investigation of module-specific effects of metacognitive training for psychosis. Z Psychol Z Angew Psychol. 2018;226(3). doi:10.1027/2151-2604/a000336

[62] Shan X, Liao R, Ou Y, . Increased regional homogeneity modulated by metacognitive training predicts therapeutic efficacy in patients with schizophrenia. Eur Arch Psychiatry Clin Neurosci. 2021;271(4):783-798. doi:10.1007/s00406-020-01119-w 32215727 PMC8119286

[63] Simón-Expósito M, Felipe-Castaño E. Effects of metacognitive training on cognitive insight in a sample of patients with schizophrenia. Int J Environ Res Public Health. 2019;16(22):4541. doi:10.3390/ijerph16224541 31744146 PMC6888430

[64] So SH-W, Chan AP, Chong CS-Y, . Metacognitive training for delusions (MCTd): effectiveness on data-gathering and belief flexibility in a Chinese sample. Front Psychol. 2015;6:730. doi:10.3389/fpsyg.2015.00730 26124726 PMC4467068

[65] Ho-Wai So S, Hoi-Kei Chan G, Kit-Wa Wong C, . A randomised controlled trial of metacognitive training for psychosis, depression, and belief flexibility. J Affect Disord. 2021;279:388-397. doi:10.1016/j.jad.2020.09.126 33099054

[66] Ussorio D, Giusti L, Wittekind CE, . Metacognitive training for young subjects (MCT young version) in the early stages of psychosis: is the duration of untreated psychosis a limiting factor? Psychol Psychother. 2016;89(1):50-65. doi:10.1111/papt.12059 25799999

[67] van Oosterhout B, Krabbendam L, de Boer K, . Metacognitive group training for schizophrenia spectrum patients with delusions: a randomized controlled trial. Psychol Med. 2014;44(14):3025-3035. doi:10.1017/S0033291714000555 25066223

[68] Yildiz M, Özaslan Z, İncedere A, Kircali A, Kiras F, İpçi K. The effect of psychosocial skills training and metacognitive training on social and cognitive functioning in schizophrenia. Noro Psikiyatr Ars. 2018;56(2):139-143. doi:10.29399/npa.23095 31223248 PMC6563869

[69] Zalzala A, Wardwell P, Petrik T, . F121. Metacognitive training (MCT) to improve insight and work outcome in schizophrenia. Schizophr Bull. 2019;45(suppl 2):S299-S300. doi:10.1093/schbul/sbz018.533

[70] Higgins J, Thomas J, Chandler J, . Cochrane Handbook for Systematic Reviews of Interventions, version 6.2. 2021. Accessed August 19, 2021. http://www.training.cochrane.org/handbook

[71] Rosenthal R. Meta-analytic Procedures for Social Research. Vol 6. Rev. ed. Sage Publications Inc; 1991. doi:10.4135/9781412984997

[72] Egger M, Davey Smith G, Schneider M, Minder C. Bias in meta-analysis detected by a simple, graphical test. BMJ. 1997;315(7109):629-634. doi:10.1136/bmj.315.7109.629 9310563 PMC2127453

[73] Rosenthal R. The file drawer problem and tolerance for null results. Psychol Bull. 1979;86(3):638. doi:10.1037/0033-2909.86.3.638

[74] Cochran WG. The combination of estimates from different experiments. Biometrics. 1954;10(1):101-129. doi:10.2307/3001666

[75] Higgins JP, Thompson SG, Deeks JJ, Altman DG. Measuring inconsistency in meta-analyses. BMJ. 2003;327(7414):557-560. doi:10.1136/bmj.327.7414.557 12958120 PMC192859

[76] Page MJ, Moher D, Bossuyt PM, . PRISMA 2020 explanation and elaboration: updated guidance and exemplars for reporting systematic reviews. BMJ. 2021;372:n160. Published online March 29, 2021. doi:10.1136/bmj.n160 33781993 PMC8005925

[77] Kesting M-L, Lincoln TM. The relevance of self-esteem and self-schemas to persecutory delusions: a systematic review. Compr Psychiatry. 2013;54(7):766-789. doi:10.1016/j.comppsych.2013.03.002 23684547

[78] Lutgens D, Gariepy G, Malla A. Psychological and psychosocial interventions for negative symptoms in psychosis: systematic review and meta-analysis. Br J Psychiatry. 2017;210(5):324-332. doi:10.1192/bjp.bp.116.197103 28302699

[79] Foussias G, Agid O, Fervaha G, Remington G. Negative symptoms of schizophrenia: clinical features, relevance to real world functioning and specificity versus other CNS disorders. Eur Neuropsychopharmacol. 2014;24(5):693-709. doi:10.1016/j.euroneuro.2013.10.017 24275699

[80] Roe D. A prospective study on the relationship between self-esteem and functioning during the first year after being hospitalized for psychosis. J Nerv Ment Dis. 2003;191(1):45-49. doi:10.1097/00005053-200301000-00008 12544599

[81] Galderisi S, Rossi A, Rocca P, ; Italian Network For Research on Psychoses. The influence of illness-related variables, personal resources and context-related factors on real-life functioning of people with schizophrenia. World Psychiatry. 2014;13(3):275-287. doi:10.1002/wps.20167 25273301 PMC4219069

[82] Sitko K, Bewick BM, Owens D, Masterson C. Meta-analysis and meta-regression of cognitive behavioral therapy for psychosis (CBTp) across time: the effectiveness of CBTp has improved for delusions. Schizophrenia Bull Open. 2020;1(1):sgaa023. doi:10.1093/schizbullopen/sgaa023

[83] Laws KR, Darlington N, Kondel TK, McKenna PJ, Jauhar S. Cognitive behavioural therapy for schizophrenia—outcomes for functioning, distress and quality of life: a meta-analysis. BMC Psychol. 2018;6(1):32. doi:10.1186/s40359-018-0243-2 30016999 PMC6050679

[84] Vita A, Barlati S, Ceraso A, . Effectiveness, core elements, and moderators of response of cognitive remediation for schizophrenia: a systematic review and meta-analysis of randomized clinical trials. JAMA Psychiatry. 2021;78(8):848-858. doi:10.1001/jamapsychiatry.2021.0620 33877289 PMC8058696

[85] Wykes T, Huddy V, Cellard C, McGurk SR, Czobor P. A meta-analysis of cognitive remediation for schizophrenia: methodology and effect sizes. Am J Psychiatry. 2011;168(5):472-485. doi:10.1176/appi.ajp.2010.10060855 21406461

[86] Cella M, Preti A, Edwards C, Dow T, Wykes T. Cognitive remediation for negative symptoms of schizophrenia: a network meta-analysis. Clin Psychol Rev. 2017;52:43-51. doi:10.1016/j.cpr.2016.11.009 27930934

[87] Moritz S, Göritz AS, Balzan RP, Gawęda Ł, Kulagin SC, Andreou C. A new paradigm to measure probabilistic reasoning and a possible answer to the question why psychosis-prone individuals jump to conclusions. J Abnorm Psychol. 2017;126(4):406-415. doi:10.1037/abn0000262 28277733

[88] Eack SM, Newhill CE. Psychiatric symptoms and quality of life in schizophrenia: a meta-analysis. Schizophr Bull. 2007;33(5):1225-1237. doi:10.1093/schbul/sbl071 17204532 PMC2632363

[89] Schrank B, Riches S, Coggins T, Tylee A, Slade M. From objectivity to subjectivity: conceptualization and measurement of well-being in mental health. Neuropsychiatry (London). 2013;3(5):525-534. doi:10.2217/npy.13.58

[90] Warner R. Recovery from schizophrenia and the recovery model. Curr Opin Psychiatry. 2009;22(4):374-380. doi:10.1097/YCO.0b013e32832c920b 19417668

[91] Schilling L, Moritz S, Kriston L, Krieger M, Nagel M. Efficacy of metacognitive training for patients with borderline personality disorder: preliminary results. Psychiatry Res. 2018;262:459-464. doi:10.1016/j.psychres.2017.09.024 28927866

[92] Jelinek L, Van Quaquebeke N, Moritz S. Cognitive and metacognitive mechanisms of change in metacognitive training for depression. Sci Rep. 2017;7(1):3449. doi:10.1038/s41598-017-03626-8 28615651 PMC5471213

[93] Mendelson D, Thibaudeau É, Sauvé G, . Remote group therapies for cognitive health in schizophrenia-spectrum disorders: feasible, acceptable, engaging. Schizophr Res Cogn. Published online December 6, 2021. doi:10.1016/j.scog.2021.100230 PMC8861418 35242604

