# Supplementary material for: Immediate and Sustained Outcomes and Moderators Associated With Metacognitive Training for Psychosis: A Systematic Review and Meta-analysis
Source: JAMA Psychiatry. 2022 Mar 23;79(5):417–29. doi: 10.1001/jamapsychiatry.2022.0277 (PMC8943641; doi:10.1001/jamapsychiatry.2022.0277)
Supplement: Supplement. — eAppendix 1. Detailed Methodology and Supplemental Results: Post Hoc Comparisons and Narrative Review eReferences eAppendix 2. References of Studies Included in the Systematic Review and Meta-analysis eTable 1. Systematic Review Search Strategy eTable 2. Core Modules of Metacognitive Training (MCT) for Psychosis eTable 3. Comprehensive List of Extracted Variables eTable 4. Quality Assessment of Included Studies Using the Mixed Methods Appraisal Tool eTable 5. Supplemental Description of Included Studies eTable 6. Metacognitive Training (MCT) Intervention Characteristics of Included Studies eFigure 1. Forest Plots by Outcome for the Pre-Post Comparisons eTable 7. List of Excluded Studies and Ongoing Trials eTable 8. Effect Sizes of Maintenance Effectiveness by Outcome eTable 9. Moderator and Subgroup Analyses of Study, Participant, and Treatment Characteristics on Proximal and Distal Outcomes for the Pre-Post Timepoints Comparison eTable 10. Moderator and Subgroup Analyses of Study, Participant, and Treatment Characteristics on Separate Outcomes for the Pre-Post Timepoints Comparison eFigure 2. Scatterplot of Publication Year Significantly Moderating Effect Sizes for Hallucinations in Pre-Post Comparison eTable 11. Heterogeneity Assessment by Outcome and Timepoints Comparison eTable 12. Rosenthal’s Fail-safe N and Tests for Asymmetry of Funnel Plots (Egger Test) by Outcome for the Pre-Post Comparison eFigure 3. Funnel Plots for Each Outcome for the Pre-Post Comparison eTable 13. Sensitivity Analyses for Proximal and Distal Outcomes Measured at Pre- and Post-intervention eFigure 4. Forest Plots by Outcome for the Pre-Post Comparisons Including Only Randomized Clinical Trials eTable 14. Effect Size Comparisons Between All Study Designs and RCT-Only Meta-analyses [file jamapsychiatry-e220277-s001.pdf]

# Supplementary Online Content

Penney D, Sauvé G, Mendelson D, Thibaudeau É, Moritz S, Lepage M. Immediate and sustained outcomes and moderators associated with metacognitive training for psychosis: a systematic review and meta-analysis. *JAMA Psychiatry*. Published online March 23, 2022. doi:10.1001/jamapsychiatry.2022.0277


**eAppendix 1.** Detailed Methodology and Supplemental Results: Post Hoc Comparisons and Narrative Review

**eReferences**

**eAppendix 2.** References of Studies Included in the Systematic Review and Meta-analysis

**eTable 1.** Systematic Review Search Strategy

**eTable 2.** Core Modules of Metacognitive Training (MCT) for Psychosis

**eTable 3.** Comprehensive List of Extracted Variables

**eTable 4.** Quality Assessment of Included Studies Using the Mixed Methods Appraisal Tool

**eTable 5.** Supplemental Description of Included Studies

**eTable 6.** Metacognitive Training (MCT) Intervention Characteristics of Included Studies

**eFigure 1.** Forest Plots by Outcome for the Pre-Post Comparisons

**eTable 7.** List of Excluded Studies and Ongoing Trials

**eTable 8.** Effect Sizes of Maintenance Effectiveness by Outcome

**eTable 9.** Moderator and Subgroup Analyses of Study, Participant, and Treatment Characteristics on Proximal and Distal Outcomes for the Pre-Post Timepoints Comparison

**eTable 10.** Moderator and Subgroup Analyses of Study, Participant, and Treatment Characteristics on Separate Outcomes for the Pre-Post Timepoints Comparison

**eFigure 2.** Scatterplot of Publication Year Significantly Moderating Effect Sizes for Hallucinations in Pre-Post Comparison

**eTable 11.** Heterogeneity Assessment by Outcome and Timepoints Comparison

**eTable 12.** Rosenthal's Fail-safe N and Tests for Asymmetry of Funnel Plots (Egger Test) by Outcome for the Pre-Post Comparison

**eFigure 3.** Funnel Plots for Each Outcome for the Pre-Post Comparison

**eTable 13.** Sensitivity Analyses for Proximal and Distal Outcomes Measured at Pre- and Post-intervention

**eFigure 4.** Forest Plots by Outcome for the Pre-Post Comparisons Including Only Randomized Clinical Trials

**eTable 14.** Effect Size Comparisons Between All Study Designs and RCT-Only Meta-analyses


This supplementary material has been provided by the authors to give readers additional information about their work

**eAppendix 1. Detailed methods and supplemental results: post hoc comparisons and narrative review**

## Methods

The study protocol was registered on the PROSPERO database (CRD 42021259291, available at https://www.crd.york.ac.uk/prospero/display_record.php?RecordID=259291) and the PRISMA 2020 guidelines were followed.[1] The PRISMA checklist is displayed in eAppendix A. Modifications to the registered protocol include (1) the removal of causal language from the title and study objectives (i.e., the term “efficacy” has been replaced with “effectiveness”); (2) the addition of moderator analyses for study design (randomized controlled trials (RCTs), non-RCTs) and diagnosis (% schizophrenia spectrum disorders); and (3) follow-up analyses assessing the effectiveness of MCT on proximal and distal outcomes including only RCTs.

*Search Strategy*

The literature search strategy was developed in collaboration with a university librarian and is available in eTable 1. The search was conducted on June 3, 2021, using the following study registers and bibliographic databases: Cochrane Central Register of Controlled Trials (CENTRAL), CINAHL (EBSCO), PubMed, Embase (Ovid), MEDLINE (Ovid), PsycINFO (Ovid), Social Work Abstracts (Ovid), and Web of Science electronic bibliographic databases. Grey literature was searched using OpenGrey, ProQuest Dissertations, and Social Science Research Network eLibrary. The search strategy is presented in eTable 1. Searches were restricted to records published after 2007, the year the first paper on metacognitive training for psychosis (MCT) was published.[2] The search did not include any language restrictions. The research team was capable of screening English, French, German, and Spanish reports. When a report needed translation to verify its eligibility or extract data (n = 2) it was translated using DeepL Translator. The search was not restricted based on study design. The bibliographies of

retrieved systematic reviews/meta-analyses and included reports were screened for additional reports. The co-developer of MCT (S. Moritz) verified the comprehensiveness of the search results and to mitigate conflict of interest, was not involved in any activities pertaining to study or report selection, data extraction, quality control, nor analyses. Search updates were performed via automatic alert for the Web of Science database until data analyses were performed on September 10, 2021.

Search results were managed using Covidence®. The flowchart of report selection is presented in manuscript Figure 1. Note that per the PRISMA 2020 glossary of terms[1], a *report* is a document providing information about a particular study (e.g., a scientific paper); a *record* is the title and/or abstract of a report that has been indexed in a database or website; and a *study* is a unique investigation or clinical trial that includes a defined group of participants and at least one intervention or outcome. As such, a *study* may have several associated reports. A total of 1045 records were initially identified. After duplicate removal (n=526), all records were screened based on title and abstract by authors DP and DM. Discrepancies (n=9) were resolved by author ÉT until consensus was reached (based on majority agreement). The full-text of 294 articles were assessed for eligibility by DP and DM. Each rater screened the first 100 records and then met to resolve discrepancies (n=7) by consensus (collaborative review of inclusion/exclusion criteria until agreement was reached). The remaining records were split equally and screened independently. Included reports were published in peer-reviewed journals; books and conference abstracts were excluded unless supplemental data was retrieved (for conference abstracts) from the report author. Studies had to include participants diagnosed with a schizophrenia spectrum or related psychotic disorder. This included schizophrenia spectrum and other psychotic disorders per the categories and criteria identified in the DSM-5.[3] Given the inclusion of studies

representing real-world clinical realities, diagnoses of depression with psychotic features, dissociative disorder, and bipolar I were tolerated (when representing a minority of participants). These diagnoses were subsumed under “other psychotic disorder” for the purpose of moderator analyses. A list of all included diagnoses is available in eTable 5. There were no restrictions based on sex, gender, ethnicity, or age. Studies also had to administer the original version or adaptations of MCT for psychosis (see eTable 2 for core modules and eTable 6 for intervention characteristics). Acceptable adaptations included variability in number of sessions, number of sessions per week, and session duration. Both individual and group formats were considered.

Data extraction was performed using a template developed by author GS. The template was then piloted with GS, DP, and DM. Three reviewers (DP, DM, ÉT) then extracted the data; reports were randomly distributed among reviewers. Author GS reviewed a random selection of 10% of the extracted data for the purpose of quality control. Discrepancies were resolved via consensus (i.e., majority agreement) among the 4 reviewers. When relevant data (e.g., means and standard deviations) were not reported in the study/report, corresponding authors were contacted via e-mail. Relevant data for 12 reports were obtained using this method. Data from the most recent report were selected when multiple reports corresponded to the same study. Corresponding authors were contacted to resolve inconsistencies across reports as applicable.

*Proximal and Distal Outcome Measures*

Only reports that investigated selected proximal (positive symptoms, delusions, hallucinations, cognitive biases) and/or distal outcomes (self-esteem, negative symptoms, quality of life (QoL), wellbeing, social and global functioning) were included. Proximal outcomes included the direct treatment targets of MCT. Distal outcomes were identified as either (1) important secondary targets of the intervention (i.e., self-esteem); (2) person-centered outcomes (i.e., QoL, wellbeing,

functioning) important to long-term outcome;[4] and (3) negative symptoms (e.g., anhedonia, avolition, social withdrawal), which are important predictors of person-centered outcomes.[5,6] A comprehensive list of extracted variables is available in eTable 3. All measures and timepoints compatible with selected outcomes were sought.

*Methodological Quality Assessment*

Study risk of bias assessment was independently performed by authors DP and DM using the Mixed Methods Appraisal Tool (MMAT; version 2018).[7] MMAT methodological quality criteria and results are presented in  eTable 4. Interrater agreement on 10% of randomly allocated assessments was 85.71%. Disagreements were resolved between the two authors following examination and discussion of the MMAT criteria. Incomplete outcome data was qualified (i.e., yes response on the MMAT) when attrition rates exceeded 20% in at least one participant group (treatment, control groups). This criterion was previously used in meta-analyses assessing cognitive behavioral therapy for psychosis[8] and MCT.[9] When incomplete outcome data were reported, reviewers noted whether intention-to-treat analyses were employed.

*Data Synthesis Procedure*

Selected outcomes were synthesized with separate meta-analyses using Comprehensive Meta-Analysis (version 3, Biostat). Reports were eligible for quantitative synthesis if they reported sample sizes, means and standard deviations, percentages and/or effect sizes with measure of variance (e.g., confidence intervals), for pre- and post-treatment outcome measures. Meta-analyses were not limited to randomized controlled trials (RCTs); the rationale was guided by Shrier et al.,[10] Borenstein et al., [11] and Efthimiou et al.[12] who suggest that if studies address a common question (treatment effects on the same outcomes), limiting meta-analyses to RCTs is arbitrary; the process of randomization does not infer study quality (the extent that a study yields

an unbiased estimate of effect). Importantly, meta-analyses based on non-RCTs typically yield similar effect size estimates to those assessing RCTs.[10] Having said that, we did assess study design as a moderator of MCT effectiveness, and ran separate meta-analyses on proximal and distal outcomes using only RCTs (analyses included 31 studies/33 reports) to verify effectiveness of the intervention on proximal and distal outcomes. Details on studies/reports included in the systematic review but ineligible for the quantitative synthesis (n=6) are displayed in manuscript Table 1 and results were outlined in a narrative review. To conduct meta-analyses, Hedges' *g* effect sizes were computed using the extracted data and interpreted as small ($g = 0.2$), medium ($g = 0.5$) and large ($g = 0.8$).[13] We selected this parameter to correct for the overestimating bias associated with the Cohen's *d* effect size known to occur for small sample studies.[11] Effect sizes were pooled for reports assessing multiple follow-up timepoints or scales measuring the same outcome.

*Moderator Analyses*

Subgroup analyses were conducted to explore the possible causes of expected heterogeneity. Subgroups and Q-statistics with significance tests were used for the following categorical variables: risk of bias (number of "yes" ratings ranging from 1 to 5, 1 being lowest quality, 5 being highest), type of analyses (intention-to-treat, per-protocol), study design (RCTs, non-RCTs), comparator type (none, active, passive), intervention delivery format (group, individual), manual adherence (yes, no), facilitator training (yes, no), facilitator credentials (general practitioner, graduate student, nurse, occupational therapist, psychiatrist, psychologist), number of MCT sessions (>8, 1 cycle, 2 cycles, <16, other), and gender (note: no gender-related data, i.e., data including non-binary, other, etc. categories, were reported). Post-hoc comparisons were performed for analyses with a significant omnibus test and more than 2 subgroups. Meta-

regression analyses using a random-effects model were performed for continuous variables, namely diagnosis (% schizophrenia spectrum disorders), year of publication, age, sex (% male), medication (chlorpromazine equivalent), and duration of illness.

*Certainty of Evidence*

Sensitivity analyses (using r values of 0.50; 0.70; 0.90) were performed to estimate the correlations between pre- and post-treatment scores when they were not reported.[14] When overall results were robust to the use of imputed correlations, a conservative value of 0.7 was employed, as recommended by Rosenthal.[15]

For each selected outcome, the potential for publication bias was verified via visual examination of the funnel plot by GS, Egger's asymmetry test, and Rosenthal's [16,17] fail-safe N. The first two indicators statistically and graphically provide estimates of whether the effect sizes of individual studies are evenly distributed around the mean effect size.[18] The later indicator denotes the required number of studies to rule out a significant overall effect size.[16]

To estimate heterogeneity of effect sizes, the Cochran's Q-statistic[19] and the $I^2$ index[20] were calculated. A Cochran's Q-statistic *p*-value below 0.1 was interpreted as indicating heterogeneity and $I^2$ indexes of 25%, 50% and 75% were construed as low, moderate, and strong heterogeneity, respectively.[21] A random-effects model was used given the anticipated differences between studies regarding test administration and MCT intervention features (e.g., individual vs group format).[22]

**Supplemental Results**

*Post hoc comparisons of significant moderator analysis for hallucinations*.

Post hoc comparisons were performed for subgroup analyses with a significant omnibus test. A significant Q-statistic was obtained for the number of MCT sessions on positive symptoms (Q4 =

10.03, $p < .05$) and post hoc comparisons revealed that studies providing less than 8 sessions ($g$ = 0.97, 6 reports) had a significantly higher effect size than studies providing 2 MCT cycles ($g$ = 0.22, 6 reports). However, effect sizes were not statistically different between studies providing 1or 2 cycles of MCT. Given the high discrepancies between studies testing the effectiveness of less than 8 MCT sessions, these results should be interpreted with caution. We observed other significant omnibus tests but did not conduct post hoc comparisons because there were too few studies in each subgroup, or the comparison was not clinically relevant (e.g., “data not reported” subgroups). The same caution is also warranted when interpreting the results of meta-regression analyses with less than 10 studies.

*Narrative Review*

Six studies/reports investigated the efficacy/effect of MCT on our selected outcomes but could not be included in our meta-analysis. Their characteristics are presented in manuscript Tables 1-3, and eTable 5. A summary of findings is detailed below.

An early study by Aghotor et al.[23] verified the feasibility and preliminary efficacy of MCT. They observed numerical improvements in positive symptoms (non-significant medium effect size, $d$ = 0.43) and cognitive biases (non-significant small-to-medium effect size, $d$ = 0.31). In a more fine-grained analysis, Schneider et al.[24] investigated the effects of MCT after each module. They found the largest improvement in positive symptoms (small effect size) following the theory of mind II module, and the greatest reduction in cognitive biases (jumping to conclusions and bias against disconfirmatory evidence, small-to-medium effect sizes) following module 3. Counter-intuitively, increases in the severity of positive symptoms and cognitive biases were observed following the modules on self-esteem (module 9) and mood (module 8). The authors hypothesized that the content of those modules may be stressful for

participants due to their highly personal relevance, which could lead to emotional avoidance and an increase in positive symptoms.

Interestingly, a case report by Kumar et al.[25] described the effect of 12 sessions of individual MCT for a patient whose delusional symptoms had not responded to pharmacological treatment and electroconvulsive therapy. Following the intervention, the authors described improvements in positive and negative symptoms, general psychopathology, as well as a reduction on a scale measuring conviction of beliefs. Improvement in interpersonal relationships and social functioning were also reported. Another study by Briki et al.[26] graphically reported improvements in general and social functioning, as well.

Regarding the ideal target population for MCT, Moritz et al.[27] identified that patients presenting with low self-esteem, poor QoL and social anxiety/withdrawal might most benefit from the intervention. Similarly, Salas-Sender et al.[28] reported larger improvements for women in personalizing bias and irrational beliefs related to dependence following MCT. They also observed that men improved more on intolerance to frustration after the intervention. Men further presented with greater improvement on jumping to conclusions measures compared to women, but the condition (experimental or control) was not specified. The authors reported no difference between sexes on positive and negative symptoms at post-intervention and follow-up.

**eReferences**

1. Page MJ, McKenzie JE, Bossuyt PM, et al. The PRISMA 2020 statement: an updated guideline for reporting systematic reviews. *Bmj*. 2021;372
2. Moritz S, Woodward TS. Metacognitive training for schizophrenia patients (MCT): a pilot study on feasibility, treatment adherence, and subjective efficacy. *German Journal of Psychiatry*. 2007;10(3):69-78.
3. APA. *American Psychiatric Association, Diagnostic and statistical manual of mental disorders (DSM-5®)*. American Psychiatric Pub; 2013.
4. Trujols J, Portella MJ, Iraurgi I, Campins MJ, Siñol N, Cobos JPdL. Patient-reported outcome measures: are they patient-generated, patient-centred or patient-valued? *Journal of mental health*. 2013;22(6):555-562.
5. Ho B-C, Nopoulos P, Flaum M, Arndt S, Andreasen NC. Two-year outcome in first-episode schizophrenia: predictive value of symptoms for quality of life. *American Journal of Psychiatry*. 1998;155(9):1196-1201.
6. Rabinowitz J, Levine SZ, Garibaldi G, Bugarski-Kirola D, Berardo CG, Kapur S. Negative symptoms have greater impact on functioning than positive symptoms in schizophrenia: analysis of CATIE data. *Schizophrenia research*. 2012;137(1-3):147-150.
7. Hong QN, Pluye P, Fàbregues S, et al. Mixed methods appraisal tool (MMAT), version 2018. *Registration of copyright*. 2018;1148552:10.
8. Jauhar S, McKenna P, Radua J, Fung E, Salvador R, Laws K. Cognitive–behavioural therapy for the symptoms of schizophrenia: systematic review and meta-analysis with examination of potential bias. *The British Journal of Psychiatry*. 2014;204(1):20-29.
9. Eichner C, Berna F. Acceptance and efficacy of metacognitive training (MCT) on positive symptoms and delusions in patients with schizophrenia: a meta-analysis taking into account important moderators. *Schizophrenia bulletin*. 2016;42(4):952-962.
10. Shrier I, Boivin J-F, Steele RJ, et al. Should meta-analyses of interventions include observational studies in addition to randomized controlled trials? A critical examination of underlying principles. *American journal of epidemiology*. 2007;166(10):1203-1209.
11. Borenstein M, Hedges LV, Higgins JPT, Rothstein HR. *Introduction to Meta-Analysis*. John Wiley & Sons, Incorporated; 2011.
12. Efthimiou O, Mavridis D, Debray TP, et al. Combining randomized and non-randomized evidence in network meta-analysis. *Statistics in medicine*. 2017;36(8):1210-1226.
13. Cohen J. Statistical power analysis for the behavioral sciences. Lawrence Erlbaum Associates. *Hillsdale, NJ*. 1988:20-26.
14. Higgins J, Thomas J, Chandler J, et al. *Cochrane Handbook for Systematic Reviews of Interventions version 6.2*. Cochrane. www.training.cochrane.org/handbook; 2021.
15. Rosenthal R. Applied social research methods series, Vol. 6. Meta-analytic procedures for social research (Rev. ed.). Sage Publications, Inc. https://doi. org/10.4135/9781412984997; 1991.
16. Rosenthal R. The file drawer problem and tolerance for null results. *Psychological bulletin*. 1979;86(3):638.
17. Egger M, Smith GD, Schneider M, Minder C. Bias in meta-analysis detected by a simple, graphical test. *Bmj*. 1997;315(7109):629-634.
18. Borenstein M, Hedges LV, Higgins JP, Rothstein HR. *Introduction to meta-analysis*. John Wiley & Sons; 2021.

19. Cochran WG. The combination of estimates from different experiments. *Biometrics*. 1954;10(1):101-129.
20. Higgins JP, Thompson SG, Deeks JJ, Altman DG. Measuring inconsistency in meta-analyses. *Bmj*. 2003;327(7414):557-560.
21. Potvin S. La métaanalyse: Illustration pour déterminer si la toxicomanie aggrave les déficits cognitifs chez les personnes avec une schizophrénie. *M Corbière et N Larivière, Méthodes qualitatives, quantitatives et mixtes: Dans la recherche en sciences humaines, sociales et de la santé Presse de l'Université du Québec*. 2014;
22. Page MJ, Moher D, Bossuyt PM, et al. PRISMA 2020 explanation and elaboration: updated guidance and exemplars for reporting systematic reviews. *bmj*. 2021;372
23. Aghotor J, Pfueller U, Moritz S, Weisbrod M, Roesch-Ely D. Metacognitive training for patients with schizophrenia (MCT): feasibility and preliminary evidence for its efficacy. *Journal of behavior therapy and experimental psychiatry*. 2010;41(3):207-211.
24. Schneider BC, Cludius B, Lutz W, Moritz S, Rubel JA. An investigation of module-specific effects of metacognitive training for psychosis. *Zeitschrift für Psychologie*. 2018;
25. Kumar D, Rao MG, Raveendranathan D, Venkatasubramanian G, Varambally S, Gangadhar BN. Metacognitive training for delusion in treatment-resistant schizophrenia: A case report. *Clinical schizophrenia & related psychoses*. 2015;9(1):40-43.
26. Briki M, Vandel P, Haffen E, Sechter D. Metacognition training for schizophrenia: a French pilot study. *The Journal of neuropsychiatry and clinical neurosciences*. 2014;26(2):E32-E33.
27. Moritz S, Menon M, Andersen D, Woodward TS, Gallinat J. Moderators of symptomatic outcome in metacognitive training for psychosis (MCT). Who benefits and who does not? *Cognitive Therapy and Research*. 2018;42(1):80-91.
28. Salas-Sender M, López-Carrilero R, Barajas A, et al. Gender differences in response to metacognitive training in people with first-episode psychosis. *Journal of consulting and clinical psychology*. 2020;88(6):516.

**eAppendix 2. References of Studies Included in the Systematic Review and Meta-analysis**


1. Acuña, V., Otto, A., Cavieres, A., & Villalobos, H. (2021). Efficacy of Metacognitive Training in a Chilean Sample of People with Schizophrenia. *Revista Colombiana de Psiquiatria*.
2. Aghotor, J., Pfueller, U., Moritz, S., Weisbrod, M., & Roesch-Ely, D. (2010). Metacognitive training for patients with schizophrenia (MCT): feasibility and preliminary evidence for its efficacy. *Journal of behavior therapy and experimental psychiatry*, *41*(3), 207-211.
3. Andreou, C., Wittekind, C. E., Fieker, M., Heitz, U., Veckenstedt, R., Bohn, F., & Moritz, S. (2017). Individualized metacognitive therapy for delusions: a randomized controlled rater-blind study. *Journal of behavior therapy and experimental psychiatry*, *56*, 144-151.
4. Andreou, C., Steinmann, S., Leicht, G., Kolbeck, K., Moritz, S., & Mulert, C. (2018). fMRI correlates of jumping-to-conclusions in patients with delusions: Connectivity patterns and effects of metacognitive training. *NeuroImage: Clinical*, *20*, 119-127.
5. Balzan, R. P., Delfabbro, P. H., Galletly, C. A., & Woodward, T. S. (2014). Metacognitive training for patients with schizophrenia: preliminary evidence for a targeted, single-module programme. *Australian & New Zealand Journal of Psychiatry*, *48*(12), 1126-1136.
6. Balzan, R. P., Mattiske, J. K., Delfabbro, P., Liu, D., & Galletly, C. (2019). Individualized metacognitive training (MCT+) reduces delusional symptoms in psychosis: A randomized clinical trial. *Schizophrenia bulletin*, *45*(1), 27-36.
7. Briki, M., Vandel, P., Haffen, E., & Sechter, D. (2014a). Metacognition training for schizophrenia: a French pilot study. *The Journal of neuropsychiatry and clinical neurosciences*, *26*(2), E32-E33.
8. Briki, M., Monnin, J., Haffen, E., Sechter, D., Favrod, J., Netillard, C., Cheraitia, E., Marin, K., Govyadovskaya, S., & Tio, G. (2014b). Metacognitive training for schizophrenia: a multicentre randomised controlled trial. *Schizophrenia research*, *157*(1-3), 99-106.
9. Chen, Q., Sang, Y., Ren, L., Wu, J., Chen, Y., Zheng, M., Bian, G., & Sun, H. (2021). Metacognitive training: a useful complement to community-based rehabilitation for schizophrenia patients in China. *BMC psychiatry*, *21*(1), 1-10.
10. de Pinho, L. M. G., Sequeira, C. A. d. C., Sampaio, F. M. C., Rocha, N. B., Ozaslan, Z., & Ferre-Grau, C. (2021). Assessing the efficacy and feasibility of providing metacognitive training for patients with schizophrenia by mental health nurses: A randomized controlled trial. *Journal of Advanced Nursing*, *77*(2), 999-1012.
11. Erawati, E., Keliat, B. A., Helena, N., & Hamid, A. (2014). The influence of metacognitive training on delusion severity and metacognitive ability in schizophrenia. *Journal of psychiatric and mental health nursing*, *21*(9), 841-847.
12. Favrod, J., Maire, A., Bardy, S., Pernier, S., & Bonsack, C. (2011). Improving insight into delusions: a pilot study of metacognitive training for patients with schizophrenia. *Journal of advanced nursing*, *67*(2), 401-407.
13. Favrod, J., Rexhaj, S., Bardy, S., Ferrari, P., Hayoz, C., Moritz, S., Conus, P., & Bonsack, C. (2014). Sustained antipsychotic effect of metacognitive training in psychosis: A randomized-controlled study. *European Psychiatry*, *29*(5), 275-281.
14. Fekete , Z., Vass, E., Balajthy , R., Tana , Ü., Nagy , A. C., Oláh , B., Domján , N., & Kuritárné, I. S. (unpublished) *Efficacy of Metacognitive Training on symptom severity,*

*neurocognition and social cognition in patients with schizophrenia: a single-blind, randomised, controlled trial*.

15. Ferwerda, J., de Boer, K., & van der Gaag, M. (2010). Metacognitieve training voor patiënten met een psychotische kwetsbaarheid. *Directieve therapie*, *30*(4), 263-279.
16. Fujii, K., Kobayashi, M., Funasaka, K., Kurokawa, S., & Hamagami, K. (2021). Effectiveness of Metacognitive Training for Long-Term Hospitalized Patients with Schizophrenia: A Pilot Study with a Crossover Design. *Asian Journal of Occupational Therapy*, *17*(1), 45-52.
17. Gawęda, Ł., Krężołek, M., Olbryś, J., Turska, A., & Kokoszka, A. (2015). Decreasing self-reported cognitive biases and increasing clinical insight through meta-cognitive training in patients with chronic schizophrenia. *Journal of behavior therapy and experimental psychiatry*, *48*, 98-104.
18. Ishikawa, R., Ishigaki, T., Shimada, T., Tanoue, H., Yoshinaga, N., Oribe, N., Morimoto, T., Matsumoto, T., & Hosono, M. (2020). The efficacy of extended metacognitive training for psychosis: A randomized controlled trial. *Schizophrenia research*, *215*, 399-407.
19. Kowalski, J., Pankowski, D., Lew-Starowicz, M., & Gawęda, Ł. (2017). Do specific metacognitive training modules lead to specific cognitive changes among patients diagnosed with schizophrenia? A single module effectiveness pilot study. *Psychosis*, *9*(3), 254-259.
20. Kumar, D., Zia Ul Haq, M., Dubey, I., Dotivala, K. N., Veqar Siddiqui, S., Prakash, R., Abhishek, P., & Nizamie, S. H. (2010). Effect of meta-cognitive training in the reduction of positive symptoms in schizophrenia. *European Journal of Psychotherapy and Counselling*, *12*(2), 149-158.
21. Kumar, D., Rao, M. G., Raveendranathan, D., Venkatasubramanian, G., Varambally, S., & Gangadhar, B. N. (2015). Metacognitive training for delusion in treatment-resistant schizophrenia: A case report. *Clinical schizophrenia & related psychoses*, *9*(1), 40-43.
22. Kuokkanen, R., Lappalainen, R., Repo-Tiihonen, E., & Tiihonen, J. (2014). Metacognitive group training for forensic and dangerous non-forensic patients with schizophrenia: A randomised controlled feasibility trial. *Criminal Behaviour and Mental Health*, *24*(5), 345-357.
23. Kuokkanen, R., Aho-Mustonen, K., Muotka, J., Lappalainen, R., & Tiihonen, J. (2015). A pilot study of group administered metacognitive training (MCT) for schizophrenia patients in a high-security forensic setting: Subjective training success and health-related quality of life. *Journal of Forensic Psychology Practice*, *15*(4), 344-362.
24. Lopez-Morinigo, J.-D., Sánchez-Escribano Martínez, A., Barrigón, M. L., Escobedo-Aedo, P.-J., González Ruiz-Ruano, V., Sánchez-Alonso, S., Mata-Iturralde, L., Muñoz-Lorenzo, L., Cuadras, D., Ochoa, S., Baca-García, E., & David, A. S. (unpublished). *Randomised controlled trial comparing metacognitive training to psychoeducation in schizophrenia: Effects on insight and outcomes over a 1-year follow-up*.
25. Moritz, S., Kerstan, A., Veckenstedt, R., Randjbar, S., Vitzthum, F., Schmidt, C., Heise, M., & Woodward, T. (2011a). Further evidence for the efficacy of a metacognitive group training in schizophrenia. *Behaviour research and therapy*, *49*(3), 151-157.
26. Moritz, S., Veckenstedt, R., Randjbar, S., Vitzthum, F., & Woodward, T. (2011b). Antipsychotic treatment beyond antipsychotics: metacognitive intervention for schizophrenia patients improves delusional symptoms. *Psychological medicine*, *41*(9), 1823-1832.

27. Moritz, S., Veckenstedt, R., Bohn, F., Hottenrott, B., Scheu, F., Randjbar, S., Aghotor, J., Köther, U., Woodward, T. S., & Treszl, A. (2013). Complementary group Metacognitive Training (MCT) reduces delusional ideation in schizophrenia. *Schizophrenia research, 151*(1-3), 61-69.
28. Moritz, S., Veckenstedt, R., Andreou, C., Bohn, F., Hottenrott, B., Leighton, L., Köther, U., Woodward, T. S., Treszl, A., & Menon, M. (2014). Sustained and “sleeper” effects of group metacognitive training for schizophrenia: a randomized clinical trial. *JAMA psychiatry, 71*(10), 1103-1111.
29. Moritz, S., Menon, M., Andersen, D., Woodward, T. S., & Gallinat, J. (2018). Moderators of symptomatic outcome in metacognitive training for psychosis (MCT). Who benefits and who does not? *Cognitive Therapy and Research, 42*(1), 80-91.
30. Naughton, M., Nulty, A., Abidin, Z., Davoren, M., O’Dwyer, S., & Kennedy, H. G. (2012). Effects of group metacognitive training (MCT) on mental capacity and functioning in patients with psychosis in a secure forensic psychiatric hospital: a prospective-cohort waiting list controlled study. *BMC research notes, 5*(1), 1-11.
31. Ochoa, S., López-Carrilero, R., Barrigón, M., Pousa, E., Barajas, A., Lorente-Rovira, E., González-Higueras, F., Grasa, E., Ruiz-Delgado, I., & Cid, J. (2017). Randomized control trial to assess the efficacy of metacognitive training compared with a psycho-educational group in people with a recent-onset psychosis. *Psychological Medicine, 47*(9), 1573-1584.
32. Ochoa, S., Lopez-Carrilero, R., Barrigon, M. L., Pousa, E., Grasa, E., González, F., & Monserrat, C. (2020). S34. Effectiveness of individual metacognitive training (MCT+) in first-episode psychosis. *Schizophrenia Bulletin, 46*(Suppl 1), S44.
33. Park, S., Lee, H. K., & Kim, H. (2020). Effects of a Korean version of the metacognitive training program for outpatients with schizophrenia on theory of mind, positive symptoms, and interpersonal relationships. *Behavioural and cognitive psychotherapy, 48*(1), 14-24.
34. Pos, K., Meijer, C. J., Verkerk, O., Ackema, O., Krabbendam, L., & de Haan, L. (2018). Metacognitive training in patients recovering from a first psychosis: an experience sampling study testing treatment effects. *European archives of psychiatry and clinical neuroscience, 268*(1), 57-64.
35. Raucher-Chéné, D. M., Daniel, Thibaudeau, É., & Lepage, M. (unpublished). *Virtual metacognitive training for psychosis: Preliminary findings from a Canadian cohort study*.
36. Salas-Sender, M., López-Carrilero, R., Barajas, A., Lorente-Rovira, E., Pousa, E., Barrigón, M. L., Grasa, E., Ruiz-Delgado, I., González-Higueras, F., & Cid, J. (2020). Gender differences in response to metacognitive training in people with first-episode psychosis. *Journal of consulting and clinical psychology, 88*(6), 516.
37. Schneider, B. C., Cludius, B., Lutz, W., Moritz, S., & Rubel, J. A. (2018). An investigation of module-specific effects of metacognitive training for psychosis. *Zeitschrift für Psychologie*.
38. Shan, X., Liao, R., Ou, Y., Pan, P., Ding, Y., Liu, F., Chen, J., Zhao, J., Guo, W., & He, Y. (2021). Increased regional homogeneity modulated by metacognitive training predicts therapeutic efficacy in patients with schizophrenia. *European archives of psychiatry and clinical neuroscience, 271*(4), 783-798.

39. Simón-Expósito, M., & Felipe-Castaño, E. (2019). Effects of metacognitive training on cognitive insight in a sample of patients with schizophrenia. *International journal of environmental research and public health*, *16*(22), 4541.
40. So, S. H.-W., Chan, A. P., Chong, C. S.-Y., Wong, M. H.-M., Lo, W. T.-L., Chung, D. W.-S., & Chan, S. S. (2015). Metacognitive training for delusions (MCTd): effectiveness on data-gathering and belief flexibility in a Chinese sample. *Frontiers in Psychology*, *6*, 730.
41. So, S. H.-w., Chan, G. H.-k., Wong, C. K.-w., Ching, E. W.-k., Lee, S. S.-w., Wong, B. C.-W., Zhu, C., Sun, X., Chung, L. K.-h., & Hung, A. Y. (2021). A randomised controlled trial of metacognitive training for psychosis, depression, and belief flexibility. *Journal of Affective Disorders*, *279*, 388-397.
42. Tanoue, H., Yoshinaga, N., Hayashi, Y., Ishikawa, R., Ishigaki, T., & Ishida, Y. (2021). Clinical effectiveness of metacognitive training as a transdiagnostic program in routine clinical settings: A prospective, multicenter, single-group study. *Japan Journal of Nursing Science*, *18*(2), e12389.
43. Ussorio, D., Giusti, L., Wittekind, C. E., Bianchini, V., Malavolta, M., Pollice, R., Casacchia, M., & Roncone, R. (2016). Metacognitive training for young subjects (MCT young version) in the early stages of psychosis: Is the duration of untreated psychosis a limiting factor? *Psychology and Psychotherapy: Theory, Research and Practice*, *89*(1), 50-65.
44. van Oosterhout, B., Krabbendam, L., De Boer, K., Ferwerda, J., Van der Helm, M., Stant, A., & Van der Gaag, M. (2014). Metacognitive group training for schizophrenia spectrum patients with delusions: a randomized controlled trial. *Psychological medicine*, *44*(14), 3025-3035.
45. Yildiz, M., Özaslan, Z., Incedere, A., Kircali, A., Kiras, F., & İpçi, K. (2019). The effect of psychosocial skills training and metacognitive training on social and cognitive functioning in schizophrenia. *Archives of Neuropsychiatry*, *56*(2), 139.
46. Zalzala, A., Wardwell, P., Petrik, T., Mathews, L., Moritz, S., Fiszdon, J., Haber, L. C., Shagan, D., Bracken, D., & Choi, J. (2019). F121. Metacognitive training (MCT) to improve insight and work outcome in schizophrenia. *Schizophrenia Bulletin*, *45*(Suppl 2), S299-S300.

**eTable 1. Systematic review search strategy.**

| Database | Search |
|---|---|
| CENTRAL | ID Search Hits<br>#1 MeSH descriptor: [Schizophrenia Spectrum and Other Psychotic Disorders] explode all trees 9347<br>#2 (schizo* or delusion* or psychosis or psychoses or psychotic* or first episode* or first-episode* or fep*) 37204<br>#3 ("metacognitive" NEXT train*) 138<br>#4 ("meta-cognitive" NEXT train*) 19<br>#5 MCT 1240<br>#6 #1 or #2 37214<br>#7 #3 or #4 or #5 1284<br>#8 #6 and #7 122<br>Results: 122 |
| CINAHL | (schizo* or delusion* or psychosis or psychoses or psychotic* or first episode* or first-episode* or fep*) TX All Text<br>AND<br>(metacognitive train* or meta-cognitive train*) TI Title<br>OR<br>(metacognitive train* or meta-cognitive train*) AB Abstract<br>OR<br>(metacognitive train* or meta-cognitive train*) TX All Text<br>Results: 87 |
| PubMed | (("Schizophrenia Spectrum and Other Psychotic Disorders"[Mesh])<br>OR<br>(schizo* or delusion* or psychosis or psychoses or psychotic* or first episode* or first-episode* or fep))<br>AND<br>((("metacognitive" train*) OR ("meta-cognitive" train*) OR (MCT))<br>AND<br>("2007"[Date - Publication] : "3000"[Date - Publication]))<br>Results: 160 |
| Embase | 1 exp psychosis/ or exp acute psychosis/ or exp affective psychosis/ or exp brief psychotic disorder/ or exp childhood psychosis/ or exp delusion/ or exp depressive psychosis/ or exp endogenous psychosis/ or exp hallucination/ or exp intensive care psychosis/ or exp manic psychosis/ or exp paranoid psychosis/ or exp puerperal psychosis/ or exp schizophrenia/<br>2 (schizo* or delusion* or psychos* or psychotic* or first episode* or first-episode* or fep*).mp.<br>3 (metacognitive train* or meta-cognitive train*).mp.<br>4 MCT.mp.<br>5 1 or 2<br>6 4 and 5<br>7 3 or 6<br>Results: 191 |
| MEDLINE | exp "schizophrenia spectrum and other psychotic disorders"/<br>(schizo* or delusion* or psychos* or psychotic* or first episode* or first-episode* or fep*).tw,kf.<br>(("metacognitive" adj train*) or ("meta-cognitive" adj train*)).mp.<br>MCT.tw,kf.<br>1 or 2<br>3 or 4<br>5 and 6<br>limit 7 to yr="2007 -Current" |

| Database | Search |
|---|---|
| MEDLINE *(continued)* | Results: 108 |
| PsycINFO | 1 psychosis/ or exp acute psychosis/ or exp affective psychosis/ or exp childhood psychosis/ or exp chronic psychosis/ or exp "paranoia (psychosis)"/ or exp schizophrenia/ or exp paranoid schizophrenia/<br>2 (schizo* or delusion* or psychos* or psychotic* or first episode* or first-episode* or fep*).mp.<br>3 (metacognitive train* or meta-cognitive train*).mp.<br>4 MCT.mp.<br>5 1 or 2<br>6 4 and 5<br>7 3 or 6<br>Results: 129 |
| Social Work Abstracts | 1 (schizo* or delusion* or psychos* or psychotic* or first episode* or first-episode* or fep*).mp.<br>2 (metacognitive train* or meta-cognitive train*).mp.<br>3 MCT.mp.<br>4 1 and 3<br>5 2 or 4<br>6 limit 5 to yr="2007 -Current"<br>Results: 0 |
| Web of Science | ALL=(schizo* or delusion* OR psychosis OR psychoses OR psychotic* OR first episode* OR first-episode* OR fep*) AND TS=(("metacognitive" NEAR train*) OR ("meta-cognitive" NEAR train*) OR (MCT) ) AND PY=(2007-2021)<br>TOPIC: (schizophrenia spectrum disorders) OR ALL FIELDS:<br>((schizo* or delusion* OR psychosis OR psychoses OR psychotic* OR first episode* OR first-episode* OR fep*) ) AND ALL FIELDS:<br>((metacognitive train* OR meta-cognitive train* OR MCT) ) AND YEAR PUBLISHED:<br>(2007-2021)<br>Results: 188 |
| Open Grey | (schizo* OR delusion* OR psychosis OR psychoses OR psychotic* OR first episode* OR first-episode* OR fep*) AND (("meta-cognitive" NEAR train*)OR("metacognitive" NEAR train*) OR(MCT))<br>Results: 1 |
| ProQuest Dissertations | (schizo* OR delusion* OR psychosis OR psychoses OR psychotic* OR first episode* OR first-episode* OR fep*) AND (("meta-cognitive training")OR("metacognitive training")) YR(>2007)<br>Results: 59 |
| Social Science Research Network | SSRN search terms (Medical research network; Psychology research network)<br><br>(schizophrenia OR delusion OR psychosis OR psychoses OR psychotic OR first episode psychosis OR first-episode psychosis OR fep) AND ((meta-cognitive training) OR (metacognitive training))<br>Results: 0 |

*Note.* The search was conducted on June 3, 2021, by author DM and assisted by author DP; total search results = 1045; duplicates = 526; total studies screened = 519.

**eTable 2. Core modules of metacognitive training (MCT) for psychosis**.

| Module | Target Domain(s) | Core Exercises |
|---|---|---|
| Attribution: Blaming and Taking Credit | Self-serving bias *vs* depressive attributional style | - Different causes of positive and negative events are contemplated<br>- Explanations taking into account various causes are preferred to monocausal explanations<br>- The negative consequences of self-serving attribution are repeatedly highlighted |
| Jumping to Conclusions: I | Jumping to conclusions/ liberal acceptance/ bias against disconfirmatory evidence | Part I<br>- Motifs contributing to hasty decision making are discussed; disadvantages are stressed<br>- Fragmented pictures are shown that eventually reveal objects; premature decisions often lead to errors<br>- Emphasis on the benefits of cautious data gathering<br>Part II<br>- Ambiguous pictures are displayed often leading to omission of details<br>- Demonstrate that first impressions often reveal only half truths |
| Changing Beliefs | Bias against disconfirmatory evidence | - Cartoon sequences are shown in backwards order, increasingly disambiguating a complex scenario<br>- After each (new) picture is revealed, patients are asked to (re-)rate the plausibility of four interpretations<br>- In some (but not all) pictures, the initial sequence is most likely interpretation prevails<br>- Exercise encourages learning to withhold strong judgments until sufficient evidence has been collected<br>- Encourages maintenance of an open attitude toward counterarguments and alternative views |
| To Empathize: I | First order theory of mind | Part I<br>- Facial expression and other cues are discussed for their relevance to social reasoning.<br>- Pictures of human faces are presented; group members guess what the depicted character(s) may be feeling<br>- The correct answer often violates intuition, demonstrating that relying on facial expression alone can be misleading<br>Part II<br>- Cartoon strips must be completed or reorganized to display the correct order<br>- Highlights how social inferences should involve multiple cues |
| Memory | Overconfidence in errors | - Discuss factors that foster or impair memory acquisition<br>- Examples of common false memories are presented<br>- Complex scenes are displayed with two typical elements each removed |

| Module | Target Domain(s) | Core Exercises |
|---|---|---|
| Memory *(continued)* | Overconfidence in errors | - Reliance on logical inference, gist-based recollection and liberal acceptance, many individuals falsely recognize lure items in a later recognition trial<br>- Highlighting the constructive rather than passive nature of memory<br>- Teach differentiation between false and correct memories using the vividness heuristic |
| To empathize: II | Second order theory of mind/ need for closure | - Discuss various aspects guiding theory of mind, with respect to both their heuristic value and fallibility for social decision making<br>-Cartoon sequences are presented, observers must consider the perspective of the protagonist, which involves discounting knowledge available to the observer but not available to the protagonist<br>- No definitive solutions can be inferred in the majority of instances, which is unsatisfactory for those with an enhanced need for closure |
| Jumping to Conclusions: II | Jumping to conclusions/ liberal acceptance | - The disadvantages of quick decision making are outlined with respect to events related and unrelated to psychosis<br>- Paintings are displayed; the correct title must be deduced from four response options<br>- Upon superficial inspection, many paintings tempt false responses |
| Mood | Mood | - Depressive symptoms, causes, and treatment options are discussed<br>- Typical depressive cognitive patterns in response to common events are presented (e.g. over-generalization, selective abstraction)<br>- Patient(s) is asked to come up with more constructive and positive responses<br>- Convey strategies to help transform negative self-schemata and promote mood elevation |
| Self-Esteem | Self-esteem | - Define and discuss how low self-esteem develops<br>- Explain the differences between low, exaggerated, and healthy self-esteem<br>- Emphasize that knowledge and appreciation of one's strengths are the basis of healthy self-esteem<br>- Identify that there are several sources of self-esteem<br>-Facilitators set a healthy example by naming their own strengths; patients are encouraged to do the same<br>-Address relationship between behavior (slouching) and mood; how good posture can enhance self-esteem<br>- Strategies and suggestions that can be implemented in everyday life to help increase self-esteem |
| Stigma | Stigma | - Global aim is to challenge prejudice and to reduce self-stigmatization |

| Module | Target Domain(s) | Core Exercises |
| --- | --- | --- |
| Stigma *(continued)* | Stigma | -Normalization of mental illness with emphasis on how not even famous people are spared from mental health problems<br>- Highlight how people with psychological problems can also make meaningful and valuable contributions<br>- Illustrate the high prevalence of mental disorders in the general population and convey that attenuated symptoms of psychosis are quite common<br>- Define self-stigmatization, discuss its roots, and convey how stigmatization can harm self-esteem and self-image<br>- Discussion on how to deal with prejudice, and how to self-advocate when feeling stigmatized<br>- Participants will be given strategies on when and how to talk with others about their illness |

*Note.* Summaries of the metacognitive training modules were obtained by the developers, S. Moritz and T. Woodward. In the original 8-module version, mood and self-esteem comprise module 8, and there is no module targeting stigma.

**eTable 3. Comprehensive list of extracted variables.**

| Proximal Outcome | Extracted Rating Scale |
|---|---|
| Positive Symptoms | Brief Psychiatric Rating Scale; BPRS Scale Score<br>Clinical Global Impression-Severity; CGI-S<br>Green Paranoid Thought Scale; GPTS<br>Questionnaire developed by author: Positive Symptoms<br>Positive and Negative Syndrome Scale; PANSS Scale Score<br>Psychotic Symptom Rating Scales; PSYRATS<br>Scale for the Assessment of Positive Symptoms; SAPS |
| Delusions | Brown's Assessment of Beliefs Scale; BABS<br>Delusion Rating Scale; DRS<br>Experience Sampling Measure - Paranoid ideation<br>Experience Sampling Measure - Delusional conviction<br>GPTS<br>Paranoia Checklist<br>Peters et al. Delusion Inventory; PDI<br>PSYRATS-Delusions<br>PANSS Factor Score |
| Hallucinations | PANSS Factor Score<br>PSYRATS-Hallucinations |
| Cognitive Biases | Bias Against Disconfirmatory Evidence (BADE)<br>Beads Task<br>Beads Task Variants (e.g., Fish Task)<br>Box Task<br>Cognitive Biases Questionnaire for Psychosis; CBQp Scale or Factor Score<br>Davos Assessment of Cognitive Biases Scales; DACOBS<br>Illusion of Control Task<br>Internal, Personal, and Situational Attributions Questionnaire; IPSAQ<br>Jumping to conclusion Fish/Lakes Task Confidence<br>Maudsley Assessment of Delusions Scale; MADS Scale or Factor Score<br>Probabilistic Reasoning Task<br>Questionnaire developed by author: Jumping to Conclusions<br>Representativeness Task |
| **Distal Outcome** | |
| Functioning | Global Assessment of Functioning; GAF (global functioning)<br>Life Skills Profile (general functioning)<br>Mini-ICF-APP (occupational and social functioning)<br>Personal and Social Performance Scale; PSP (socially useful activities, personal and social relationships, self-care, disturbing and aggressive behaviours)<br>Quality of Life Scale; QLS (Interpersonal Relations subscales, assesses social functioning in schizophrenia)<br>Relationship Change Scale (social functioning, relationship satisfaction)<br>Social Network Questionnaire; SNQ (social functioning)<br>World Health Organization Disability Assessment Schedule 2; WHODAS 2.0 (measures global functioning via assessment of cognition, mobility, self-care, getting along, life activities (household and work/school) and participation) |
| Negative Symptoms | BPRS Scale Score<br>PANSS Scale Score<br>Scale for the Assessment of Negative Symptoms; SANS |

| Distal Outcome | Extracted Rating Scale |
|---|---|
| Quality of Life *(continued)* | 15D<br>EuroQoL-5D; EQ-5D<br>Quality of Life Enjoyment and Satisfaction Questionnaire; Q-LES-Q 18<br>Quality of Life Scale; QLS<br>Satisfaction Life Domains Scale<br>Schizophrenia Quality of Life Scale; SQLS<br>World Health Organization Disability Schedule<br>World Health Organization Quality of life scale; WHOQOL; WHOQOL-BREF |
| Self-Esteem | Rosenberg Self-Esteem Scale; RSES<br>Self-Esteem Rating Scale; SERS<br>Self-esteem rating scale-short form; SERS-SF |

## eTable 4. Quality assessment rating of included studies using the Mixed Methods Appraisal Tool (MMAT).

| Study Frist Author(s) | Year | Screening Questions | | Randomized Controlled Trials | | | | | MMAT Score | Comments |
|---|---|---|---|---|---|---|---|---|---|---|
| | | S1. Are there clear research questions? | S2. Do the collected data allow to address the research questions? | 1. Is randomization appropriately performed? | 2. Are the groups comparable at baseline? | 3. Are there complete outcome data? | 4. Are outcome assessors blinded to the intervention provided? | 5 Did the participants adhere to the assigned intervention? | | |
| Acuna | 2021 | yes | yes | no | no | yes | yes | yes | 3 | |
| Aghotor | 2010 | yes | yes | no | yes | no | yes | yes | 3 | MCT had complete outcome data, control group did not; dropouts associated with discharge |
| Andreou | 2017 | yes | yes | yes | no | no | yes | no | 2 | ITT analyses |
| Balzan | 2019 | yes | yes | yes | no | yes | yes | yes | 4 | |
| Briki | 2014 b | yes | yes | no | yes | no | yes | no | 2 | |
| Chen | 2021 | yes | yes | no | yes | yes | yes | yes | 4 | |
| de Pinho | 2020 | yes | yes | yes | yes | yes | yes | yes | 5 | ITT analyses; manuscript does not report dropouts who completed pre but not post and/or follow up |
| Favrod | 2014 | yes | yes | no | yes | yes | yes | yes | 4 | |
| Fekete | UD | yes | yes | yes | yes | no | yes | yes | 4 | Incomplete outcome data is due to dropout from the control group; ITT analyses |
| Fujii | 2017 | yes | yes | yes | yes | no | no | no | 2 | |
| Gaweda | 2015 | yes | yes | yes | yes | yes | no | yes | 4 | |
| Ishikawa | 2020 | yes | yes | yes | yes | yes | yes | yes | 5 | ITT analyses |
| Kowalski | 2017 | yes | yes | no | no | no | no | yes | 1 | |
| Kumar | 2010 | yes | yes | yes | yes | no | no | no | 2 | |

| Study Frist Author(s) | Year | Screening Questions | | Randomized Controlled Trials | | | | | MMAT Score | Comments |
|---|---|---|---|---|---|---|---|---|---|---|
| | | S1. Are there clear research questions? | S2. Do the collected data allow to address the research questions? | 1. Is randomization appropriately performed? | 2. Are the groups comparable at baseline? | 3. Are there complete outcome data? | 4. Are outcome assessors blinded to the intervention provided? | 5 Did the participants adhere to the assigned intervention? | | |
| Kuokkanen<br>Kuokkanen | 2014<br>2015 | yes | yes | yes | yes | yes | yes | yes | 5 | |
| Lopez-Morinigo | UD | yes | yes | yes | yes | no | yes | no | 3 | |
| Moritz | 2011 a | yes | yes | yes | yes | yes | yes | yes | 5 | |
| Moritz | 2011 b | yes | yes | yes | yes | yes | yes | yes | 5 | ITT analyses |
| Moritz<br>Moritz<br>Moritz | 2013<br>2014<br>2018 | yes | yes | no | yes | yes | yes | yes | 4 | ITT analyses; Moritz (2018) not included in quantitative analyses |
| Ochoa<br>Salas-Sender | 2017<br>2020 | yes | yes | yes | yes | no | yes | no | 3 | Salas-Sender (2020) not included in quantitative analyses |
| Ochoa | 2020 | yes | yes | no | no | no | yes | no | 3 | Rating based on a conference abstract + supplemental info provided by the author |
| Park | 2020 | yes | yes | no | yes | no | no | yes | 2 | ITT analyses; post treatment data not reported for all measures |
| Pos | 2018 | yes | yes | no | no | no | yes | yes | 2 | |
| Shan | 2021 | yes | yes | yes | yes | yes | yes | yes | 5 | |
| So | 2015 | yes | yes | no | yes | no | yes | no | 2 | ITT analyses |
| So | 2021 | yes | yes | no | yes | no | yes | no | 2 | ITT analyses |
| van Oosterhout | 2014 | yes | yes | yes | yes | no | yes | yes | 4 | ITT analyses |

| Study Frist Author(s) | Year | Screening Questions | | Randomized Controlled Trials | | | | | MMAT Score | Comments |
|---|---|---|---|---|---|---|---|---|---|---|
| | | S1. Are there clear research questions? | S2. Do the collected data allow to address the research questions? | 1. Is randomization appropriately performed? | 2. Are the groups comparable at baseline? | 3. Are there complete outcome data? | 4. Are outcome assessors blinded to the intervention provided? | 5 Did the participants adhere to the assigned intervention? | | |
| Yildiz | 2019 | yes | yes | no | yes | yes | yes | yes | 4 | |
| Zalzala | 2019 | yes | yes | no | yes | yes | no | yes | 3 | Rating based on a conference abstract + supplemental table provided by the author |

*Note.* Ratings were randomly allocated to two independent reviewers (DP and DM); interrater agreement was 85. 71%, established for 10% of studies randomly allocated to both reviewers; discrepancies were resolved via consensus; all study designs are based on 5 methodological quality criteria, where the highest possible score is 5 (indicating the highest quality) and the lowest possible score is 0 (indicating the lowest quality; possible responses on all items include “yes” = criterium met; “no” = criterium not met; “can’t tell” = inadequate information to assess a given criterium; incomplete outcome data was qualified by attrition rates exceeding 20% in at least one study group (i.e., treatment or control); UD = unpublished data; ITT = intention-to-treat; a = unpublished data from our group, thus an independent reviewer (A.E. de Sousa) from our group but with no conflict of interest (neither in regard to the unpublished data nor the current systematic review and meta-analysis) rated MMAT criteria; columns assessing qualitive and mixed method studies were not included in the table given no such studies were identified in our search; for more information: http://mixedmethodsappraisaltoolpublic.pbworks.com/.

| Study First Author(s) | Year | Screening Questions | | Non-randomized Controlled Trials | | | | | MMAT Score | Comments |
|---|---|---|---|---|---|---|---|---|---|---|
| | | **S1. Are there clear research questions?** | **S2. Do the collected data allow to address the research questions?** | **1. Are the participants representative of the target population?** | **2. Are measurements appropriate regarding both the outcome and intervention (or exposure)?** | **3. Are there complete outcome data?** | **4. Are the confounders accounted for in the design and analysis?** | **5. During the study period, is the intervention administered (or exposure occurred) as intended?** | | |
| Andreou | 2018 | yes | yes | no | yes | yes | yes | yes | 4 | |
| Balzan | 2014 | yes | yes | yes | yes | yes | yes | yes | 5 | |
| Erawati | 2014 | yes | yes | yes | yes | yes | no | yes | 4 | |
| Favrod | 2011 | yes | yes | yes | yes | no | no | yes | 3 | |
| Ferwerda | 2010 | yes | yes | can't tell | yes | can't tell | no | can't tell | 1 | |
| Naughton | 2012 | yes | yes | can't tell | yes | yes | yes | yes | 4 | |
| Raucher-Chene | UD | yes | yes | yes | yes | no | no | yes | 3 | Completion rate was variable across outcomes ranging from 53.85% - 69.23% for all outcomes |
| Schneider | 2018 | yes | yes | can't tell | no | can't tell | no | yes | 1 | |
| Simon-Exposito | 2019 | yes | yes | yes | yes | yes | yes | yes | 5 | |
| Tanoue | 2021 | yes | yes | yes | yes | no | no | can't tell | 2 | ITT analyses |
| Ussorio | 2016 | yes | yes | yes | yes | yes | yes | yes | 5 | |

*Note.* Ratings were randomly allocated to two independent reviewers (DP and DM); interrater agreement was 85. 71%, established for 10% of studies randomly allocated to both reviewers; discrepancies were resolved via consensus; all study designs are based on 5 methodological quality criteria, where the highest possible score is 5 (indicating the highest quality) and the lowest possible score is 0 (indicating the lowest quality; possible responses on all items include "yes" = criterium met; "no" = criterium not met; "can't tell" = inadequate information to assess a given criterium; incomplete outcome data was qualified by attrition rates exceeding 20% in at least one study group (i.e., treatment or control); UD = unpublished data; ITT = intention-to-treat; a = unpublished data from our group, thus an independent reviewer (A.E. de Sousa) from our group but with no conflict of interest (neither in regard to the unpublished data nor the current systematic review and meta-analysis) rated MMAT criteria; columns assessing qualitive and mixed method studies were not included in the table given no such studies were identified in our search; for more information: http://mixedmethodsappraisaltoolpublic.pbworks.com/.

| Study First Author(s) | Year | Screening Questions | | Quantitative Descriptive Studies | | | | | | |
|---|---|---|---|---|---|---|---|---|---|---|
| | | S1. Are there clear research questions? | S2. Do the collected data allow to address the research questions? | 1. Is the sampling strategy relevant to address the research question? | 2. Is the sample representative of the target population? | 3. Are the measurements appropriate? | 4. Is the risk of nonresponse bias low? | 5. Is the statistical analysis appropriate to answer the research question? | | |
| Briki | 2014 a | yes | yes | yes | yes | yes | yes | yes | 5 | |
| Kumar | 2015 | no | can't tell | can't tell | yes | yes | yes | N/A | 3 | Case report; data were not analyzed |

*Note.* Ratings were randomly allocated to two independent reviewers (DP and DM); interrater agreement was 85. 71%, established for 10% of studies randomly allocated to both reviewers; discrepancies were resolved via consensus; all study designs are based on 5 methodological quality criteria, where the highest possible score is 5 (indicating the highest quality) and the lowest possible score is 0 (indicating the lowest quality; possible responses on all items include “yes” = criterium met; “no” = criterium not met; “can’t tell” = inadequate information to assess a given criterium; incomplete outcome data was qualified by attrition rates exceeding 20% in at least one study group (i.e., treatment or control); UD = unpublished data; ITT = intention-to-treat; a = unpublished data from our group, thus an independent reviewer (A.E. de Sousa) from our group but with no conflict of interest (neither in regard to the unpublished data nor the current systematic review and meta-analysis) rated MMAT criteria; columns assessing qualitive and mixed method studies were not included in the table given no such studies were identified in our search; for more information: http://mixedmethodsappraisaltoolpublic.pbworks.com/.

**eTable 5. Supplemental description of included studies.**

| First Author(s) | Year | Diagnosis | Age Range | % N Antipsy | Dose EQ (mean) | Dose EQ (SD) | Outcome (measure) | FUP |
|---|---|---|---|---|---|---|---|---|
| Acuna | 2021 | SSD | ExpGr (18-45); CtrlGr (19-36) | 100% | ExpGr (653.4); CtrlGr (542.85) | ExpGr (363.68); CtrlGr (282.30) | Positive sx (PANSS positive); Negative sx (PANSS negative); Cognitive bias (CBQp jumping to conclusions, total) | N/A |
| Aghotor | 2010 | SSD | ExpGr (18–48); CtrlGr (22–62) | N/R | N/R | N/R | Positive sx (PANSS positive); Cognitive bias (BADE) | N/A |
| Andreou | 2017 | ExpGr: SSD(46); CtrlGr: SSD (46) | N/R | N/R | ExpGr (344.56); CtrlGr (305.49) | ExpGr (424); CtrlGr (393.5) | Delusions (PSYRATS delusions, PANSS: P1); Positive sx (PANSS positive); Negative sx (PANSS negative); Cognitive bias (Fish task variants); Self-esteem (RSES); QoL (WHOQOL: Physical, Psychological; WHOQOL BREF: Psychological, Relations, Environment) | 24 |
| Andreou | 2018 | ExpGr schizophrenia (26) | N/R | 50% | 828.15 | 806.6 | Positive sx (PANSS positive); Delusions (PANSS: P1); Negative sx (PANSS negative); Cognitive bias (Fish task, Box task variants) | N/A |
| Balzan | 2014 | ExpGr: schizophrenia (14); CtrlGr: schizophrenia (14) | N/R | 100% | N/R | N/R | Positive sx (PANSS positive, SAPS); Delusions (PANSS: P1, PDI); QoL (WHOQOL: Psychological, Social); Cognitive bias (Representativeness task variants, Illusion of Control task variants) | N/A |
| Balzan | 2019 | ExpGr schizophrenia (18), schizoaffective (6), other psychotic disorder (3); | N/R | 96% | ExpGr (609.11); CtrlGr (425) | ExpGr (420.7); CtrlGr (361.68) | Positive sx (PANSS positive); Delusions (PANSS: P1, PSYRATS delusions); Negative sx (PANSS negative); Cognitive | N/A |

| First Author(s) | Year | Diagnosis | Age Range | % N Antipsy | Dose EQ (mean) | Dose EQ (SD) | Outcome (measure) | FUP |
|---|---|---|---|---|---|---|---|---|
| Balzan *(continued)* | 2019 | CtrlGr schizophrenia (20), schizoaffective (5), other psychotic disorder (2) | | | | | bias (Beads task: Jumping to conclusions) | |
| Balzan | 2019 | ExpGr schizophrenia (18), schizoaffective (6), other psychotic disorder (3); CtrlGr schizophrenia (20), schizoaffective (5), other psychotic disorder (2) | N/R | 96% | ExpGr (609.11); CtrlGr (425) | ExpGr (420.7); CtrlGr (361.68) | Positive sx (PANSS positive); Delusions (PANSS: P1, PSYRATS delusions); Negative sx (PANSS negative); Cognitive bias (Beads task: Jumping to conclusions) | 224 |
| Briki [a] | 2014 a | SSD | N/R | 100% | N/R | N/R | Functioning (ILSS social relationships, total) | N/A |
| Briki | 2014 b | SSD | N/R | 100% | ExpGr (1519); CtrlGr (1359) | ExpGr (1635); CtrlGr (1516) | Positive sx (PANSS positive); Hallucinations (PANSS: P3, PSYRATS hallucinations); Delusions (PANSS: P1, P6; PSYRATS delusions); Functioning (QLS, Interpersonal Relations Subscales: social initiatives, social circle) | N/A |
| Chen | 2021 | ExpGr schizophrenia (58); CtrlGr schizophrenia (62) | N/R | N/R | N/R | N/R | Positive sx (PANSS positive, PSYRATS total); Delusions (PSYRATS delusions, PANSS core delusions); Hallucinations (PSYRATS hallucinations); Negative sx (PANSS negative); QoL (SQLS: Psychosocial, Motivation and energy, Symptoms and side effects) | N/A |
| de Pinho | 2020 | schizophrenia | N/R | 100% | N/R | N/R | Positive sx (PSYRATS total); Delusions (PSYRATS delusions); Hallucinations (PSYRATS hallucinations); | 12 |
| First Author(s) | Year | Diagnosis | Age Range | % N Antipsy | Dose EQ (mean) | Dose EQ (SD) | Outcome (measure) | FUP |

| | | | | | | | | |
|---|---|---|---|---|---|---|---|---|
| de Pinho *(continued)* | 2020 | | | | | | Functioning (PSP, WHODAS 2.0) | |
| Erawati | 2014 | SSD | N/R | N/R | N/R | N/R | Delusions (PSYRATS delusions) | N/A |
| Favrod | 2011 | schizophrenia (16); schizoaffective (2) | N/R | 100% | N/R | N/R | Delusions (PANSS: P1, PSYRATS delusions); Hallucinations (PANSS: P3, PSYRATS hallucinations) | N/A |
| Favrod [b] | 2014 | ExpGr schizophrenia (21) schizoaffective (5); CtrlGr schizophrenia (22) schizoaffective disorders (4) | N/R | 100% | ExpGr (422); CtrlGr (379) | ExpGr (218); CtrlGr (163) | Positive sx (PANSS positive); Delusions (PSYRATS delusions) | 26 |
| Fekete | UD | SSD | N/R | 100% | ExpGr (14.26); CtrlGr (11.43) | ExpGr (9.40); CtrlGr (6.20) | Positive sx (PANSS positive); Negative sx (PANSS negative) | 8 |
| Ferwerda | 2010 | paranoid schizophrenia (29) | N/R | 100% | N/R | N/R | Delusions (DRS total, GPTS total); Cognitive bias (Beads task variants); Self-esteem (SERS-SF 20 positive, negative) | N/A |
| Fujii [c] | 2021 | schizophrenia | N/R | 100% | GrA (1117.24); GrB (1033.25) | GrA (686.94); GrB (419.74) | Positive sx (PANSS positive); Negative sx (PANSS negative); Functioning (GAF) | N/A |
| Gaweda | 2015 | ExpGr schizophrenia (23); CtrlGr schizophrenia (21) | N/R | 100% | ExpGr (531.75); CtrlGr (440) | ExpGr (389.23); CtrlGr (372.34) | Delusions (PSYRATS delusions, Paranoia checklist: frequency, conviction conviction, distress); Hallucinations (PSYRATS hallucinations); Cognitive bias (CBQp total) | N/A |
| Ishikawa | 2020 | ExpGr schizophrenia disorders (25); schizotypal (1); CtrlGr schizophrenia disorders (23); schizoaffective (1) | N/R | N/R | ExpGr (720.82); CtrlGr (804.97) | ExpGr (402.27); ExpGr (505.32) | Positive sx (PANSS positive); Delusions (PANSS core delusions); Hallucinations (PANSS: P3); Cognitive bias; (CBQ)(jumping to conclusions); Functioning (GAF); QoL (EQ-5D-EL); Self-esteem (RSES) | 4 |

| First Author(s) | Year | Diagnosis | Age Range | % N Antipsy | Dose EQ (mean) | Dose EQ (SD) | Outcome (measure) | FUP |
|---|---|---|---|---|---|---|---|---|
| Kowalski [c] | 2017 | 29 paranoid schizophrenia, 1 unspecified schizophrenia, 1 acute polymorphic psychotic disorder with schizophrenia symptoms | N/R | N/R | ExpGr (810); CtrlGr (797) | ExpGr (362); CtrlGr (351) | Delusions (Paranoia Checklist: Intensity, Conviction, Distress); Cognitive bias (Fish task variants) | N/A |
| Kumar | 2010 | paranoid schizophrenia | N/R | 100% | N/R | N/R | Positive sx (PANSS positive); Delusions (BABS); Negative sx (PANSS negative) | N/A |
| Kumar | 2015 | paranoid schizophrenia | N/A | N/A | N/R | N/R | Positive sx (PANSS positive); Delusions (BABS); Negative sx (PANSS negative) | N/A |
| Kuokkanen | 2014 2015 | ExpGr schizophrenia (10 with 4 forensic and 6 non-forensic); CtrlGr (10 with 6 forensic and 4 non-forensic) | ExpGr (28-56); CtrlGr (19-67) | N/R | N/R | N/R | Positive sx (PANSS positive); Delusions (PANSS: P1, PSYRATS: Preoccupation. Duration preoccupation, Conviction, Amount of distress, Intensity of distress, Disruption); Cognitive bias (Fish task variants); QoL (15D: Depression, Distress, Index) | 12; 24 |
| Lopez-Morinigo | UD* | ExpGr schizophrenia (23), other SSD (16); CtrlGr schizophrenia (25), other SSD (13) | N/R | N/R | ExpGr (442.3); CtrlGr (461.2) | ExpGr (310); CtrlGr (387.1) | Positive sx (PANSS positive); Negative sx (PANSS negative); Cognitive bias (Beads task variants); Functioning (GAF, World Health Organization Disability Schedule); QoL (Satisfaction Life Domains Scale) | 40 |
| Moritz | 2011 a | SSD | N/R | N/R | ExpGr (65.42); CtrlGr (66.76) | ExpGr (46.32); CtrlGr (42.86) | Positive sx (PANSS positive); Delusions (PSYRATS delusions); Hallucinations (PSYRATS hallucinations); | N/A |
| Moritz *(continued)* | 2011 a | | | | | | Negative sx (PANSS negative); | |

| | | | | | | | | |
|---|---|---|---|---|---|---|---|---|
| | | | | | | | Cognitive bias (Fish task variants); QoL (WHOQOL-BREF) | |
| Moritz | 2011 b | SSD | N/R | 78% | N/R | N/R | Delusions (PANSS core delusions, PSYRATS delusions); Hallucinations (PSYRATS hallucinations); Cognitive bias (Beads task variant) | N/A |
| Moritz | 2013 | SSD | N/R | 100% | ExpGr (69.72%); CtrlGrp (80.96%) | ExpGr (59.06); CtrlGrp (63.37) | Positive sx (PANSS positive); Delusions (PANSS core delusions, PSYRATS delusions); Hallucinations (PSYRATS hallucinations); Cognitive bias (Fish task variants); Self-esteem (RSES) | 24 |
| | 2014 | | | | ExpGr (72.37%); CtrlGrp (79.71%) | ExpGr (61.59); CtrlGrp (63.20) | Self-esteem (RSES); QoL (WHOQOL-BREF: total) | 156 |
| | 2018 | | | | ExpGr (69.72%); CtrlGrp (80.96%) | ExpGr (59.06); CtrlGrp (63.37) | QoL (WHOQOL total); Self-esteem (RSES); Cognitive bias (CBQp total, Number of admissions); Self-esteem (RSES) | 24; 156 |
| Naughton | 2012 | ExpGr schizophrenia (7), schizoaffective (3), major depression with psychotic features (7); CtrlGr schizophrenia (8) | N/R | 100% | N/R | N/R | Positive sx (PANSS positive); Negative sx (PANSS negative); Functioning (GAF) | N/A |

| First Author(s) | Year | Diagnosis | Age Range | % N Antipsy | Dose EQ (mean) | Dose EQ (SD) | Outcome (measure) | FUP |
|---|---|---|---|---|---|---|---|---|
| Ochoa Salas-Sender | 2017 2020 | SSD | N/R | 100% | ExpGr (472.53); CtrlGr (519.49) | ExpGr (703.89); CtrlGr (534.58) | Positive sx (PANSS positive); Negative sx (PANSS negative); Functioning (GAF); Cognitive bias (IPSAQ: Externalizing bias Personalizing bias, Beads task variants) | |
| Ochoa | 2020 | SSD | N/R | 100% | N/R | N/R | Positive sx (PANSS positive); Delusions (PANSS: P1); Negative sx (PANSS negative); Functioning (GAF); Self-esteem (RSES) | N/A |
| Park | 2020 | schizophrenia | N/R | N/R | N/R | N/R | Positive sx (SAPS); Negative sx (SANS); Functioning (Relationship Change Scale) | N/A |
| Pos | 2018 | ExpGr schizophrenia or schizophreniform (15), psychotic disorder NOS (3), schizoaffective (2), other disorder with psychotic sx (5); CtrlGr schizophrenia or schizophreniform disorder (15), psychotic disorder NOS (6) schizoaffective (1), other disorder with psychotic symptoms (3) | N/R | ExpGr (95%); CtrlGr (96%) | N/R | N/R | Delusions (Experience Sampling Measure: Paranoid ideation, Delusional conviction); Cognitive bias (Beads task: Jumping to Conclusions) | N/A |
| Raucher-Chene | UD | schizophrenia (4); schizoaffective (2); dissociative disorder (1); bipolar I (1); major depressive disorder (1); other specified SSD (1); unspecified SSD (4) | (20-51) | 91.67% | (397.0) | (226.2) | Positive sx (PANSS-6 total); Wellbeing (WEMWBS); Functioning (PSP); Cognitive bias (Beads task variants, DACOBS); QoL (Q-LES-Q 18) | N/A |
| Schneider | 2018 | schizophrenia (100); brief psychotic disorder (13); bipolar disorder (29); schizoaffective (23); other (11) | N/R | 95% | N/R | N/R | Positive sx (questionnaire developed for study: positive symptoms); Cognitive bias (questionnaire developed for study: Jumping to conclusions, BADE) | N/A |

| First Author(s) | Year | Diagnosis | Age Range | % N Antipsy | Dose EQ (mean) | Dose EQ (SD) | Outcome (measure) | FUP |
|---|---|---|---|---|---|---|---|---|
| Shan | 2021 | schizophrenia | 18-38 | 100% | N/R | N/R | Positive sx (PANSS positive); Negative sx (PANSS negative) | N/A |
| Simon-Exposito | 2019 | ExpGr schizophrenia (11); CtrlGr schizophrenia (11) | N/R | N/R | N/R | N/R | Positive sx (PANSS: G12, P6); Delusions (PANSS: P1); Hallucinations (PANSS: P3) | N/A |
| So | 2015 | schizophrenia (25); delusional disorder (8); schizoaffective (1) psychotic disorder NOS (3); depression with psychotic symptoms (3); bipolar disorder (1); unknown (3) | N/R | 98% | ExpGr (217.36); CtrlGr (336.79) | ExpGr (172.37); CtrlGr (248.41) | Positive sx (PANSS positive); Delusions (PANSS: P1, PSYRATS delusions); Cognitive bias (MADS, Beads task variants) | 4 |
| So | 2021 | schizophrenia (36); delusional disorder (18); psychotic disorder NOS (2) | N/R | N/R | (492.04) | (415.64) | Positive sx (PANSS positive); Delusions (PSYRATS delusions); Negative sx (PANSS negative); Cognitive bias (MADS, BADE tasks) | 4; 24 |
| Tanoue [d] | 2021 | schizophrenia | N/R | 36.36% | 347.3 | 187.4 | Positive sx (PANSS positive); Cognitive bias (CBQp jumping to conclusions, total); QoL (EuroQOL 5 dimensions 5-level); Functioning (GAF) | 4 |
| Ussorio | 2016 | ExpGr SSD or affective psychosis (56) | N/R | 100% | ExpGr (109,9); CtrlGr (114,4) | ExpGr (65); CtrlGr (79,7) | Positive sx (PANSS positive); Negative sx (PANSS negative); Functioning (PSP, SNQ); Self-esteem (SERS) | N/A |
| van Oosterhout | 2014 | ExpGr schizophrenia (52), psychotic disorder NOS (9), schizoaffective (3), other (11); CtrlGr schizophrenia (46), psychotic disorder NOS (9), schizoaffective (5), other (19) | 16-55 | ExpGr (93%); CtrlGr (91%) | ExpGr (379.5); CtrlGr (284) | Standard error: ExpGr (70,5); CtrlGr (38) | Delusions (DRS, GPTS); Cognitive bias (DACOBS: Subjective cognitive problems, Social cognition problems) | 16 |

| First Author(s) | Year | Diagnosis | Age Range | % N Antipsy | Dose EQ (mean) | Dose EQ (SD) | Outcome (measure) | FUP |
|---|---|---|---|---|---|---|---|---|
| Yildiz | 2019 | ExpGr schizophrenia (10); CtrlGr schizophrenia (10) | N/R | N/R | N/R | N/R | Positive sx (CGI-Severity); Functioning (GAF, QLS) | N/A |
| Zalzala | 2019 | ExpGr 50% schizoaffective, 50% schizophrenia; CtrlGr 53% schizoaffective, 47% schizophrenia | N/R | ExpGr (69); CtrlGr (75) | N/R | N/R | Positive sx (BPRS positive); Negative sx (BPRS negative) | 8 |

*Note.* % N Antipsy = percentage of the sample taking antipsychotic medication(s); Dose EQ = chlorpromazine equivalent; FUP = length from post evaluation to follow-up, in weeks; ExpGr = experimental group (i.e., metacognitive training); CtrlGr = control group; sx = symptoms; N/R = not rated; N/A = not applicable; outcome measure: see eTable 3 for measure acronyms; SSD = schizophrenic spectrum disorder; UD = unpublished data; QoL = quality of life; a = antipsychotic medication % inferred from "Participants were on stable neuroleptic medication"; b = antipsychotic medication %: ambiguous, but stable dose of antipsychotic was an inclusion criterion; c = Crossover study; d = Transdiagnostic study wherein 22 (64.7%) of participants had schizophrenia, outcome data is schizophrenia-specific; *for more information regarding unpublished data from Lopez-Morinigo contact jlmorinigo@salud.madrid.org

**eTable 6. Metacognitive training (MCT) intervention characteristics of included studies.**

| Study First Author(s) | Year | Sessions | Sessions per week | Session Duration | Format | Facilitator Credentials | Other Facilitator Credentials | Number of Facilitators | Facilitator Training | Manual Adherence |
|---|---|---|---|---|---|---|---|---|---|---|
| Acuna | 2021 | 10 | 1 | 45-60 | Group | Psychiatrist | Psychologist | 2 | N/R | Yes |
| Aghotor | 2010 | 8 | 2 | 45-60 | Group | N/R | N/R | N/R | Yes | No [a] |
| Andreou | 2017 | 12 | 2 | 45-60 | INDV | Psychologist | N/A | N/R | N/R | N/R |
| Andreou | 2018 | 8 | 2 | 45-60 | Group | N/R | N/R | N/R | N/R | N/R |
| Balzan | 2014 | 1 | 1 | 60 | INDV | N/R | N/R | N/R | N/R | No [g] |
| Balzan | 2019 | 4 | 1 | 120 | INDV | Psychologist | N/R | N/R | Yes | Yes |
| Briki | 2014 (a) | 10 | 1 or 2 [b] | 60 | Group | N/R | N/R | 2 | Yes | N/R |
| Briki | 2014 (b) | 16 | 2 | 60 | Group | Psychiatrist | Psychiatric nurses; Physician interns; Psychologists | N/R | Yes | Yes |
| Chen | 2021 | 8 | 1 | 60 | Group [c] | General practitioner | N/A | 1 | Yes | N/R |
| de Pinho | 2020 | 8 | 2 | 45-60 | Group | Nurse | N/A | N/R | Yes | Yes |
| Erawati | 2014 | 8 | 2 | 45-60 | INDV | Nurse | N/A | 1 | N/R | N/R |
| Favrod | 2011 | 8 or 16 [d] | 1 | 60 | Group | Nurse | Research assistant; Nursing assistant | 2 or 3 | N/R | N/R |

| Study First Author(s) | Year | Sessions | Sessions per week | Session Duration | Format | Facilitator Credentials | Other Facilitator Credentials | Number of Facilitators | Facilitator Training | Manual Adherence |
|---|---|---|---|---|---|---|---|---|---|---|
| Favrod | 2014 | 8 | 1 | 60 | Group | N/R | N/R | N/R | N/R | N/R |
| Fekete | UD | 16 | 1 | 45-60 [e] | Group | N/R | N/A | 1 | N/R | Yes |
| Ferwerda | 2010 | 8 | 2 [f] | 45-60 | Group | N/R | N/R | N/R | N/R | Yes |
| Fujii | 2021 | 16 | 1 | 60 | Group | Occupational therapist | Occupational therapist | 2 | N/R | Yes |
| Gaweda | 2015 | 8 | 2 | 45-60 | Group | Graduate student | N/R | NR | Yes | Yes |
| Ishikawa | 2020 | 10 | 1 | 45-60 | Group | Occupational therapist | Psychiatrist; Psychiatric nurse | N/R | Yes | Yes |
| Kowalski | 2017 | 1 | 1 | N/R | Group | N/R | N/R | N/R | N/R | No [g] |
| Kumar | 2010 | 8 [h] | 2 | 45-60 | Group [h] | N/R | N/R | N/R | N/R | N/R |
| Kumar | 2015 | 12 | N/R | 45-60 | INDV | N/R | N/R | N/R | N/R | Yes |
| Kuokkanen<br>Kuokkanen | 2014<br>2015 | 8 | 2 | 45 | Group | Psychologist | N/A | 2 | Yes | Yes |
| Lopez-Morinigo | UD | 8 | 1 | 45-60 | Group | Psychologist | N/A | 1 | Yes | Yes |
| Moritz | 2011 (a) | 8 | 1 | 45-60 | Group | Psychologist | Graduate student | N/R | N/R | Yes |
| Moritz | 2011 (b) | MCT; MCT+ [i] | 2 | 45-60 | Group; INDV | Psychologist | MCT (Intern) | MCT (2); MCT+ (1) | N/R | N/R |
| Moritz<br>Moritz<br>Moritz | 2013<br>2014<br>2018 | 8 to16 [j] | 2 | 45-60 | Group | Psychologist | Psychologist trainees | N/R | Yes | Yes |

| Study First Author(s) | Year | Sessions | Sessions per week | Session Duration | Format | Facilitator Credentials | Other Facilitator Credentials | Number of Facilitators | Facilitator Training | Manual Adherence |
|---|---|---|---|---|---|---|---|---|---|---|
| Naughton | 2012 | 16 | 2 | N/R | Group | Psychiatrist | Clinical nurse specialist | 2 | Yes | N/R |
| Ochoa Salas-Sender | 2017 2020 | 8 | 1 | 60 | Group | Psychologist | Psychiatrist; Nurse | 2 | Yes | Yes |
| Ochoa | 2020 | 10 | 1 | N/R | INDV | Psychologist | Psychologist; Psychiatrist [k] | 1 | Yes | Yes |
| Park | 2020 | 18 | 1 to 2 | 60 | N/R | N/R | N/R | N/R | N/R | N/R |
| Pos | 2018 | 8 | 1 | N/R [l] | Group | Nurse | N/A | 1 | Yes | Yes |
| Raucher-Chene | UD | 12 | 2 | 45-60 | Group-virtual | Psychologist | Psychiatrist | ≥ 2 | Yes | Yes |
| Schneider | 2018 | ≤ 20 | 2 | N/R | Group | Graduate student | N/R | 2 | Yes | N/R |
| Shan | 2021 | 8 | 1 | 45-60 | Group | Psychiatrist | N/A | 1 | Yes | Yes |
| Simon-Exposito | 2019 | 16 | 2 | N/R | Group | Psychologist | N/R | 2 | Yes | Yes |
| So | 2015 | 4 | 1 | 60 | INDV | Psychologist | N/R | 1 | Yes | Yes |
| So | 2021 | 4 | 1 | 45-60 | Group | Psychologist | Psychiatrists | N/R | N/R | Yes |
| Tanoue | 2021 | 10 | 1 | 60 | Group | Nurse | Occupational therapist; Psychiatric social worker | 1 or 2 | Yes | N/R |
| Ussorio | 2016 | 16 | 1 | 45-60 | Group | Psychologist | Psychiatric rehabilitation technician | 2 | Yes | Yes |

| Study First Author(s) | Year | Sessions | Sessions per week | Session Duration | Format | Facilitator Credentials | Other Facilitator Credentials | Number of Facilitators | Facilitator Training | Manual Adherence |
|---|---|---|---|---|---|---|---|---|---|---|
| van Oosterhout | 2014 | 8 | 1 | N/R | Group | Psychologist | Psychiatrist; Occupational therapist; Psychiatric nurse | 2 | Yes | Yes |
| Yildiz | 2019 | 40 | 2 | 40-50 | Group | N/R | N/R | 2 | Yes | N/R |
| Zalzala | 2019 | 8 | 1 | N/R | Group | N/R | N/R | N/R | N/R | N/R |

*Note.* Reports presented in the same row are from the same study trial; MCT = metacognitive training; MCT + = individualized MCT; N/R = not rated in the study; N/A = not applicable; UD = unpublished data; a = manual was updated during the intervention; b = inferred given 10 sessions were administered over 8 weeks; c = participants were compensated $5 after each session; d = participants completed either 1 (8 week) or 2 (16 week) cycles over 8 months; e = inferred given standard metacognitive training was reported; f = inferred given description of MCT in introduction; g = 1 MCT session delivered; h = inferred given 2 sessions per week for 4 weeks and implied in description of MCT in introduction; i = 1 MCT+ session related to medical history; j = all participants received a maximum of 8 sessions prior to post assessment and were invited to complete another cycle of 8 sessions immediately thereafter; k = facilitator credentials at other cites; l = a link to the study protocol (in Dutch) was provided in the manuscript.

**eFigure 1. Forest plots by outcome for the pre-post comparisons.**
**Effect sizes (Hedges' g) and their 95% confidence intervals for each study/report, with positive values favoring MCT and negative values favoring the control condition.**

(a) Proximal outcomes

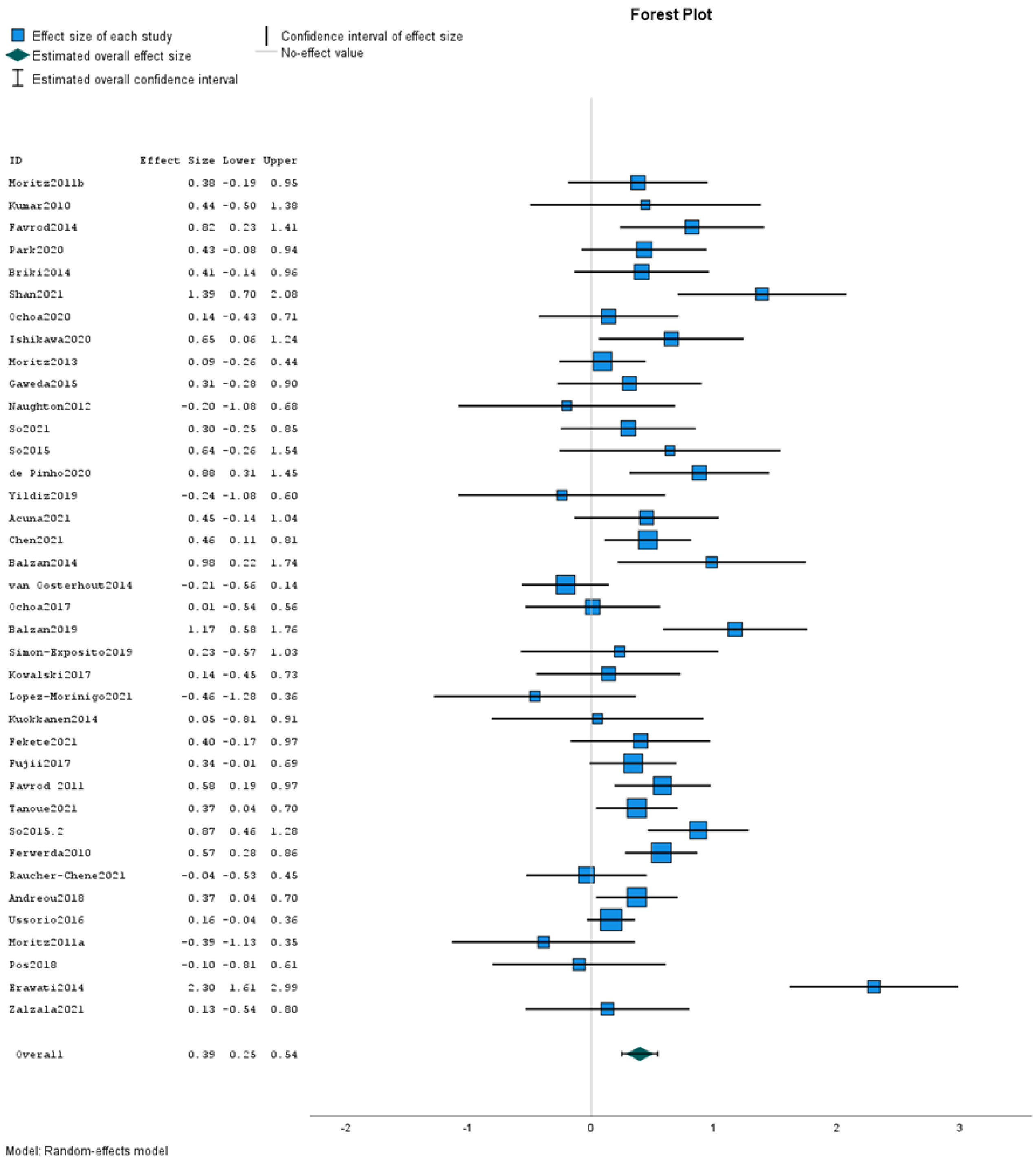

## (b) Positive symptoms

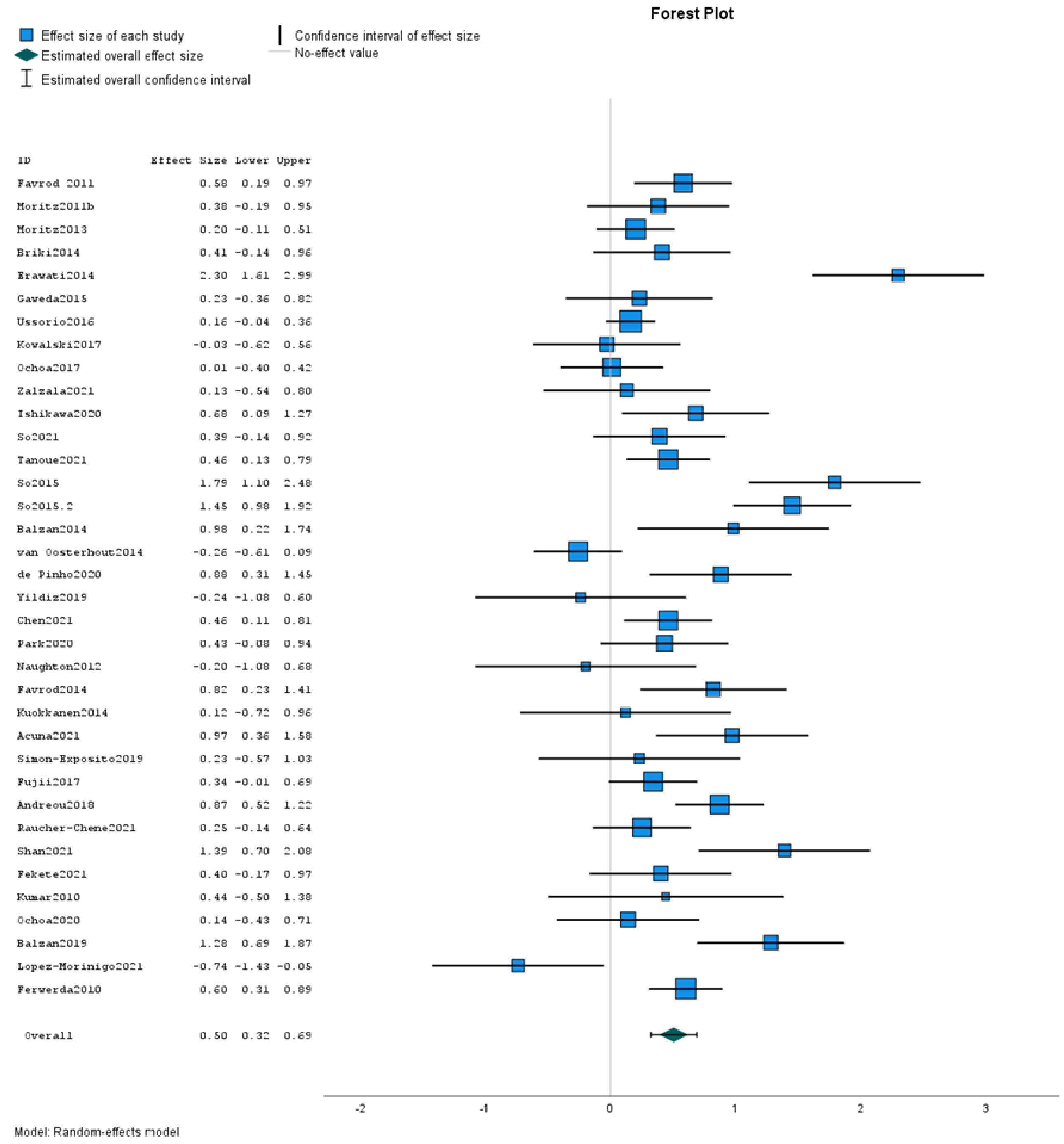

## (c) Delusions

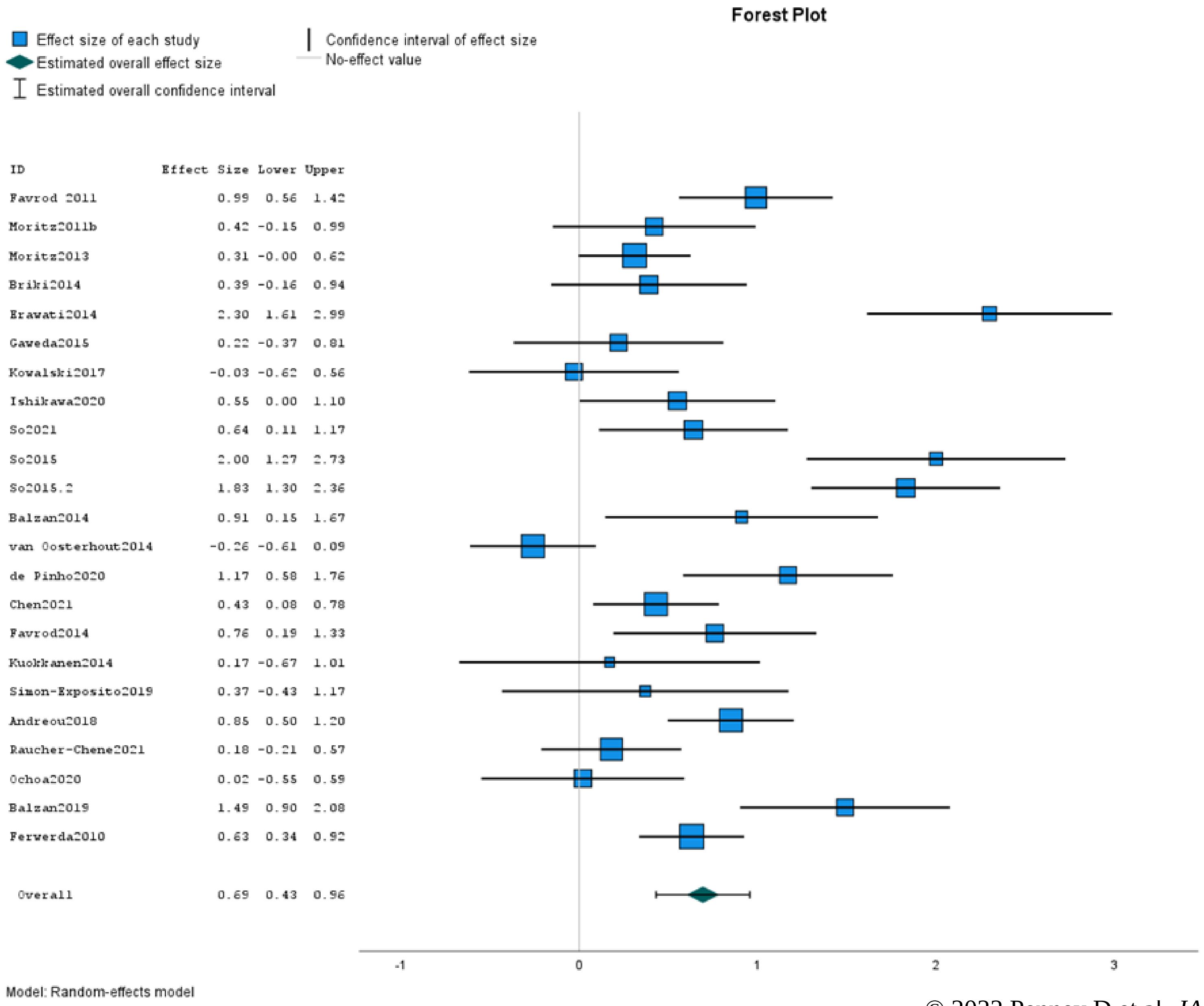
Forest Plot
Effect size of each study
Estimated overall effect size
Estimated overall confidence interval
Confidence interval of effect size
No-effect value
ID Effect Size Lower Upper
Favrod 2011 0.99 0.56 1.42
Moritz2011b 0.42 -0.15 0.99
Moritz2013 0.31 -0.00 0.62
Briki2014 0.39 -0.16 0.94
Erawati2014 2.30 1.61 2.99
Gaweda2015 0.22 -0.37 0.81
Kowalski2017 -0.03 -0.62 0.56
Ishikawa2020 0.55 0.00 1.10
So2021 0.64 0.11 1.17
So2015 2.00 1.27 2.73
So2015.2 1.83 1.30 2.36
Balzan2014 0.91 0.15 1.67
van Oosterhout2014 -0.26 -0.61 0.09
de Pinho2020 1.17 0.58 1.76
Chen2021 0.43 0.08 0.78
Favrod2014 0.76 0.19 1.33
Kuokkanen2014 0.17 -0.67 1.01
Simon-Exposito2019 0.37 -0.43 1.17
Andreou2018 0.85 0.50 1.20
Raucher-Chene2021 0.18 -0.21 0.57
Ochoa2020 0.02 -0.55 0.59
Balzan2019 1.49 0.90 2.08
Ferwerda2010 0.63 0.34 0.92
Overall 0.69 0.43 0.96
-1
0
1
2
3
Model: Random-effects model

(d) Hallucinations

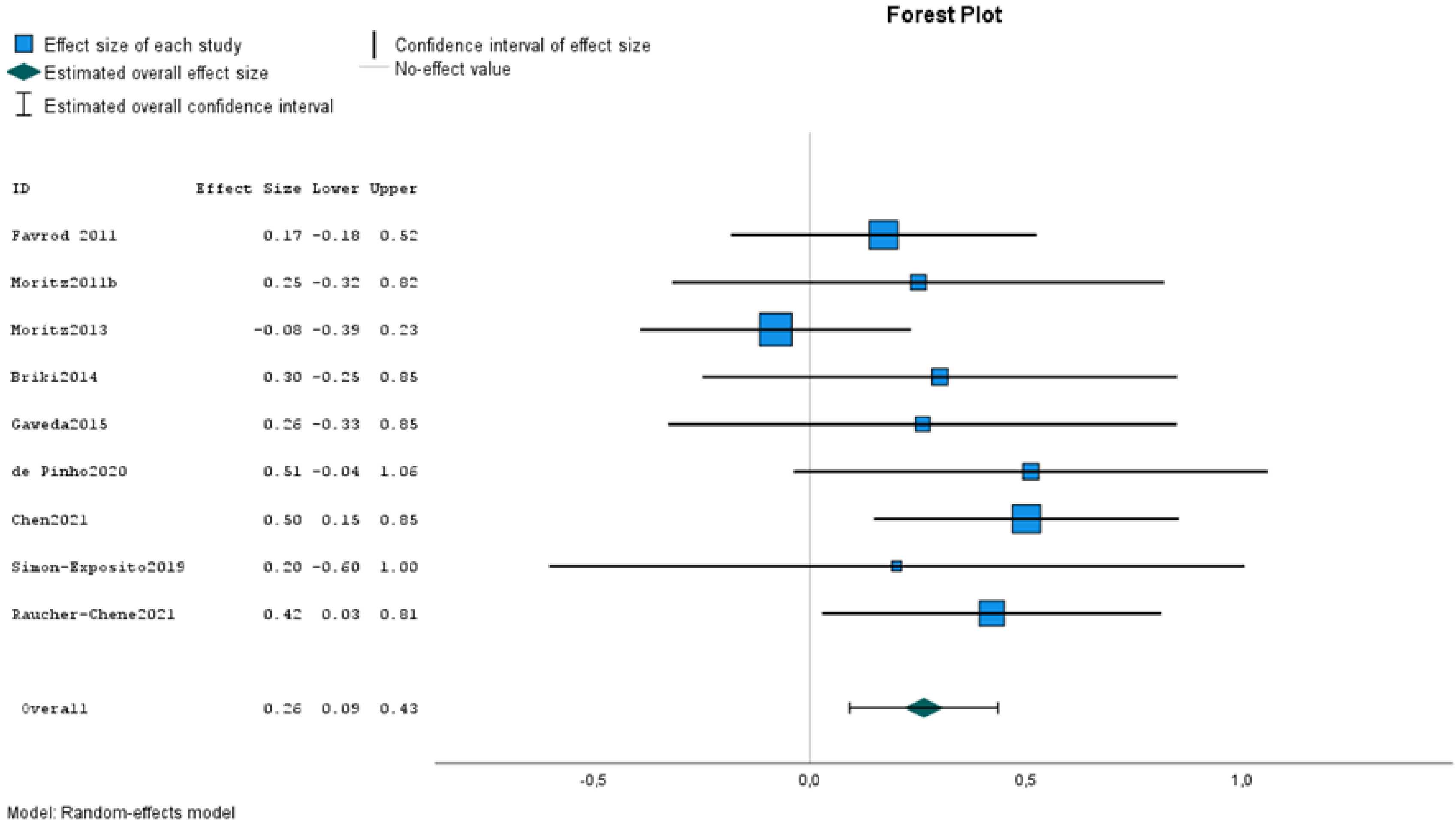

(e) Cognitive bias

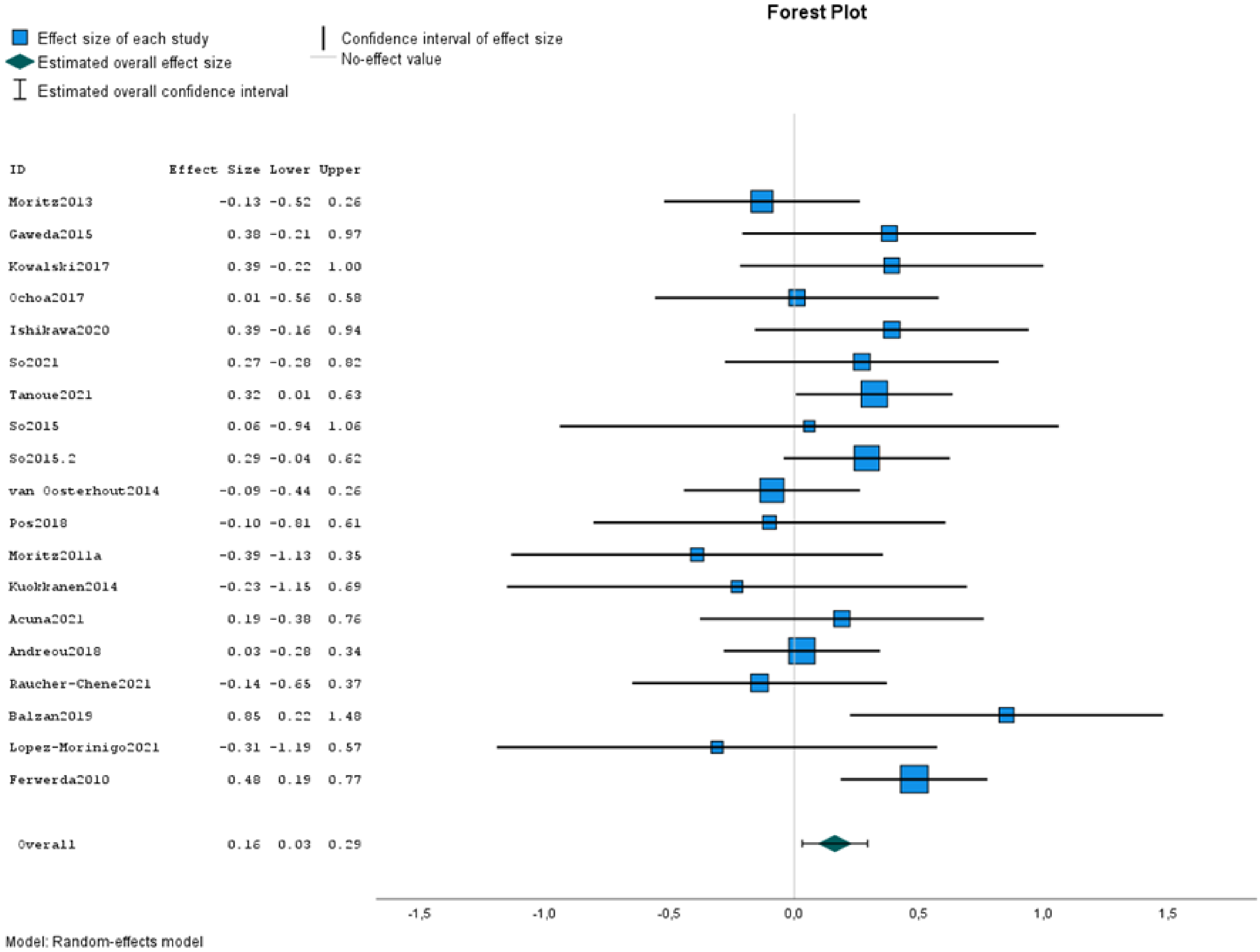

(f) Distal outcomes

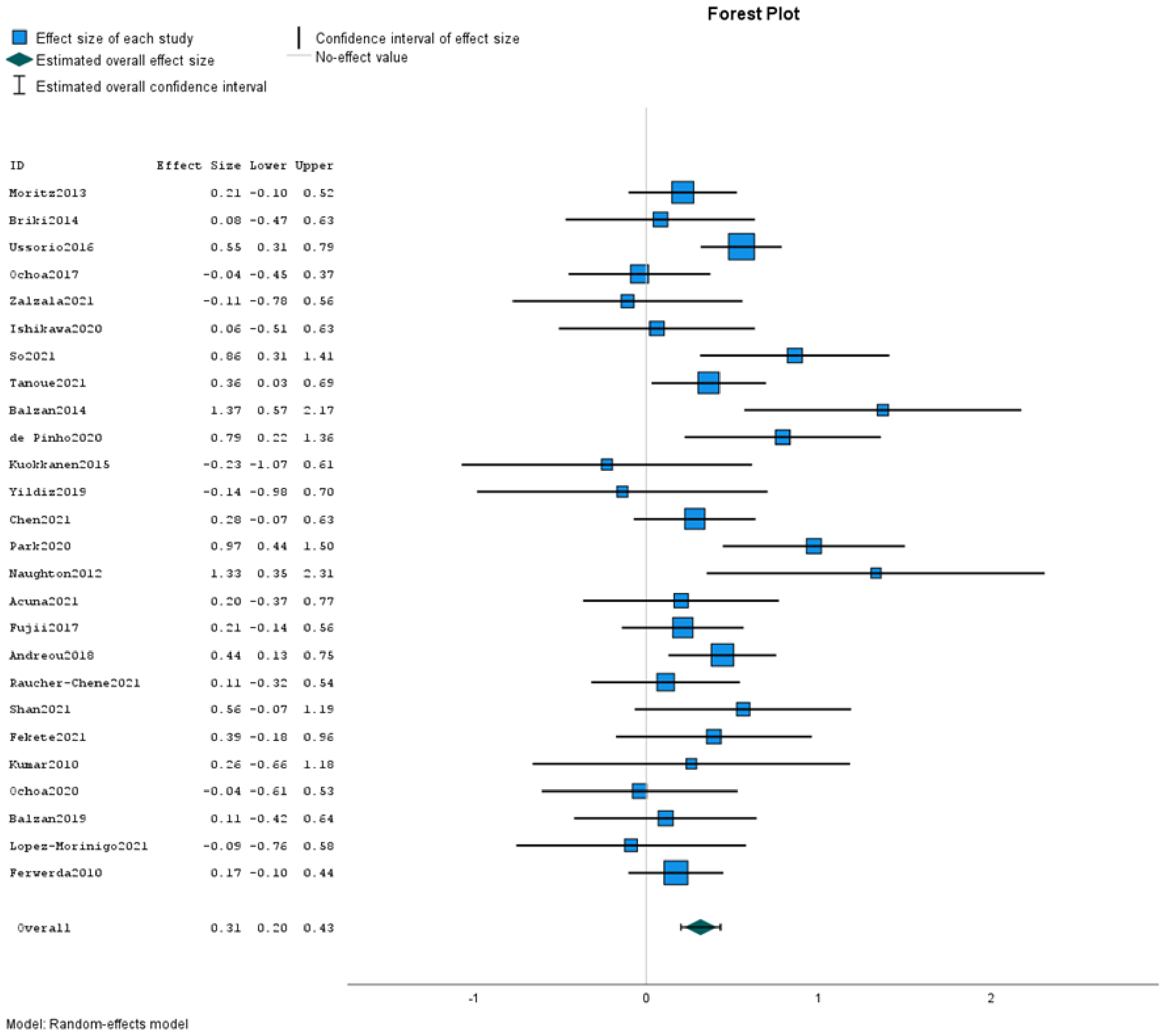

(g) Negative symptoms

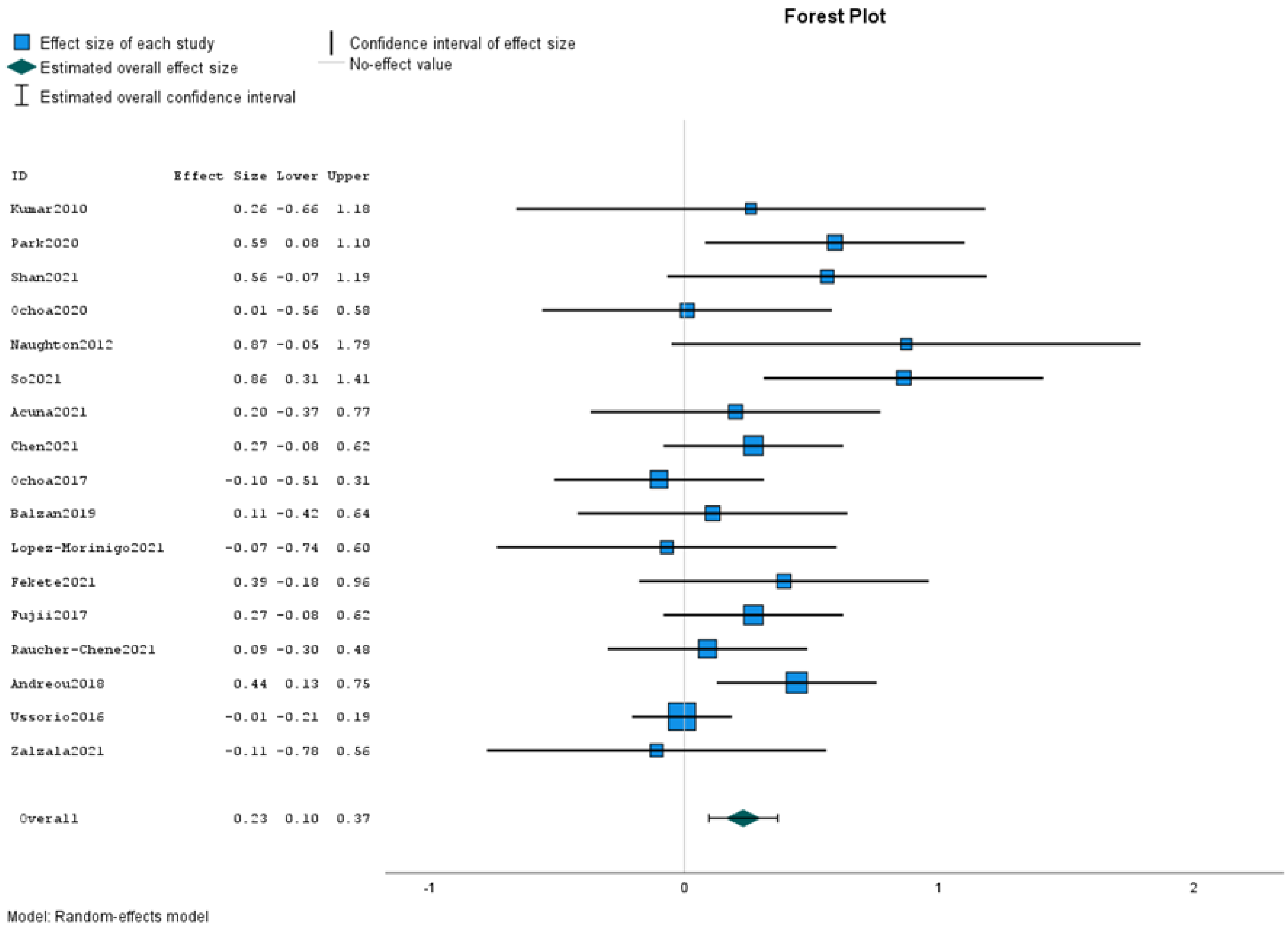

(h) Self-esteem

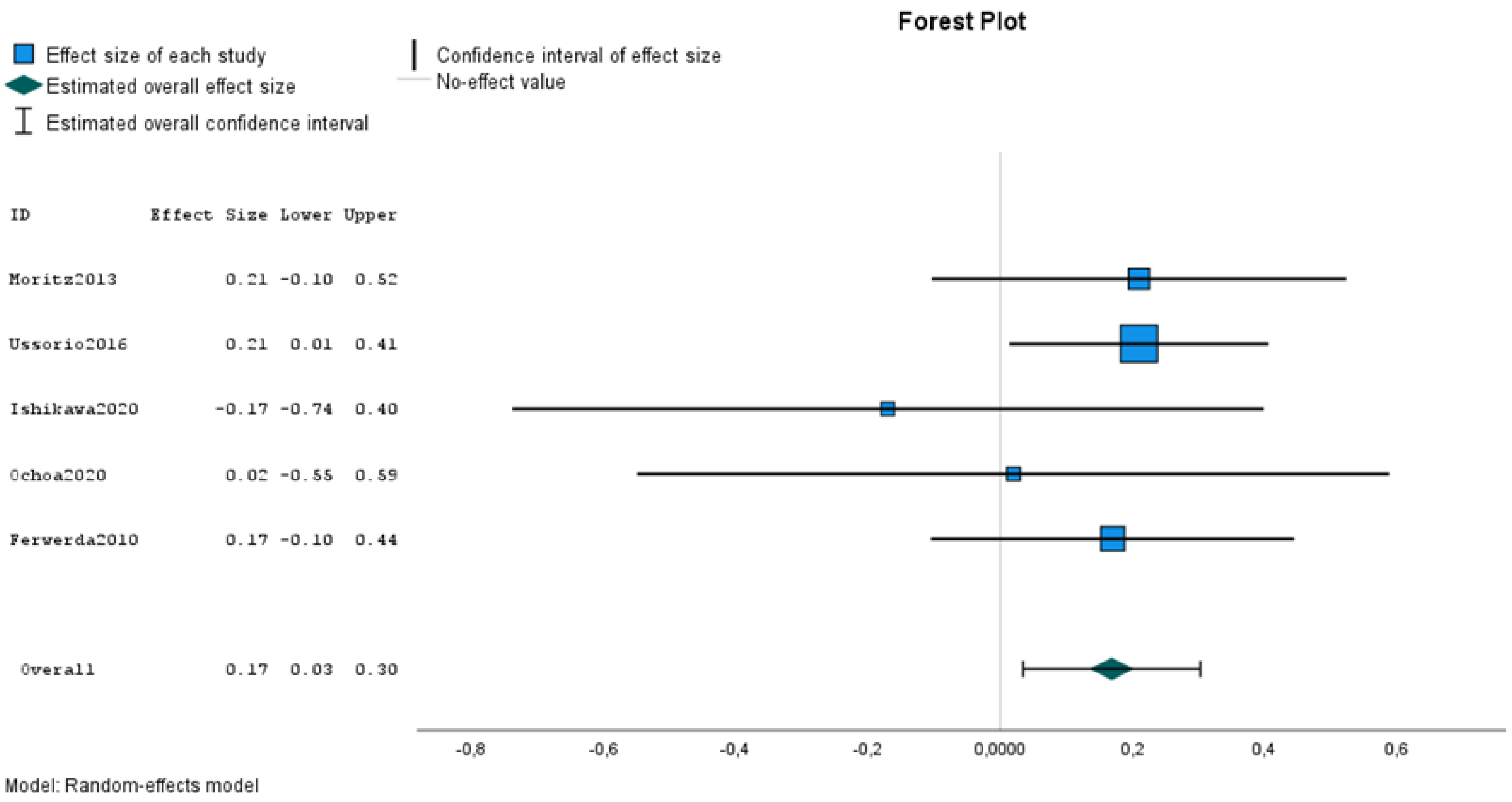

(i) Quality of life

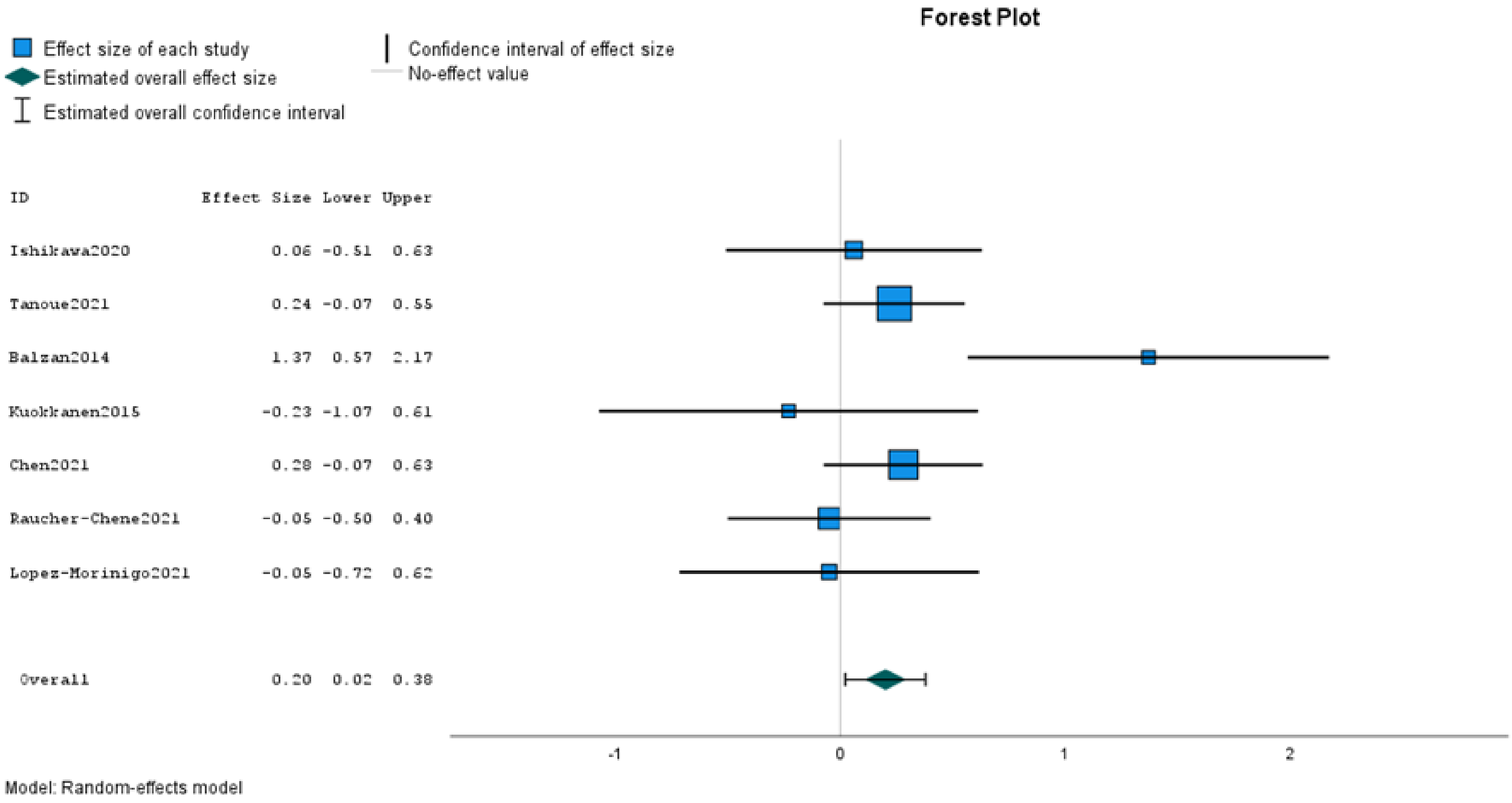

(j) Functioning

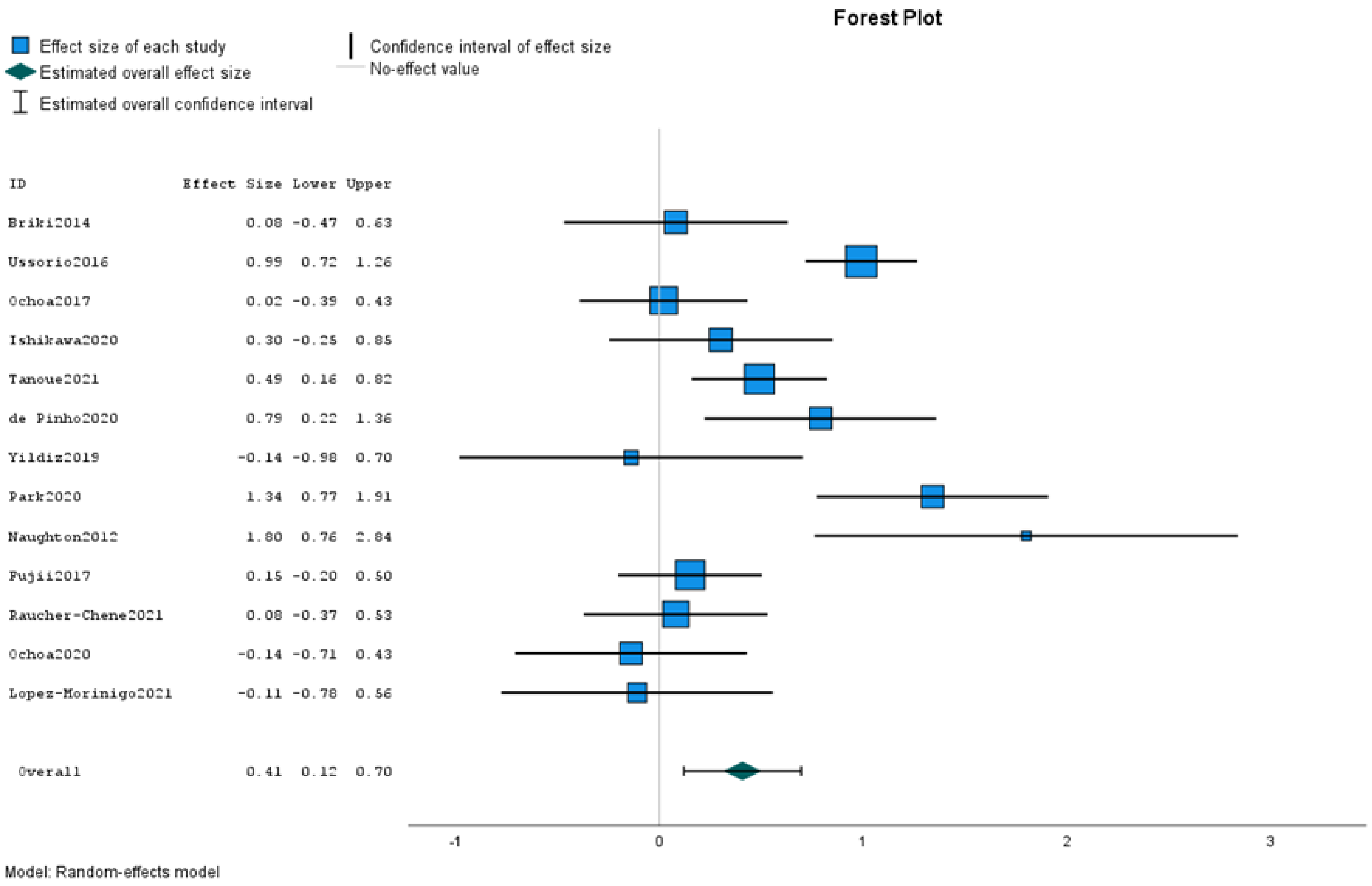

## eTable 7. List of excluded studies and ongoing trials.

| First Author | Year/ Trial # | Title | Reason for Exclusion/ Link to protocol |
|---|---|---|---|
| Aggarwal | CTRI/2018/ 02/011838 | Metacognitive therapy in schizophrenia | http://ctri.nic.in/Clinicaltrials/pmaindet2.php ?trialid=11094&EncHid=&userName=CTRI /2018/02/011838 ; principal author emailed no response |
| Ahuir | 2018 | Improvement in cognitive biases after group psychoeducation and metacognitive training in recent-onset psychosis: a randomized crossover clinical trial | Mixed intervention; MCT+ Psychoeducation in Group A; Group B psychoeducation + MCT |
| Akrap | 2013 | Metacognitive training for patients with schizophrenia (MCT): a Croatian pilot study of its efficacy | Conference abstract; author emailed no response |
| Alvarez-Astorga | 2019 | Social cognition in psychosis: Predictors and effects of META-cognitive training | Do not report + or - sx in the results, but did assess at baseline; senior author emailed no data provided |
| Andreou | NCT04631 939 | Metacognitive Training as a Serious Game | Ongoing trial; https://clinicaltrials.gov/ct2/show/NCT0463 1939 |
| Blumenfeld | Unknown | A longitudinal case-series study of the efficacy of metacognitive training at ameliorating psychosis-related symptoms and cognitive biases | Dissertation; Supervisor (Blumenfeld) emailed no response |
| Birulés | 2020 | Cognitive insight in first-episode psychosis: Changes during metacognitive training | Did not assess outcomes of interest |
| Buonocore | 2015 | Combined neurocognitive and metacognitive rehabilitation in schizophrenia: effects on bias against disconfirmatory evidence | Mixed intervention; used a combination of MCT and computer-assisted cognitive remediation |
| Caponnetto | 2018 | Improving neurocognitive functioning in schizophrenia by addition of cognitive remediation therapy to a standard treatment of metacognitive training | Mixed intervention; used a combination of MCT and cognitive remediation |
| Choi | 2014 | Case-management models for early psychosis intervention in Asia | Conference abstract; author emailed no response |
| Chong | 2016 | Metacognitive Training in Early Psychosis in Singapore: Preliminary Findings and Considerations | Conference abstract; author email not retrievable |

| First Author | Year/<br>Trial # | Title | Reason for Exclusion/<br>Link to protocol |
|---|---|---|---|
| Dobie | NCT03955549 | Insight Enhancement Program vs. Metacognitive Training for Psychosis in Patients With Schizophrenia: a Three-Armed Comparative Randomized Controlled Trial | Ongoing trial; https://clinicaltrials.gov/ct2/show/NCT03955549 |
| Fekete | 2021 | Basic demographic outcomes: additional findings of a single-blind, randomised, controlled trial on metacognitive training for psychosis | Received complete trial (unpublished) data from study author assessing more relevant outcomes |
| Howe | 2015 | Investigating the usefulness of a metacognitive training group programme for schizophrenia | Did not assess outcomes of interest |
| Kikuchi | N/A | Pilot trial of Metacognitive Training | UMIN000014554; principal author emailed no response |
| Kim | 2014 | Group cognitive behavioral therapy for Korean patients with early psychosis | Mixed intervention; used a combination of MCT with cognitive restructuring and lifestyle management |
| Lam | 2015 | Metacognitive training (MCT) for schizophrenia improves cognitive insight: a randomized controlled trial in a Chinese sample with schizophrenia spectrum disorders | Did not assess outcomes of interest |
| Lambert | NCT02037581 | Integrated Care Including Assertive Community Treatment in Early Psychosis | MCT is a part of an Integrated Care package; https://clinicaltrials.gov/ct2/show/record/NCT02037581?view=record |
| Lecardeur | 2009 | Effects of cognitive remediation therapies on psychotic symptoms and cognitive complaints in patients with schizophrenia and related disorders: A randomized study | Mixed intervention; MCT modules were integrated in the mental state attribution therapy (MSAT) |
| Kowalski | 2014 | Open therapeutic ward for young patients with psychotic disorders – Description and patients evaluation of therapeutic program | Did not assess outcomes of interest |
| Köther | 2017 | Bayesian analyses of the effect of metacognitive training on social cognition deficits and overconfidence in errors. | Did not assess outcomes of interest |
| Matsumoto | JPRN-UMIN000029146 2017 | Feasibility study of Meta Cognitive Training for At-Risk Mental State in Japan | Cancelled trial; https://upload.umin.ac.jp/cgi-open-bin/ctr_e/ctr_view.cgi?recptno=R000033325 |

| First Author | Year/ Trial # | Title | Reason for Exclusion/ Link to protocol |
|---|---|---|---|
| Orcel | 2013 | Group metacognitive training for adolescents with psychosis: Multiple case study. | Conference abstract; author emailed no response |
| Puskar | 2016 | A Case Study on Promoting Neuroplasticity in a Patient With Schizophrenia | Did not assess outcomes of interest |
| Ravishankar | CTRI/2019/ 02/017658 | To study the effectiveness of psychological therapy in patients with schizophrenia | Ongoing trial; http://ctri.nic.in/Clinicaltrials/pmaindet2.php ?trialid=29718&EncHid=&userName=CTRI /2019/02/017658 |
| Rek-Owodzin | 2019 | The effectiveness of metacognitive training for psychosis (MCT) in the ultra-high risk group (UHR) | Conference abstract; study discontinued |
| Rocha | 2013 | Metacognitive and social cognition training (MSCT) in schizophrenia: a preliminary efficacy study. | Mixed intervention; used a combination of metacognitive and social cognition training |
| Ross | 2010 | A Randomized Experimental Investigation of Reasoning Training for People With Delusions | Consists of a single session using two tasks from MCT and another exercise made by the authors |
| Schneider | DRKS0000 8001 | Investigating the efficacy of an individualized metacognitive therapy program (MCT plus) for psychosis: study protocol of a multi-center randomized controlled trial | Ongoing trial publication |
| Turner | 2019 | The Effect of Reducing the "Jumping to Conclusions" Bias on Treatment Decision-Making Capacity in Psychosis: a Randomized Controlled Trial With Mediation Analysis | Mixed intervention; one-session intervention based on MCT: "MCT-JTC" |
| Vitzthum | 2014 | Individualized Metacognitive Therapy Program for Patients with Psychosis (MCT+): Introduction of a Novel Approach for Psychotic Symptoms | Mixed intervention; patient completed MCT (group) and MCT+ concurrently |
| Woodward | NCT01764 568 2012 | Contrasting Group Therapy Methods for Psychosis | Ongoing trial; https://clinicaltrials.gov/ct2/show/study/NC T01764568 |
| Zonp | 2021 | The effectiveness of metacognitive training on impairments in social cognition in patients with schizophrenia: mental health nursing practice in a community mental health center | Did not assess outcomes of interest |

**eTable 8. Effect sizes of maintenance effectiveness by outcome.**

| Comparison | Outcome | Nb studies | Nb participants | Estimate (*g*) | Lower 95% CI | Upper 95% CI | *p*-value |
|---|---|---|---|---|---|---|---|
| Post-FUP<1y | Proximal | 14 | 832 | 0.03 | -0.09 | 0.14 | 0.66 |
| Post-FUP<1y | Positive sx | 14 | 832 | 0.08 | -0.03 | 0.19 | 0.17 |
| Post-FUP<1y | Delusions | 10 | 654 | 0.05 | -0.12 | 0.22 | 0.60 |
| Post-FUP<1y | Hallucinations | 2 | 202 | -0.08 | -0.56 | 0.40 | 0.74 |
| Post-FUP<1y | Cognitive bias | 10 | 658 | 0.06 | -0.12 | 0.24 | 0.51 |
| Post-FUP<1y | Distal | 11 | 631 | -0.01 | -0.15 | 0.14 | 0.94 |
| Post-FUP<1y | Negative sx | 6 | 342 | 0.01 | -0.20 | 0.22 | 0.95 |
| Post-FUP<1y | Self-esteem | 3 | 269 | -0.14 | -0.38 | 0.10 | 0.26 |
| Post-FUP<1y | Quality of life | 4 | 159 | 0.15 | -0.08 | 0.38 | 0.19 |
| Post-FUP<1y | Functioning | 4 | 205 | 0.16 | -0.06 | 0.37 | 0.15 |
| Post-FUP>1y | Proximal | 3 | 328 | 0.08 | -0.17 | 0.33 | 0.55 |
| Post-FUP>1y | Positive sx | 2 | 178 | 0.13 | -0.16 | 0.42 | 0.39 |
| Post-FUP>1y | Delusions | 1 | 76 | 0.03 | -0.29 | 0.34 | 0.87 |
| Post-FUP>1y | Hallucinations | 1 | 74 | 0.05 | -0.26 | 0.37 | 0.74 |
| Post-FUP>1y | Cognitive bias | 3 | 328 | -0.04 | -0.29 | 0.21 | 0.74 |
| Post-FUP>1y | Distal | 1 | 28 | 0.10 | -0.62 | 0.82 | 0.79 |
| Post-FUP>1y | Negative sx | 1 | 28 | 0.20 | -0.52 | 0.93 | 0.58 |
| Post-FUP>1y | Self-esteem | 0 | 0 | 0 | 0 | 0 | 0 |
| Post-FUP>1y | Quality of life | 1 | 28 | 0.12 | -0.60 | 0.84 | 0.75 |
| Post-FUP>1y | Functioning | 1 | 28 | 0.03 | -0.69 | 0.75 | 0.93 |
| Pre-FUP<1y | Proximal | 14 | 832 | 0.39 | 0.16 | 0.61 | 0.00 |
| Pre-FUP<1y | Positive sx | 14 | 832 | 0.49 | 0.22 | 0.76 | 0.00 |
| Pre-FUP<1y | Delusions | 10 | 654 | 0.61 | 0.16 | 1.06 | 0.01 |
| Pre-FUP<1y | Hallucinations | 2 | 202 | 0.07 | -0.21 | 0.34 | 0.62 |
| Pre-FUP<1y | Cognitive bias | 10 | 658 | 0.20 | -0.02 | 0.41 | 0.07 |
| Pre-FUP<1y | Distal | 11 | 631 | 0.30 | 0.14 | 0.46 | 0.00 |
| Pre-FUP<1y | Negative sx | 6 | 342 | 0.27 | 0.05 | 0.50 | 0.02 |
| Pre-FUP<1y | Self-esteem | 3 | 269 | 0.04 | -0.44 | 0.52 | 0.86 |
| Pre-FUP<1y | Quality of life | 4 | 159 | 0.15 | -0.23 | 0.52 | 0.44 |
| Pre-FUP<1y | Functioning | 4 | 205 | 0.53 | 0.30 | 0.75 | 0.00 |

*Note*. POST-FUP>1y = comparison of the follow-up (less than 1 year) to the post-intervention scores; Post-FUP<1y = comparison of the follow-up (more than 1 year) to the post-intervention scores; Pre-FUP<1y = comparison of the follow-up (less than 1 year) to the pre-intervention scores; *g* = Hedges' g; CI = Confidence intervals; sx = symptoms.

**eTable 9. Moderator and subgroup analyses of study, participant, and treatment characteristics on proximal and distal outcomes for the pre-post timepoints comparison.**

| Moderator | Proximal | | | | | | Distal | | | | | |
|---|---|---|---|---|---|---|---|---|---|---|---|---|
| | Nb studies | Statistic Type | Statistic Value | Lower 95% CI | Upper 95% CI | *p*-value | Nb studies | Statistic Type | Statistic Value | Lower 95% CI | Upper 95% CI | *p*-value |
| **Study characteristics** | | | | | | | | | | | | |
| *Publication year* | 38 | β | 0.00 | -0.04 | 0.03 | 0.85 | 26 | β | 0.00 | -0.04 | 0.03 | 0.91 |
| *MMAT assessment* [a] | | | | | | | | | | | | |
| 1 (lowest quality) | 2 | *g* | 0.43 | 0.03 | 0.82 | 0.03 | 2 | *g* | 0.13 | -0.12 | 0.38 | 0.29 |
| 2 | 10 | *g* | 0.41 | 0.25 | 0.57 | 0.00 | 6 | *g* | 0.45 | 0.17 | 0.72 | 0.00 |
| 3 | 6 | *g* | 0.18 | -0.11 | 0.47 | 0.23 | 5 | *g* | 0.03 | -0.20 | 0.26 | 0.80 |
| 4 | 11 | *g* | 0.47 | 0.12 | 0.83 | 0.01 | 7 | *g* | 0.31 | 0.13 | 0.50 | 0.00 |
| 5 (highest quality) | 9 | *g* | 0.48 | 0.15 | 0.81 | 0.00 | 6 | *g* | 0.52 | 0.19 | 0.85 | 0.00 |
| Subgroup differences | | $Q_4$ | 2.57 | | | 0.63 | | $Q_4$ | 9.33 | | | 0.05 |
| *Type of analysis* | | | | | | | | | | | | |
| N/R | 11 | *g* | 0.28 | 0.11 | 0.44 | 0.00 | 0 | *g* | 0.00 | 0.00 | 0.00 | 0.00 |
| Intention-to-treat | 9 | *g* | 0.37 | 0.09 | 0.65 | 0.01 | 4 | *g* | 0.33 | 0.09 | 0.57 | 0.01 |
| Per-protocol | 18 | *g* | 0.53 | 0.30 | 0.76 | 0.00 | 15 | *g* | 0.33 | 0.16 | 0.50 | 0.00 |
| Subgroup differences | | $Q_2$ | 3.03 | | | 0.22 | | $Q_2$ | 0.12 | | | 0.94 |
| *Type of control condition* | | | | | | | | | | | | |
| Active | 11 | *g* | 0.28 | -0.02 | 0.58 | 0.06 | 8 | *g* | 0.11 | -0.07 | 0.29 | 0.24 |
| None | 6 | *g* | 0.34 | 0.16 | 0.52 | 0.00 | 5 | *g* | 0.35 | 0.19 | 0.52 | 0.00 |
| Other | 2 | *g* | 0.45 | 0.16 | 0.75 | 0.00 | 2 | *g* | 0.59 | -0.08 | 1.26 | 0.09 |
| Passive | 19 | *g* | 0.48 | 0.22 | 0.73 | 0.00 | 11 | *g* | 0.43 | 0.16 | 0.70 | 0.00 |
| Subgroup differences | | $Q_3$ | 1.08 | | | 0.78 | | $Q_3$ | 6.36 | | | 0.10 |
| *Type of design* | | | | | | | | | | | | |
| Non-randomized | 11 | *g* | 0.46 | 0.18 | 0.75 | 0.00 | 7 | *g* | 0.46 | 0.23 | 0.68 | 0.00 |
| Randomized controlled | 26 | *g* | 0.37 | 0.20 | 0.53 | 0.00 | 19 | *g* | 0.25 | 0.11 | 0.39 | 0.00 |
| Subgroup differences | | $Q_1$ | 0.34 | | | 0.56 | | $Q_1$ | 2.27 | | | 0.13 |
| **Treatment characteristics** | | | | | | | | | | | | |
| *Nb MCT sessions* | | | | | | | | | | | | |
| Less than 8 | 6 | *g* | 0.68 | 0.35 | 1.01 | 0.00 | 3 | *g* | 0.73 | 0.04 | 1.42 | 0.04 |
| One cycle (8) | 17 | *g* | 0.39 | 0.13 | 0.66 | 0.00 | 11 | *g* | 0.24 | 0.09 | 0.38 | 0.00 |
| Two cycles (16) | 6 | *g* | 0.22 | 0.06 | 0.37 | 0.01 | 5 | *g* | 0.41 | 0.14 | 0.69 | 0.00 |
| More than 16 | 3 | *g* | 0.30 | -0.05 | 0.64 | 0.09 | 2 | *g* | 0.46 | -0.62 | 1.54 | 0.40 |
| Other | 6 | *g* | 0.37 | 0.17 | 0.57 | 0.00 | 0 | *g* | 0.00 | 0.00 | 0.00 | 0.00 |
| Subgroup differences | | $Q_4$ | 6.63 | | | 0.16 | | $Q_4$ | 3.58 | | | 0.47 |

| Moderator | Proximal | | | | | | Distal | | | | | |
|---|---|---|---|---|---|---|---|---|---|---|---|---|
| | Nb studies | Statistic Type | Statistic Value | Lower 95% CI | Upper 95% CI | *p*-value | Nb studies | Statistic Type | Statistic Value | Lower 95% CI | Upper 95% CI | *p*-value |
| *Delivery format* | | | | | | | | | | | | |
| N/R | 2 | *g* | 1.35 | -0.48 | 3.19 | 0.15 | 1 | *g* | 0.97 | 0.43 | 1.50 | 0.00 |
| Both | 1 | *g* | 0.38 | -0.18 | 0.94 | 0.19 | 0 | *g* | 0.00 | 0.00 | 0.00 | 0.00 |
| Individual | 5 | *g* | 0.79 | 0.40 | 1.18 | 0.00 | 3 | *g* | 0.43 | -0.32 | 1.17 | 0.26 |
| Group | 30 | *g* | 0.29 | 0.17 | 0.41 | 0.00 | 22 | *g* | 0.29 | 0.18 | 0.41 | 0.00 |
| Subgroup differences | | $Q_3$ | 7.07 | | | 0.07 | | $Q_3$ | 5.86 | | | 0.05 |
| *Facilitators credentials* | | | | | | | | | | | | |
| N/R | 10 | *g* | 0.45 | 0.29 | 0.61 | 0.00 | 0 | *g* | 0.00 | 0.00 | 0.00 | 0.00 |
| GP | 1 | *g* | 0.46 | 0.10 | 0.82 | 0.01 | 1 | *g* | 0.28 | -0.08 | 0.63 | 0.13 |
| Graduate student | 1 | *g* | 0.31 | -0.27 | 0.90 | 0.30 | 0 | *g* | 0.00 | 0.00 | 0.00 | 0.00 |
| Nurse | 5 | *g* | 0.78 | 0.18 | 1.38 | 0.01 | 2 | *g* | 0.51 | 0.11 | 0.91 | 0.01 |
| Occupational therapist | 2 | *g* | 0.42 | 0.11 | 0.73 | 0.01 | 2 | *g* | 0.17 | -0.13 | 0.47 | 0.27 |
| Psychiatrist | 4 | *g* | 0.54 | -0.03 | 1.11 | 0.06 | 4 | *g* | 0.43 | -0.01 | 0.87 | 0.06 |
| Psychologist | 15 | *g* | 0.21 | 0.00 | 0.42 | 0.06 | 9 | *g* | 0.21 | 0.00 | 0.43 | 0.05 |
| Subgroup differences | | $Q_6$ | 5.83 | | | 0.44 | | $Q_5$ | 3.32 | | | 0.65 |
| *Training of facilitators* | | | | | | | | | | | | |
| No or N/R | 16 | *g* | 0.50 | 0.29 | 0.71 | 0.00 | 10 | *g* | 0.43 | 0.21 | 0.66 | 0.00 |
| Yes | 22 | *g* | 0.31 | 0.13 | 0.49 | 0.00 | 16 | *g* | 0.25 | 0.11 | 0.40 | 0.00 |
| Subgroup differences | | $Q_1$ | 1.86 | | | 0.17 | | $Q_1$ | 1.72 | | | 0.19 |
| *Adherence to MCT manual* | | | | | | | | | | | | |
| No or N/R | 14 | *g* | 0.50 | 0.26 | 0.75 | 0.00 | 9 | *g* | 0.48 | 0.21 | 0.75 | 0.00 |
| Yes | 24 | *g* | 0.33 | 0.17 | 0.49 | 0.00 | 17 | *g* | 0.25 | 0.12 | 0.39 | 0.00 |
| Subgroup differences | | $Q_1$ | 1.30 | | | 0.25 | | $Q_1$ | 2.15 | | | 0.14 |
| **Participant characteristics** | | | | | | | | | | | | |
| *Age* (years) | 36 | β | 0.00 | -0.07 | 0.02 | 0.84 | 25 | β | 0.01 | -0.01 | 0.02 | 0.39 |
| *Duration of illness* (years) | 20 | β | 0.01 | -0.01 | 0.02 | 0.44 | 17 | β | 0.01 | -0.01 | 0.03 | 0.31 |
| *Sex* (%male) | 36 | β | -0.01 | -0.02 | 0.00 | 0.18 | 26 | β | 0.00 | -0.01 | 0.01 | 0.82 |
| *Medication* (chlorpromazine equivalents) | 17 | β | 0.00 | 0.00 | 0.00 | 0.91 | 12 | β | 0.00 | 0.00 | 0.00 | 0.68 |
| *Diagnosis (%SSD)* | 35 | β | 0.01 | -0.01 | 0.02 | 0.35 | 24 | β | -0.02 | -0.04 | 0.00 | 0.11 |

*Note*. a = Number of "yes"; β = Beta coefficient for metaregression; *g* = Hedges' g; GP = general practitioner; MMAT = Mixed methods appraisal tool; N/R = not reported; Q = Q-statistic for between-group comparison; SSD = Schizophrenia-spectrum disorders; gender (i.e., including non-binary, other, etc. categories) was not included as a moderator as it was not assessed by any study.

**eTable 10. Moderator and subgroup analyses of study, participant, and treatment characteristics on separate outcomes for the pre-post timepoints comparison.**

| Moderator | Positive symptoms[b] | | | | | | Delusions[b] | | | | | |
|---|---|---|---|---|---|---|---|---|---|---|---|---|
| | Nb studies | Statistic Type | Statistic Value | Lower 95% CI | Upper 95% CI | *p*-value | Nb studies | Statistic Type | Statistic Value | Lower 95% CI | Upper 95% CI | *p*-value |
| **Study characteristics** | | | | | | | | | | | | |
| *Publication year* | 36 | β | -0.01 | -0.06 | 0.03 | 0.62 | 23 | β | -0.02 | -0.09 | 0.05 | 0.64 |
| *MMAT assessment* [a] | | | | | | | | | | | | |
| 1 (lowest quality) | 2 | *g* | 0.44 | 0.01 | 0.86 | 0.05 | 2 | *g* | 0.37 | -0.22 | 0.97 | 0.22 |
| 2 | 9 | *g* | 0.62 | 0.28 | 0.96 | 0.00 | 5 | *g* | 0.96 | 0.20 | 1.71 | 0.01 |
| 3 | 6 | *g* | 0.22 | -0.16 | 0.61 | 0.25 | 2 | *g* | 0.58 | -0.21 | 1.38 | 0.15 |
| 4 | 11 | *g* | 0.53 | 0.15 | 0.91 | 0.01 | 8 | *g* | 0.72 | 0.26 | 1.18 | 0.00 |
| 5 (highest quality) | 8 | *g* | 0.58 | 0.24 | 0.91 | 0.00 | 6 | *g* | 0.63 | 0.34 | 0.93 | 0.00 |
| Subgroup differences | | $Q_4$ | 2.73 | | | 0.60 | | $Q_4$ | 1.57 | | | 0.81 |
| *Type of analysis* | | | | | | | | | | | | |
| N/R | 10 | *g* | 0.33 | 0.16 | 0.49 | 0.00 | 6 | *g* | 0.45 | 0.24 | 0.66 | 0.00 |
| Intention-to-treat | 8 | *g* | 0.66 | 0.23 | 1.09 | 0.00 | 7 | *g* | 0.83 | 0.23 | 1.43 | 0.01 |
| Per-protocol | 18 | *g* | 0.58 | 0.33 | 0.83 | 0.00 | 10 | *g* | 0.80 | 0.44 | 1.15 | 0.00 |
| Subgroup differences | | $Q_2$ | 3.98 | | | 0.14 | | $Q_2$ | 3.50 | | | 0.17 |
| *Type of control condition* | | | | | | | | | | | | |
| Active | 11 | *g* | 0.30 | 0.00 | 0.61 | 0.05 | 6 | *g* | 0.48 | 0.14 | 0.82 | 0.01 |
| None | 6 | *g* | 0.47 | 0.24 | 0.70 | 0.00 | 4 | *g* | 0.66 | 0.34 | 0.98 | 0.00 |
| Other | 1 | *g* | 0.43 | -0.08 | 0.94 | 0.10 | 0 | *g* | 0 | 0 | 0 | 0 |
| Passive | 18 | *g* | 0.65 | 0.34 | 0.96 | 0.00 | 13 | *g* | 0.81 | 0.36 | 1.27 | 0.00 |
| Subgroup differences | | $Q_3$ | 2.44 | | | 0.49 | | $Q_3$ | 1.39 | | | 0.50 |
| *Type of design* | | | | | | | | | | | | |
| Non-randomized | 10 | *g* | 0.61 | 0.29 | 0.93 | 0.00 | 7 | *g* | 0.90 | 0.51 | 1.29 | 0.00 |
| Randomized controlled | 24 | *g* | 0.47 | 0.25 | 0.69 | 0.00 | 15 | *g* | 0.64 | 0.32 | 0.96 | 0.00 |
| Subgroup differences | | $Q_1$ | 0.48 | | | 0.49 | | $Q_1$ | 1.03 | | | 0.31 |
| **Treatment characteristics** | | | | | | | | | | | | |
| *Nb MCT sessions* | | | | | | | | | | | | |
| Less than 8 | 6 | *g* | 0.97 | 0.42 | 1.52 | 0.00 | 6 | *g* | 1.14 | 0.50 | 1.77 | 0.00 |
| One cycle (8) | 15 | *g* | 0.48 | 0.18 | 0.78 | 0.00 | 10 | *g* | 0.63 | 0.27 | 0.98 | 0.00 |
| Two cycles (16) | 6 | *g* | 0.22 | 0.06 | 0.37 | 0.01 | 2 | *g* | 0.38 | -0.08 | 0.84 | 0.10 |
| More than 16 | 3 | *g* | 0.30 | -0.05 | 0.64 | 0.09 | 1 | *g* | 0.42 | -0.14 | 0.99 | 0.14 |
| Other | 6 | *g* | 0.48 | 0.28 | 0.68 | 0.00 | 4 | *g* | 0.45 | 0.00 | 0.89 | 0.05 |
| Subgroup differences | | $Q_4$ | 10.03 | | | 0.04 | | $Q_4$ | 4.41 | | | 0.35 |

| Moderator | Positive symptoms[b] | | | | | | Delusions[b] | | | | | |
|---|---|---|---|---|---|---|---|---|---|---|---|---|
| | Nb studies | Statistic Type | Statistic Value | Lower 95% CI | Upper 95% CI | *p*-value | Nb studies | Statistic Type | Statistic Value | Lower 95% CI | Upper 95% CI | *p*-value |
| *Delivery format* | | | | | | | | | | | | |
| N/R | 2 | *g* | 1.35 | -0.48 | 3.19 | 0.15 | 1 | *g* | 2.30 | 1.61 | 3.00 | 0.00 |
| Both | 1 | *g* | 0.38 | -0.18 | 0.94 | 0.19 | 1 | *g* | 0.42 | -0.14 | 0.99 | 0.14 |
| Group | 28 | *g* | 0.35 | 0.21 | 0.50 | 0.00 | 16 | *g* | 0.46 | 0.27 | 0.66 | 0.00 |
| Individual | 5 | *g* | 1.13 | 0.57 | 1.68 | 0.00 | 5 | *g* | 1.25 | 0.51 | 1.99 | 0.00 |
| Subgroup differences | | $Q_3$ | 7.90 | | | 0.05 | | $Q_3$ | 28.26 | | | 0.00 |
| *Facilitators credentials* | | | | | | | | | | | | |
| N/R | 10 | *g* | 0.50 | 0.28 | 0.72 | 0.00 | 5 | *g* | 0.64 | 0.36 | 0.92 | 0.00 |
| GP | 1 | *g* | 0.46 | 0.10 | 0.82 | 0.01 | 1 | *g* | 0.43 | 0.07 | 0.79 | 0.02 |
| Graduate student | 1 | *g* | 0.23 | -0.36 | 0.81 | 0.44 | 1 | *g* | 0.22 | -0.37 | 0.80 | 0.47 |
| Nurse | 4 | *g* | 1.00 | 0.35 | 1.64 | 0.00 | 3 | *g* | 1.45 | 0.72 | 2.18 | 0.00 |
| Occupational therapist | 2 | *g* | 0.43 | 0.12 | 0.74 | 0.01 | 1 | *g* | 0.55 | 0.00 | 1.11 | 0.05 |
| Psychiatrist | 4 | *g* | 0.68 | 0.07 | 1.28 | 0.03 | 1 | *g* | 0.39 | -0.17 | 0.94 | 0.17 |
| Psychologist | 14 | *g* | 0.37 | 0.08 | 0.66 | 0.01 | 11 | *g* | 0.64 | 0.21 | 1.06 | 0.00 |
| Subgroup differences | | $Q_6$ | 4.27 | | | 0.64 | | $Q_5$ | 8.29 | | | 0.22 |
| *Training of facilitators* | | | | | | | | | | | | |
| No or N/R | 15 | *g* | 0.62 | 0.40 | 0.84 | 0.00 | 9 | *g* | 0.81 | 0.49 | 1.12 | 0.00 |
| Yes | 21 | *g* | 0.41 | 0.19 | 0.64 | 0.00 | 14 | *g* | 0.61 | 0.27 | 0.96 | 0.00 |
| Subgroup differences | | $Q_1$ | 1.61 | | | 0.20 | | $Q_1$ | 0.64 | | | 0.42 |
| *Adherence to MCT manual* | | | | | | | | | | | | |
| No or N/R | 14 | *g* | 0.55 | 0.29 | 0.81 | 0.00 | 8 | *g* | 0.80 | 0.42 | 1.19 | 0.00 |
| Yes | 22 | *g* | 0.48 | 0.26 | 0.69 | 0.00 | 15 | *g* | 0.63 | 0.31 | 0.94 | 0.00 |
| Subgroup differences | | $Q_1$ | 0.17 | | | 0.68 | | $Q_1$ | 0.47 | | | 0.49 |
| **Participant characteristics** | | | | | | | | | | | | |
| *Age* (years) | 34 | β | -0.01 | -0.04 | 0.01 | 0.26 | 22 | β | -0.02 | -0.05 | 0.02 | 0.44 |
| *Duration of illness* (years) | 19 | β | 0.00 | -0.01 | 0.02 | 0.74 | 11 | β | 0.00 | -0.03 | 0.04 | 0.85 |
| *Sex* (%male) | 34 | β | -0.01 | -0.02 | 0.00 | 0.20 | 21 | β | -0.01 | -0.03 | 0.01 | 0.22 |
| *Medication* (chlorpromazine equivalents) | 17 | β | 0.00 | 0.00 | 0.00 | 0.74 | 11 | β | 0.00 | 0.00 | 0.00 | 0.37 |
| *Diagnosis (%SSD)* | 32 | β | -0.01 | -0.02 | 0.01 | 0.34 | 21 | β | -0.01 | -0.03 | 0.01 | 0.19 |

*Note*. a = Number of "yes"; b = Results must be interpreted cautiously due to the low number of studies per subgroup for some analyses; β = Beta coefficient for metaregression; *g* = Hedges' g; GP = general practitioner N/R = not reported; Q = Q-statistic for between-group comparison; SSD = Schizophrenia-spectrum disorder; gender (i.e., including non-binary, other, etc. categories) was not included as a moderator as it was not assessed by any study.

| Moderator | Hallucinations[b] | | | | | | Cognitive bias[b] | | | | | |
|---|---|---|---|---|---|---|---|---|---|---|---|---|
| | Nb studies | Statistic Type | Statistic Value | Lower 95% CI | Upper 95% CI | *p*-value | Nb studies | Statistic Type | Statistic Value | Lower 95% CI | Upper 95% CI | *p*-value |
| **Study characteristics** | | | | | | | | | | | | |
| *Publication year* | 9 | β | 0.04 | 0.00 | 0.07 | 0.03 | 19 | β | 0.00 | -0.03 | 0.04 | 0.88 |
| *MMAT assessment* [a] | | | | | | | | | | | | |
| 1 (lowest quality) | 0 | *g* | 0.00 | 0.00 | 0.00 | 0.00 | 1 | *g* | 0.48 | 0.19 | 0.77 | 0.00 |
| 2 | 1 | *g* | 0.30 | -0.25 | 0.85 | 0.28 | 6 | *g* | 0.27 | 0.08 | 0.46 | 0.00 |
| 3 | 2 | *g* | 0.28 | 0.02 | 0.54 | 0.04 | 4 | *g* | -0.03 | -0.33 | 0.27 | 0.84 |
| 4 | 3 | *g* | 0.22 | -0.18 | 0.61 | 0.28 | 5 | *g* | 0.13 | -0.16 | 0.41 | 0.38 |
| 5 (highest quality) | 3 | *g* | 0.35 | 0.00 | 0.70 | 0.05 | 3 | *g* | -0.01 | -0.53 | 0.51 | 0.98 |
| Subgroup differences | | $Q_3$ | 0.24 | | | 0.97 | | $Q_4$ | 7.25 | | | 0.12 |
| *Type of analysis* | | | | | | | | | | | | |
| N/R | 2 | *g* | 0.24 | -0.24 | 0.71 | 0.33 | 5 | *g* | 0.24 | -0.07 | 0.55 | 0.14 |
| Intention-to-treat | 3 | *g* | 0.17 | -0.19 | 0.53 | 0.36 | 7 | *g* | 0.13 | -0.04 | 0.30 | 0.13 |
| Per-protocol | 4 | *g* | 0.35 | 0.15 | 0.55 | 0.00 | 7 | *g* | 0.14 | -0.10 | 0.38 | 0.27 |
| Subgroup differences | | $Q_2$ | 0.82 | | | 0.66 | | $Q_2$ | 0.34 | | | 0.84 |
| *Type of control condition* | | | | | | | | | | | | |
| Active | 4 | *g* | 0.23 | -0.07 | 0.53 | 0.14 | 6 | *g* | 0.13 | -0.20 | 0.45 | 0.45 |
| None | 2 | *g* | 0.28 | 0.02 | 0.54 | 0.04 | 4 | *g* | 0.21 | -0.05 | 0.47 | 0.11 |
| Other | 0 | *g* | 0 | 0 | 0 | 0 | 0 | *g* | 0 | 0 | 0 | 0 |
| Passive | 3 | *g* | 0.35 | 0.00 | 0.71 | 0.05 | 9 | *g* | 0.14 | -0.03 | 0.31 | 0.10 |
| Subgroup differences | | $Q_2$ | 0.29 | | | 0.86 | | $Q_2$ | 0.24 | | | 0.89 |
| *Type of design* | | | | | | | | | | | | |
| Non-randomized | 2 | *g* | 0.18 | -0.14 | 0.50 | 0.27 | 5 | *g* | 0.19 | 0.00 | 0.43 | 0.14 |
| Randomized controlled | 6 | *g* | 0.26 | 0.00 | 0.48 | 0.00 | 14 | *g* | 0.15 | 0.00 | 0.31 | 0.00 |
| Subgroup differences | | $Q_1$ | 0.18 | | | 0.67 | | $Q_1$ | 0.00 | | | 0.84 |
| **Treatment characteristics** | | | | | | | | | | | | |
| *Nb MCT sessions* | | | | | | | | | | | | |
| Less than 8 | 0 | *g* | 0 | 0 | 0 | 0 | 5 | *g* | 0.36 | 0.13 | 0.59 | 0.00 |
| One cycle (8) | 4 | *g* | 0.27 | -0.05 | 0.60 | 0.10 | 10 | *g* | 0.04 | -0.15 | 0.23 | 0.65 |
| Two cycles (16) | 2 | *g* | 0.27 | -0.19 | 0.72 | 0.25 | 0 | *g* | 0 | 0 | 0 | 0 |
| More than 16 | 1 | *g* | 0.25 | -0.31 | 0.80 | 0.39 | 0 | *g* | 0 | 0 | 0 | 0 |
| Other | 2 | *g* | 0.28 | 0.02 | 0.54 | 0.04 | 4 | *g* | 0.22 | 0.00 | 0.45 | 0.05 |
| Subgroup differences | | $Q_3$ | 0.01 | | | 1.00 | | $Q_4$ | 4.54 | | | 0.10 |
| *Delivery format* | | | | | | | | | | | | |
| N/R | 0 | *g* | 0 | 0 | 0 | 0 | 0 | *g* | 0 | 0 | 0 | 0 |
| Both | 1 | *g* | 0.25 | -0.31 | 0.80 | 0.39 | 0 | *g* | 0 | 0 | 0 | 0 |
| Individual | 8 | *g* | 0.26 | 0.10 | 0.42 | 0.00 | 16 | *g* | 0.13 | -0.01 | 0.26 | 0.06 |
| Group | 0 | *g* | 0 | 0 | 0 | 0 | 3 | *g* | 0.42 | 0.03 | 0.80 | 0.04 |

| Moderator | Hallucinations[b] | | | | | | Cognitive bias[b] | | | | | |
|---|---|---|---|---|---|---|---|---|---|---|---|---|
| | Nb studies | Statistic Type | Statistic Value | Lower 95% CI | Upper 95% CI | *p*-value | Nb studies | Statistic Type | Statistic Value | Lower 95% CI | Upper 95% CI | *p*-value |
| Subgroup differences | | $Q_1$ | 0.00 | | | 0.96 | | $Q_1$ | 1.92 | | | 0.17 |
| *Facilitators credentials* | | | | | | | | | | | | |
| N/R | 0 | *g* | 0.00 | 0.00 | 0.00 | 0.00 | 3 | *g* | 0.29 | -0.03 | 0.61 | 0.08 |
| GP | 1 | *g* | 0.50 | 0.14 | 0.87 | 0.01 | 0 | *g* | 0 | 0 | 0 | 0 |
| Graduate student | 1 | *g* | 0.26 | -0.33 | 0.84 | 0.39 | 1 | *g* | 0.38 | -0.21 | 0.97 | 0.20 |
| Nurse | 2 | *g* | 0.28 | -0.03 | 0.58 | 0.07 | 2 | *g* | 0.23 | -0.11 | 0.57 | 0.19 |
| Occupational therapist | 1 | *g* | 0.30 | -0.25 | 0.85 | 0.28 | 1 | *g* | 0.39 | -0.17 | 0.94 | 0.17 |
| Psychiatrist | 0 | *g* | 0 | 0 | 0 | 0 | 1 | *g* | 0.19 | -0.38 | 0.76 | 0.51 |
| Psychologist | 4 | *g* | 0.16 | -0.10 | 0.42 | 0.23 | 11 | *g* | 0.05 | -0.14 | 0.24 | 0.60 |
| Subgroup differences | | $Q_4$ | 2.29 | | | 0.68 | | $Q_5$ | 3.31 | | | 0.65 |
| *Training of facilitators* | | | | | | | | | | | | |
| No or N/R | 2 | *g* | 0.19 | -0.10 | 0.49 | 0.19 | 6 | *g* | 0.22 | -0.01 | 0.45 | 0.06 |
| Yes | 6 | *g* | 0.26 | 0.03 | 0.49 | 0.03 | 13 | *g* | 0.13 | -0.03 | 0.29 | 0.11 |
| Subgroup differences | | $Q_1$ | 0.26 | | | 0.61 | | $Q_1$ | 0.37 | | | 0.54 |
| *Adherence to MCT manual* | | | | | | | | | | | | |
| No or N/R | 2 | *g* | 0.19 | -0.10 | 0.49 | 0.19 | 3 | *g* | 0.20 | -0.01 | 0.42 | 0.06 |
| Yes | 7 | *g* | 0.29 | 0.09 | 0.48 | 0.00 | 16 | *g* | 0.14 | -0.01 | 0.30 | 0.07 |
| Subgroup differences | | $Q_1$ | 0.26 | | | 0.61 | | $Q_1$ | 0.19 | | | 0.67 |
| **Participant characteristics** | | | | | | | | | | | | |
| *Age* (years) | 9 | β | 0.01 | -0.01 | 0.03 | 0.20 | 18 | β | 0.01 | -0.01 | 0.03 | 0.25 |
| *Duration of illness* (years) | 6 | β | 0.00 | -0.02 | 0.03 | 0.77 | 8 | β | 0.01 | -0.01 | 0.04 | 0.31 |
| *Sex* (%male) | 8 | β | -0.02 | -0.03 | 0.00 | 0.08 | 18 | β | -0.01 | -0.02 | 0.00 | 0.23 |
| *Medication* (chlorpromazine equivalents) | 0 | β | 0 | 0 | 0 | 0 | 13 | β | 0.00 | 0.00 | 0.00 | 1.00 |
| *Diagnosis (%SSD)* | 9 | β | 0 | -0.03 | 0.02 | 0.79 | 18 | β | 0.00 | -0.01 | 0.01 | 0.59 |

*Note.* a = Number of "yes"; b = Results must be interpreted cautiously because of the low number of studies per subgroup for some analyses; β = Beta coefficient for metaregression; *g* = Hedges' g; GP = general practitioner N/R = not reported; Q = Q-statistic for between-group comparison; SSD = Schizophrenia-Spectrum Disorder; gender (i.e., including non-binary, other, etc. categories) was not included as a moderator as it was not assessed by any study.

| Moderator | Negative symptoms[b] | | | | | | Self-esteem[b] | | | | | |
|---|---|---|---|---|---|---|---|---|---|---|---|---|
| | Nb studies | Statistic Type | Statistic Value | Lower 95% CI | Upper 95% CI | *p*-value | Nb studies | Statistic Type | Statistic Value | Lower 95% CI | Upper 95% CI | *p*-value |
| **Study characteristics** | | | | | | | | | | | | |
| *Publication year* | 17 | β | 0.01 | -0.04 | 0.07 | 0.59 | 5 | β | -0.01 | -0.06 | 0.03 | 0.52 |
| *MMAT assessment* [a] | | | | | | | | | | | | |
| 1 (lowest quality) | 1 | *g* | 0.01 | -0.56 | 0.59 | 0.96 | 2 | *g* | 0.14 | -0.11 | 0.39 | 0.26 |
| 2 | 4 | *g* | 0.48 | 0.20 | 0.77 | 0.00 | 0 | *g* | 0 | 0 | 0 | 0 |
| 3 | 5 | *g* | 0.01 | -0.21 | 0.23 | 0.92 | 0 | *g* | 0 | 0 | 0 | 0 |
| 4 | 5 | *g* | 0.36 | 0.16 | 0.55 | 0.00 | 1 | *g* | 0.21 | -0.11 | 0.53 | 0.19 |
| 5 (highest quality) | 2 | *g* | 0.20 | -0.34 | 0.73 | 0.47 | 2 | *g* | 0.12 | -0.20 | 0.44 | 0.46 |
| Subgroup differences | | $Q_4$ | 8.85 | | | 0.06 | | $Q_4$ | 0.19 | | | 0.91 |
| *Type of analysis* | | | | | | | | | | | | |
| N/R | 5 | *g* | 0.35 | -0.01 | 0.71 | 0.06 | 2 | *g* | 0.14 | -0.11 | 0.39 | 0.26 |
| Intention-to-treat | 0 | *g* | 0 | 0 | 0 | 0 | 2 | *g* | 0.10 | -0.25 | 0.44 | 0.57 |
| Per-protocol | 12 | *g* | 0.19 | 0.05 | 0.32 | 0.01 | 1 | *g* | 0.21 | 0.01 | 0.41 | 0.04 |
| Subgroup differences | | $Q_2$ | 0.68 | | | 0.41 | | $Q_2$ | 0.36 | | | 0.83 |
| *Type of control condition* | | | | | | | | | | | | |
| Active | 6 | *g* | 0.12 | -0.08 | 0.32 | 0.24 | 1 | *g* | 0.21 | -0.11 | 0.53 | 0.19 |
| None | 3 | *g* | 0.16 | -0.12 | 0.44 | 0.27 | 2 | *g* | 0.20 | 0.04 | 0.36 | 0.02 |
| Other | 1 | *g* | 0.59 | 0.07 | 1.10 | 0.03 | 0 | *g* | 0 | 0 | 0 | 0 |
| Passive | 7 | *g* | 0.37 | 0.15 | 0.59 | 0.00 | 2 | *g* | -0.08 | -0.48 | 0.33 | 0.71 |
| Subgroup differences | | $Q_3$ | 4.65 | | | 0.20 | | $Q_2$ | 1.61 | | | 0.45 |
| *Type of design* | | | | | | | | | | | | |
| Non-randomized | 3 | *g* | 0.30 | -0.13 | 0.73 | 0.17 | 2 | *g* | 0.20 | 0.00 | 0.36 | 0.00 |
| Randomized controlled | 13 | *g* | 0.25 | 0.00 | 0.40 | 0.00 | 3 | *g* | 0.10 | -0.15 | 0.35 | 0.42 |
| Subgroup differences | | $Q_1$ | 0.00 | | | 0.82 | | $Q_1$ | 0.39 | | | 0.53 |
| **Treatment characteristics** | | | | | | | | | | | | |
| *Nb MCT sessions* | | | | | | | | | | | | |
| Less than 8 | 2 | *g* | 0.49 | -0.25 | 1.22 | 0.19 | 0 | *g* | 0 | 0 | 0 | 0 |
| One cycle (8) | 7 | *g* | 0.22 | 0.02 | 0.41 | 0.03 | 2 | *g* | 0.19 | -0.02 | 0.40 | 0.07 |
| Two cycles (16) | 4 | *g* | 0.22 | -0.07 | 0.50 | 0.14 | 1 | *g* | 0.21 | 0.01 | 0.41 | 0.04 |
| More than 16 | 1 | *g* | 0.59 | 0.07 | 1.10 | 0.03 | 0 | *g* | 0 | 0 | 0 | 0 |
| Other | 3 | *g* | 0.10 | -0.18 | 0.38 | 0.50 | 2 | *g* | -0.08 | -0.48 | 0.33 | 0.71 |
| Subgroup differences | | $Q_4$ | 3.21 | | | 0.52 | | $Q_2$ | 1.62 | | | 0.45 |
| *Delivery format* | | | | | | | | | | | | |
| N/R | 1 | *g* | 0.59 | 0.07 | 1.10 | 0.03 | 0 | *g* | 0 | 0 | 0 | 0 |
| Both | 0 | *g* | 0 | 0 | 0 | 0 | 0 | *g* | 0 | 0 | 0 | 0 |
| Individual | 14 | *g* | 0.23 | 0.08 | 0.38 | 0.00 | 4 | *g* | 0.18 | 0.04 | 0.32 | 0.01 |
| Group | 2 | *g* | 0.07 | -0.32 | 0.46 | 0.73 | 1 | *g* | 0.02 | -0.56 | 0.59 | 0.96 |

| Moderator | Negative symptoms[b] | | | | | | Self-esteem[b] | | | | | |
|---|---|---|---|---|---|---|---|---|---|---|---|---|
| | Nb studies | Statistic Type | Statistic Value | Lower 95% CI | Upper 95% CI | *p*-value | Nb studies | Statistic Type | Statistic Value | Lower 95% CI | Upper 95% CI | *p*-value |
| Subgroup differences | | $Q_3$ | 2.51 | | | 0.28 | | $Q_1$ | 0.29 | | | 0.59 |
| *Facilitators credentials* | | | | | | | | | | | | |
| N/R | 5 | *g* | 0.39 | 0.17 | 0.61 | 0.00 | 1 | *g* | 0.17 | -0.10 | 0.45 | 0.22 |
| GP | 1 | *g* | 0.27 | -0.09 | 0.62 | 0.14 | 0 | *g* | 0 | 0 | 0 | 0 |
| Graduate student | 0 | *g* | 0 | 0 | 0 | 0 | 0 | *g* | 0 | 0 | 0 | 0 |
| Nurse | 0 | *g* | 0 | 0 | 0 | 0 | 0 | *g* | 0 | 0 | 0 | 0 |
| Occupational therapist | 1 | *g* | 0.27 | -0.09 | 0.63 | 0.14 | 1 | *g* | -0.17 | -0.75 | 0.40 | 0.56 |
| Psychiatrist | 3 | *g* | 0.46 | 0.07 | 0.84 | 0.02 | 0 | *g* | 0 | 0 | 0 | 0 |
| Psychologist | 7 | *g* | 0.10 | -0.11 | 0.30 | 0.35 | 3 | *g* | 0.20 | 0.03 | 0.36 | 0.02 |
| Subgroup differences | | $Q_6$ | 4.72 | | | 0.32 | | $Q_5$ | 1.44 | | | 0.49 |
| *Training of facilitators* | | | | | | | | | | | | |
| No or N/R | 8 | *g* | 0.39 | 0.22 | 0.56 | 0.00 | 1 | *g* | 0.17 | -0.10 | 0.45 | 0.22 |
| Yes | 9 | *g* | 0.08 | -0.05 | 0.21 | 0.23 | 4 | *g* | 0.17 | 0.01 | 0.33 | 0.03 |
| Subgroup differences | | $Q_1$ | 8.17 | | | 0.00 | | $Q_1$ | 0.00 | | | 0.98 |
| *Adherence to MCT manual* | | | | | | | | | | | | |
| No or N/R | 6 | *g* | 0.37 | 0.18 | 0.57 | 0.00 | 0 | *g* | 0 | 0 | 0 | 0 |
| Yes | 11 | *g* | 0.16 | 0.01 | 0.32 | 0.04 | 5 | *g* | 0.17 | 0.03 | 0.31 | 0.01 |
| Subgroup differences | | $Q_1$ | 2.67 | | | 0.10 | | $Q_1$ | 0.00 | | | 1.00 |
| **Participant characteristics** | | | | | | | | | | | | |
| *Age* (years) | 16 | β | 0.01 | -0.01 | 0.02 | 0.34 | 4 | β | -0.01 | -0.05 | 0.03 | 0.67 |
| *Duration of illness* (years) | 11 | β | 0.01 | 0.00 | 0.02 | 0.16 | 0 | β | 0 | 0 | 0 | 0 |
| *Sex* (%male) | 17 | β | 0.00 | -0.01 | 0.01 | 0.53 | 5 | β | 0.01 | -0.01 | 0.02 | 0.59 |
| *Medication* (chlorpromazine equivalents) | 9 | β | 0.00 | 0.00 | 0.00 | 0.73 | 0 | β | 0 | 0 | 0 | 0 |
| *Diagnosis (%SSD)* | 15 | β | -0.01 | -0.02 | 0.00 | 0.13 | 0 | β | 0 | 0 | 0 | 0 |

*Note.* a = Number of "yes"; b = Results must be interpreted cautiously because of the low number of studies per subgroup for some analyses; β = Beta coefficient for metaregression; *g* = Hedges' g; GP = general practitioner N/R = not reported; Q = Q-statistic for between-group comparison; SSD = Schizophrenia-spectrum disorder; gender (i.e., including non-binary, other, etc. categories) was not included as a moderator as it was not assessed by any study.

<table>
<tr><th rowspan="2">Moderator</th><th colspan="6">Quality of life[b]</th><th colspan="6">Functioning[b]</th></tr>
<tr><th>Nb studies</th><th>Statistic Type</th><th>Statistic Value</th><th>Lower 95% CI</th><th>Upper 95% CI</th><th>p-value</th><th>Nb studies</th><th>Statistic Type</th><th>Statistic Value</th><th>Lower 95% CI</th><th>Upper 95% CI</th><th>p-value</th></tr>
<tr><td>Study characteristics</td><td></td><td></td><td></td><td></td><td></td><td></td><td></td><td></td><td></td><td></td><td></td><td></td></tr>
<tr><td>Publication year</td><td>7</td><td>β</td><td>-0.08</td><td>-0.19</td><td>0.03</td><td>0.15</td><td>13</td><td>β</td><td>-0.07</td><td>-0.18</td><td>0.03</td><td>0.17</td></tr>
<tr><td>MMAT assessment[a]</td><td></td><td></td><td></td><td></td><td></td><td></td><td></td><td></td><td></td><td></td><td></td><td></td></tr>
<tr><td>1 (lowest quality)</td><td>0</td><td>g</td><td>0.00</td><td>0.00</td><td>0.00</td><td>0.00</td><td>1</td><td>g</td><td>-0.14</td><td>-0.72</td><td>0.43</td><td>0.62</td></tr>
<tr><td>2</td><td>1</td><td>g</td><td>0.24</td><td>-0.08</td><td>0.56</td><td>0.14</td><td>4</td><td>g</td><td>0.49</td><td>0.02</td><td>0.97</td><td>0.04</td></tr>
<tr><td>3</td><td>2</td><td>g</td><td>-0.05</td><td>-0.42</td><td>0.33</td><td>0.80</td><td>3</td><td>g</td><td>0.02</td><td>-0.26</td><td>0.29</td><td>0.91</td></tr>
<tr><td>4</td><td>1</td><td>g</td><td>0.28</td><td>-0.08</td><td>0.64</td><td>0.13</td><td>2</td><td>g</td><td>0.80</td><td>-1.10</td><td>2.71</td><td>0.41</td></tr>
<tr><td>5 (highest quality)</td><td>3</td><td>g</td><td>0.39</td><td>-0.51</td><td>1.29</td><td>0.40</td><td>3</td><td>g</td><td>0.74</td><td>0.33</td><td>1.15</td><td>0.00</td></tr>
<tr><td>Subgroup differences</td><td></td><td>$Q_3$</td><td>2.06</td><td></td><td></td><td>0.56</td><td></td><td>$Q_4$</td><td>11.43</td><td></td><td></td><td>0.02</td></tr>
<tr><td>Type of analysis</td><td></td><td></td><td></td><td></td><td></td><td></td><td></td><td></td><td></td><td></td><td></td><td></td></tr>
<tr><td>N/R</td><td>0</td><td>g</td><td>0.00</td><td>0.00</td><td>0.00</td><td>0.00</td><td>4</td><td>g</td><td>0.30</td><td>-0.32</td><td>0.92</td><td>0.34</td></tr>
<tr><td>Intention-to-treat</td><td>2</td><td>g</td><td>0.20</td><td>-0.08</td><td>0.48</td><td>0.16</td><td>3</td><td>g</td><td>0.51</td><td>0.26</td><td>0.76</td><td>0.00</td></tr>
<tr><td>Per-protocol</td><td>5</td><td>g</td><td>0.23</td><td>-0.20</td><td>0.66</td><td>0.29</td><td>6</td><td>g</td><td>0.41</td><td>-0.08</td><td>0.90</td><td>0.10</td></tr>
<tr><td>Subgroup differences</td><td></td><td>$Q_1$</td><td>0.02</td><td></td><td></td><td>0.90</td><td></td><td>$Q_2$</td><td>0.43</td><td></td><td></td><td>0.81</td></tr>
<tr><td>Type of control condition</td><td></td><td></td><td></td><td></td><td></td><td></td><td></td><td></td><td></td><td></td><td></td><td></td></tr>
<tr><td>Active</td><td>2</td><td>g</td><td>0.20</td><td>-0.11</td><td>0.52</td><td>0.20</td><td>4</td><td>g</td><td>-0.01</td><td>-0.29</td><td>0.27</td><td>0.96</td></tr>
<tr><td>None</td><td>2</td><td>g</td><td>0.14</td><td>-0.12</td><td>0.41</td><td>0.29</td><td>3</td><td>g</td><td>0.54</td><td>0.04</td><td>1.05</td><td>0.04</td></tr>
<tr><td>Other</td><td>0</td><td>g</td><td>0.00</td><td>0.00</td><td>0.00</td><td>0.00</td><td>1</td><td>g</td><td>1.34</td><td>0.78</td><td>1.90</td><td>0.00</td></tr>
<tr><td>Passive</td><td>3</td><td>g</td><td>0.39</td><td>-0.51</td><td>1.29</td><td>0.40</td><td>5</td><td>g</td><td>0.45</td><td>-0.02</td><td>0.92</td><td>0.06</td></tr>
<tr><td>Subgroup differences</td><td></td><td>$Q_2$</td><td>0.30</td><td></td><td></td><td>0.86</td><td></td><td>$Q_3$</td><td>19.13</td><td></td><td></td><td>0.00</td></tr>
<tr><td>Type of design</td><td></td><td></td><td></td><td></td><td></td><td></td><td></td><td></td><td></td><td></td><td></td><td></td></tr>
<tr><td>Non-randomized</td><td>3</td><td>g</td><td>0.42</td><td>-0.19</td><td>1.02</td><td>0.17</td><td>4</td><td>g</td><td>0.72</td><td>0.20</td><td>1.23</td><td>0.00</td></tr>
<tr><td>Randomized controlled</td><td>4</td><td>g</td><td>0.13</td><td>-0.13</td><td>0.39</td><td>0.32</td><td>9</td><td>g</td><td>0.26</td><td>-0.05</td><td>0.57</td><td>0.00</td></tr>
<tr><td>Subgroup differences</td><td></td><td>$Q_1$</td><td>0.72</td><td></td><td></td><td>0.40</td><td></td><td>$Q_1$</td><td>2.18</td><td></td><td></td><td>0.14</td></tr>
<tr><td>Treatment characteristics</td><td></td><td></td><td></td><td></td><td></td><td></td><td></td><td></td><td></td><td></td><td></td><td></td></tr>
<tr><td>Nb MCT sessions</td><td></td><td></td><td></td><td></td><td></td><td></td><td></td><td></td><td></td><td></td><td></td><td></td></tr>
<tr><td>Less than 8</td><td>1</td><td>g</td><td>1.37</td><td>0.56</td><td>2.17</td><td>0.00</td><td>0</td><td>g</td><td>0.00</td><td>0.00</td><td>0.00</td><td>0.00</td></tr>
<tr><td>One cycle (8)</td><td>3</td><td>g</td><td>0.15</td><td>-0.14</td><td>0.45</td><td>0.32</td><td>3</td><td>g</td><td>0.23</td><td>-0.30</td><td>0.76</td><td>0.39</td></tr>
<tr><td>Two cycles (16)</td><td>0</td><td>g</td><td>0.00</td><td>0.00</td><td>0.00</td><td>0.00</td><td>4</td><td>g</td><td>0.66</td><td>0.03</td><td>1.28</td><td>0.04</td></tr>
<tr><td>More than 16</td><td>0</td><td>g</td><td>0.00</td><td>0.00</td><td>0.00</td><td>0.00</td><td>2</td><td>g</td><td>0.64</td><td>-0.82</td><td>2.09</td><td>0.39</td></tr>
<tr><td>Other</td><td>3</td><td>g</td><td>0.13</td><td>-0.11</td><td>0.37</td><td>0.28</td><td>4</td><td>g</td><td>0.23</td><td>-0.04</td><td>0.51</td><td>0.10</td></tr>
<tr><td>Subgroup differences</td><td></td><td>$Q_2$</td><td>8.51</td><td></td><td></td><td>0.01</td><td></td><td>$Q_4$</td><td>1.74</td><td></td><td></td><td>0.63</td></tr>
<tr><td>Delivery format</td><td></td><td></td><td></td><td></td><td></td><td></td><td></td><td></td><td></td><td></td><td></td><td></td></tr>
<tr><td>N/R</td><td>0</td><td>g</td><td>0.00</td><td>0.00</td><td>0.00</td><td>0.00</td><td>1</td><td>g</td><td>1.34</td><td>0.78</td><td>1.90</td><td>0.00</td></tr>
<tr><td>Both</td><td>0</td><td>g</td><td>0.00</td><td>0.00</td><td>0.00</td><td>0.00</td><td>0</td><td>g</td><td>0.00</td><td>0.00</td><td>0.00</td><td>0.00</td></tr>
<tr><td>Individual</td><td>6</td><td>g</td><td>0.14</td><td>-0.05</td><td>0.32</td><td>0.14</td><td>11</td><td>g</td><td>0.37</td><td>0.09</td><td>0.66</td><td>0.01</td></tr>
<tr><td>Group</td><td>1</td><td>g</td><td>1.37</td><td>0.56</td><td>2.17</td><td>0.00</td><td>1</td><td>g</td><td>-0.14</td><td>-0.72</td><td>0.43</td><td>0.62</td></tr>
</table>

| Moderator | Quality of life[b] | | | | | | Functioning[b] | | | | | |
|---|---|---|---|---|---|---|---|---|---|---|---|---|
| | Nb studies | Statistic Type | Statistic Value | Lower 95% CI | Upper 95% CI | *p*-value | Nb studies | Statistic Type | Statistic Value | Lower 95% CI | Upper 95% CI | *p*-value |
| Subgroup differences | | $Q_1$ | 8.51 | | | 0.00 | | $Q_2$ | 14.19 | | | 0.00 |
| *Facilitators credentials* | | | | | | | | | | | | |
| N/R | 1 | *g* | 1.37 | 0.56 | 2.17 | 0.00 | 2 | *g* | 0.64 | -0.82 | 2.09 | 0.39 |
| GP | 1 | *g* | 0.28 | -0.08 | 0.64 | 0.13 | 0 | *g* | 0.00 | 0.00 | 0.00 | 0.00 |
| Graduate student | 0 | *g* | 0.00 | 0.00 | 0.00 | 0.00 | 0 | *g* | 0.00 | 0.00 | 0.00 | 0.00 |
| Nurse | 1 | *g* | 0.24 | -0.08 | 0.56 | 0.14 | 2 | *g* | 0.57 | 0.28 | 0.85 | 0.00 |
| Occupational therapist | 1 | *g* | 0.06 | -0.51 | 0.64 | 0.83 | 2 | *g* | 0.19 | -0.11 | 0.49 | 0.21 |
| Psychiatrist | 0 | *g* | 0.00 | 0.00 | 0.00 | 0.00 | 2 | *g* | 0.88 | -0.80 | 2.56 | 0.30 |
| Psychologist | 3 | *g* | -0.08 | -0.42 | 0.26 | 0.65 | 5 | *g* | 0.20 | -0.33 | 0.73 | 0.47 |
| Subgroup differences | | $Q_4$ | 11.09 | | | 0.03 | | $Q_5$ | 4.08 | | | 0.40 |
| *Training of facilitators* | | | | | | | | | | | | |
| No or N/R | 1 | *g* | 1.37 | 0.56 | 2.17 | 0.00 | 2 | *g* | 0.72 | -0.45 | 1.90 | 0.23 |
| Yes | 6 | *g* | 0.14 | -0.05 | 0.32 | 0.14 | 11 | *g* | 0.35 | 0.05 | 0.65 | 0.02 |
| Subgroup differences | | $Q_1$ | 8.51 | | | 0.00 | | $Q_1$ | 0.36 | | | 0.55 |
| *Adherence to MCT manual* | | | | | | | | | | | | |
| No or N/R | 3 | *g* | 0.48 | 0.01 | 0.95 | 0.04 | 4 | *g* | 0.83 | 0.14 | 1.53 | 0.02 |
| Yes | 4 | *g* | -0.04 | -0.34 | 0.25 | 0.78 | 9 | *g* | 0.26 | -0.05 | 0.57 | 0.10 |
| Subgroup differences | | $Q_1$ | 3.45 | | | 0.06 | | $Q_1$ | 2.15 | | | 0.14 |
| **Participant characteristics** | | | | | | | | | | | | |
| *Age* (years) | 7 | β | 0.00 | -0.04 | 0.04 | 0.92 | 12 | β | 0.01 | -0.02 | 0.04 | 0.45 |
| *Duration of illness* (years) | 6 | β | 0.01 | -0.05 | 0.07 | 0.67 | 9 | β | 0.01 | -0.02 | 0.04 | 0.49 |
| *Sex* (%male) | 7 | β | 0.00 | -0.01 | 0.02 | 0.75 | 13 | β | 0.02 | -0.01 | 0.04 | 0.14 |
| *Medication* (chlorpromazine equivalents) | 4 | β | 0.00 | 0.00 | 0.00 | 0.67 | 7 | β | 0.00 | 0.00 | 0.00 | 0.50 |
| *Diagnosis (%SSD)* | 7 | β | 0.01 | -0.02 | 0.05 | 0.43 | 12 | β | -0.02 | -0.05 | 0.01 | 0.25 |

*Note.* a = Number of "yes"; b = Results must be interpreted cautiously because of the low number of studies per subgroup for some analyses; β = Beta coefficient for metaregression; *g* = Hedges' g; GP = general practitioner N/R = not reported; Q = Q-statistic for between-group comparison; SSD = Schizophrenia-spectrum disorders; gender (i.e., including non-binary, other, etc. categories) was not included as a moderator as it was not assessed by any study.

**eFigure 2. Scatterplot of publication year significantly moderating effect sizes for hallucinations in pre-post comparison.**

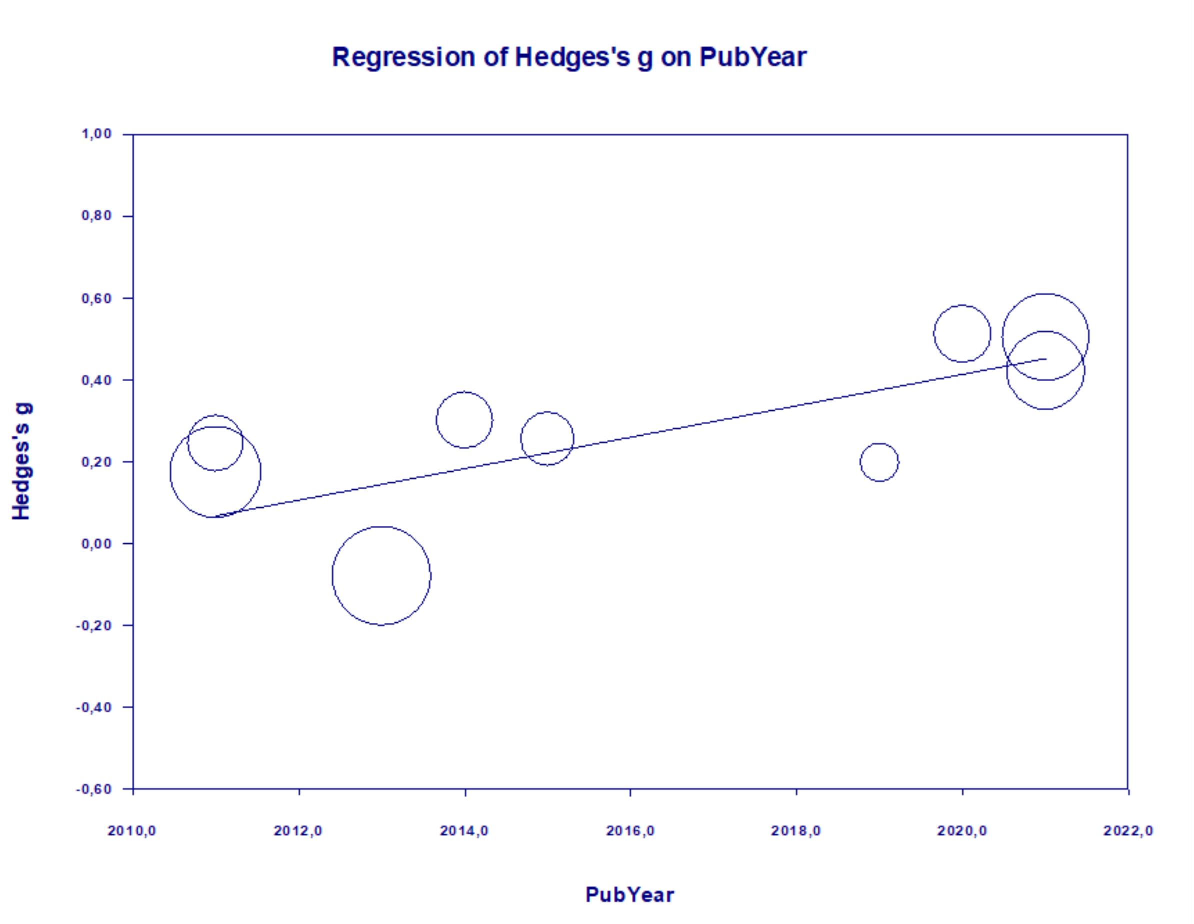

## eTable 11. Heterogeneity assessment by outcome and timepoints comparison.

| Comparison | Outcome | Nb studies | Nb participants | Q-value | *df* | *p*-value | $I^2$ |
|---|---|---|---|---|---|---|---|
| Pre-Post | Proximal | 38 | 1717 | 101.03 | 37 | 0.00 | 63.38 |
| Pre-Post | Positive sx | 36 | 1648 | 144.37 | 35 | 0.00 | 75.76 |
| Pre-Post | Delusions | 23 | 1156 | 121.13 | 22 | 0.00 | 81.84 |
| Pre-Post | Hallucinations | 9 | 518 | 7.84 | 8 | 0.45 | 0.00 |
| Pre-Post | Cognitive bias | 19 | 931 | 23.57 | 18 | 0.17 | 23.63 |
| Pre-Post | Distal | 26 | 1180 | 42.83 | 25 | 0.01 | 41.63 |
| Pre-Post | Negative sx | 17 | 765 | 22.77 | 16 | 0.12 | 29.72 |
| Pre-Post | Self-esteem | 5 | 325 | 1.86 | 4 | 0.76 | 0.00 |
| Pre-Post | Quality of life | 7 | 278 | 11.25 | 6 | 0.08 | 46.65 |
| Pre-Post | Functioning | 13 | 522 | 52.45 | 12 | 0.00 | 77.12 |
| Post-FUP<1y | Proximal | 14 | 832 | 10.03 | 13 | 0.69 | 0.00 |
| Post-FUP<1y | Positive sx | 14 | 832 | 11.01 | 13 | 0.61 | 0.00 |
| Post-FUP<1y | Delusions | 10 | 654 | 13.39 | 9 | 0.15 | 32.78 |
| Post-FUP<1y | Hallucinations | 2 | 202 | 2.42 | 1 | 0.12 | 58.60 |
| Post-FUP<1y | Cognitive bias | 10 | 658 | 14.59 | 9 | 0.10 | 38.33 |
| Post-FUP<1y | Distal | 11 | 631 | 5.89 | 10 | 0.82 | 0.00 |
| Post-FUP<1y | Negative sx | 6 | 342 | 1.83 | 5 | 0.87 | 0.00 |
| Post-FUP<1y | Self-esteem | 3 | 269 | 0.83 | 2 | 0.66 | 0.00 |
| Post-FUP<1y | Quality of life | 4 | 159 | 1.29 | 3 | 0.73 | 0.00 |
| Post-FUP<1y | Functioning | 4 | 205 | 2.51 | 3 | 0.47 | 0.00 |
| Post-FUP>1y | Proximal | 3 | 328 | 0.24 | 2 | 0.89 | 0.00 |
| Post-FUP>1y | Positive sx | 2 | 178 | 0.46 | 1 | 0.50 | 0.00 |
| Post-FUP>1y | Delusions | 1 | 76 | 0.00 | 0 | 1.00 | 0.00 |
| Post-FUP>1y | Hallucinations | 1 | 74 | 0.00 | 0 | 1.00 | 0.00 |
| Post-FUP>1y | Cognitive bias | 3 | 328 | 1.27 | 2 | 0.53 | 0.00 |
| Post-FUP>1y | Distal | 1 | 28 | 0.00 | 0 | 1.00 | 0.00 |
| Post-FUP>1y | Negative sx | 1 | 28 | 0.00 | 0 | 1.00 | 0.00 |
| Post-FUP>1y | Self-esteem | 0 | 0 | 0 | 0 | 0 | 0 |
| Post-FUP>1y | Quality of life | 1 | 28 | 0.00 | 0 | 1.00 | 0.00 |
| Post-FUP>1y | Functioning | 1 | 28 | 0.00 | 0 | 1.00 | 0.00 |
| Pre-FUP<1y | Proximal | 14 | 832 | 37.71 | 13 | 0.00 | 65.52 |
| Pre-FUP<1y | Positive sx | 14 | 832 | 57.93 | 13 | 0.00 | 77.56 |
| Pre-FUP<1y | Delusions | 10 | 654 | 80.50 | 9 | 0.00 | 88.82 |
| Pre-FUP<1y | Hallucinations | 2 | 202 | 0.02 | 1 | 0.88 | 0.00 |
| Pre-FUP<1y | Cognitive bias | 10 | 658 | 19.90 | 9 | 0.02 | 54.78 |
| Pre-FUP<1y | Distal | 11 | 631 | 11.41 | 10 | 0.33 | 12.34 |
| Pre-FUP<1y | Negative sx | 6 | 342 | 5.71 | 5 | 0.34 | 12.42 |
| Pre-FUP<1y | Self-esteem | 3 | 269 | 7.00 | 2 | 0.03 | 71.44 |
| Pre-FUP<1y | Quality of life | 4 | 159 | 6.43 | 3 | 0.09 | 53.32 |
| Pre-FUP<1y | Functioning | 4 | 205 | 3.05 | 3 | 0.38 | 1.78 |

*Note.* POST-FUP<1y = Comparison of the follow-up (less than 1 year) to the post-intervention scores; Post-FUP<1y = Comparison of the follow-up (more than 1 year) to the post-intervention scores; Pre-FUP<1y = Comparison of the follow-up (less than 1 year) to the pre-intervention scores; g = Hedges' g; df = degrees of freedom; sx = symptoms.

**eTable 12. Rosenthal's Fail-safe N and tests for asymmetry of funnel plots (Egger test) by outcome for the pre-post comparison.**

| Outcome | Fail-safe N | Intercept | SE | Lower 95% CI | Upper 95% CI | *p*-value |
|---|---|---|---|---|---|---|
| Proximal | 763 | 0.65 | 0.71 | -0.79 | 2.09 | 0.36 |
| Positive symptoms | 1173 | 1.41 | 0.91 | -0.43 | 3.25 | 0.13 |
| Delusions | 871 | 2.77 | 1.61 | -0.58 | 6.11 | 0.10 |
| Hallucinations | 20 | 1.03 | 1.25 | -1.93 | 3.98 | 0.44 |
| Cognitive bias | 14 | -0.81 | 0.75 | -2.39 | 0.78 | 0.30 |
| Distal | 275 | 0.04 | 0.72 | -1.45 | 1.52 | 0.96 |
| Negative symptoms | 59 | 1.14 | 0.69 | -0.33 | 2.61 | 0.12 |
| Self-esteem | 0 | -1.41 | 0.47 | -2.89 | 0.07 | 0.06 |
| Quality of life | 2 | 0.28 | 1.67 | -4.02 | 4.57 | 0.88 |
| Functioning | 115 | -1.00 | 1.82 | -5.00 | 3.00 | 0.59 |

**eFigure 3. Funnel plots for each outcome for the pre-post comparison.**

(a) Proximal outcomes

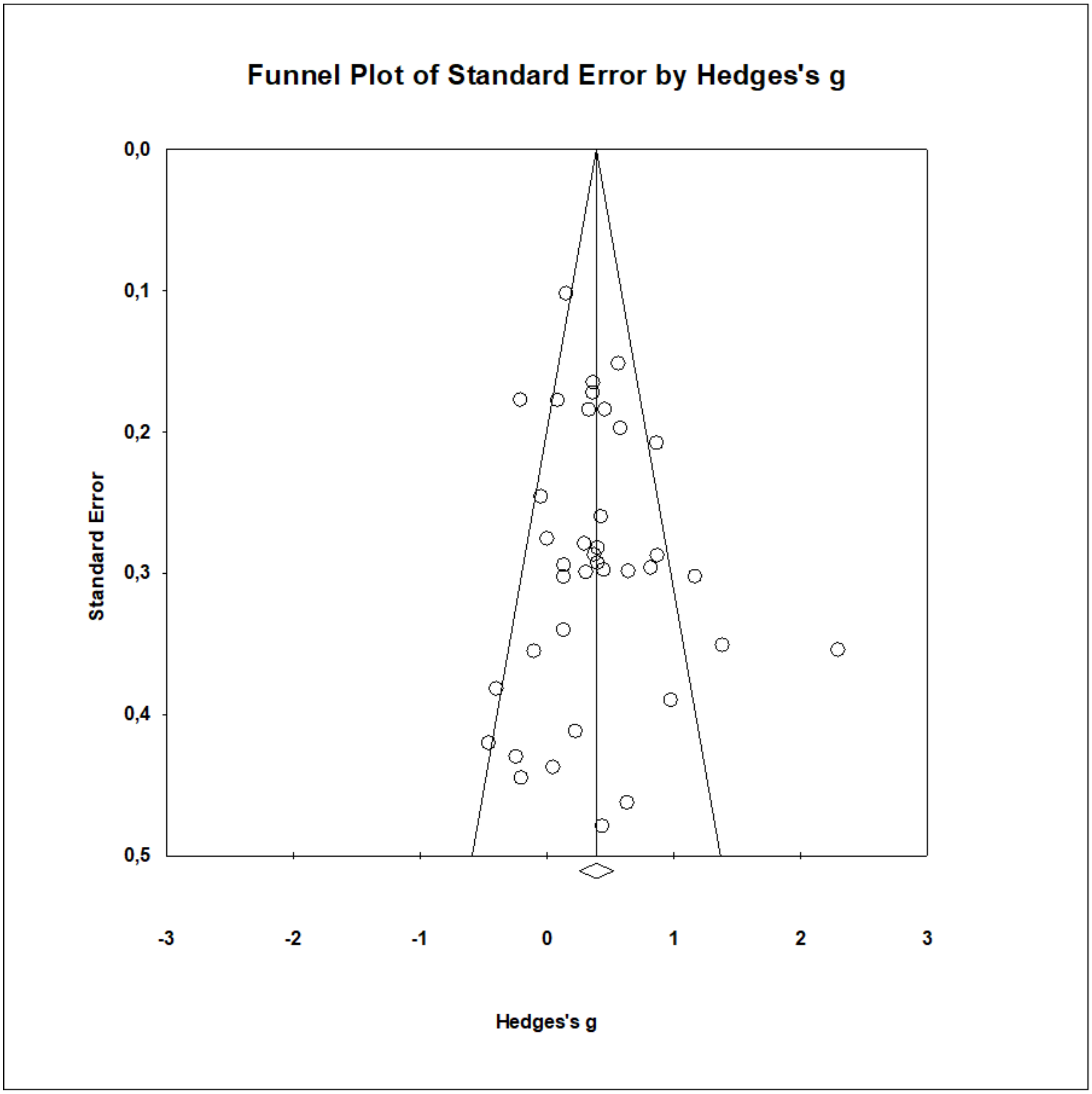

(b) Positive symptoms

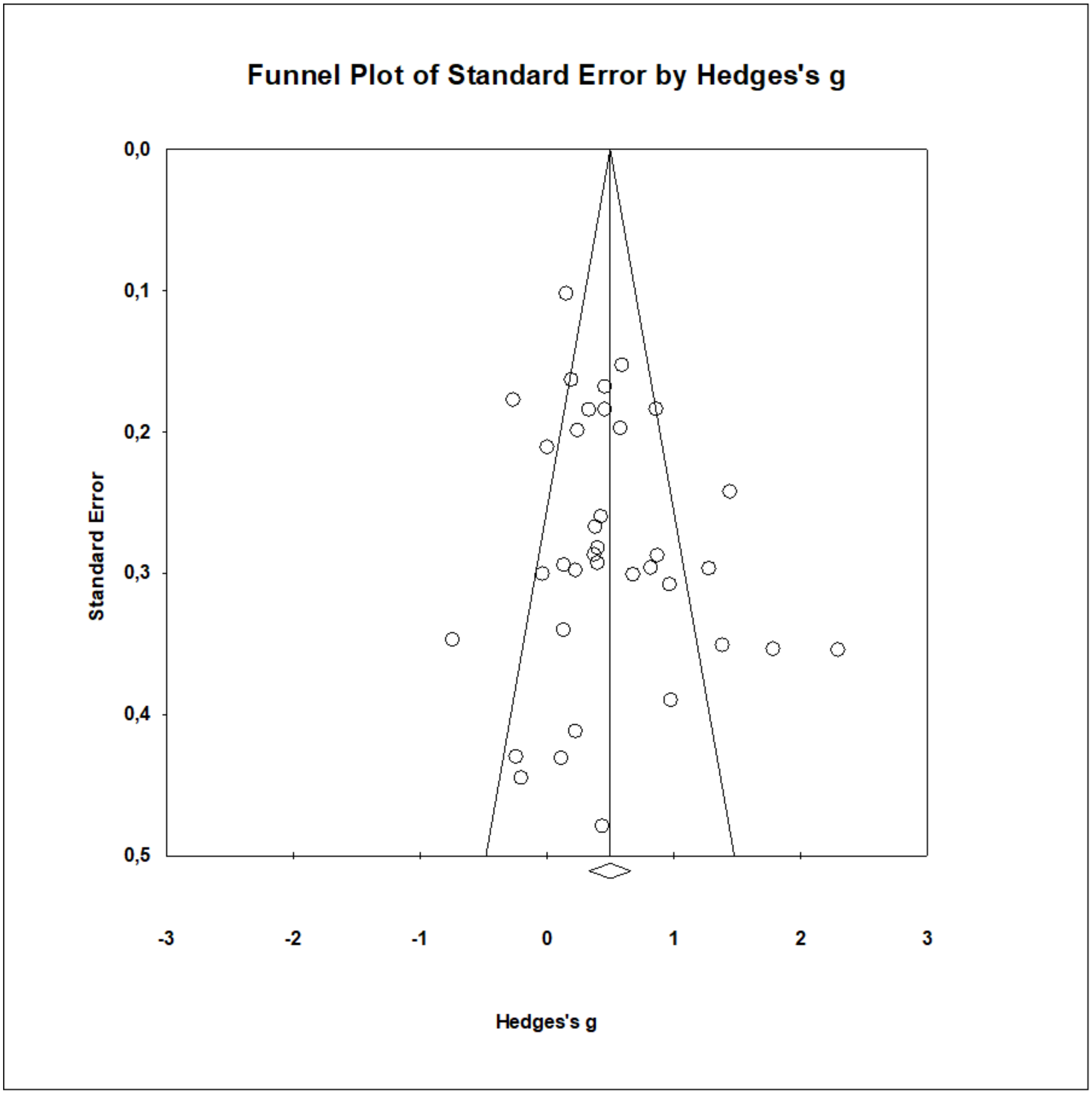

(c) Delusions

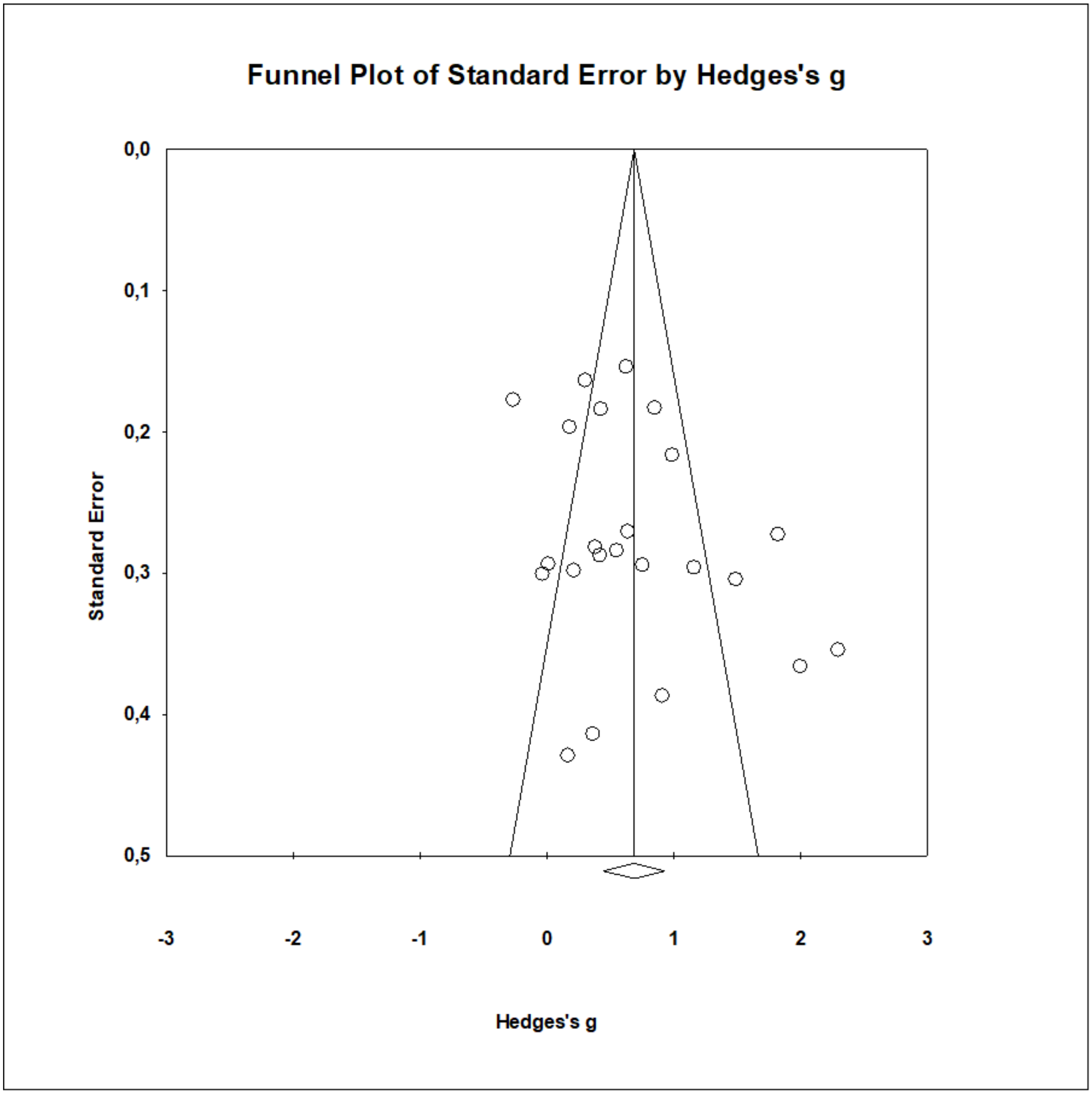

Funnel Plot of Standard Error by Hedges's g
Standard Error
0,0
0,1
0,2
0,3
0,4
0,5
-3
-2
-1
0
1
2
3
Hedges's g

(d) Hallucinations

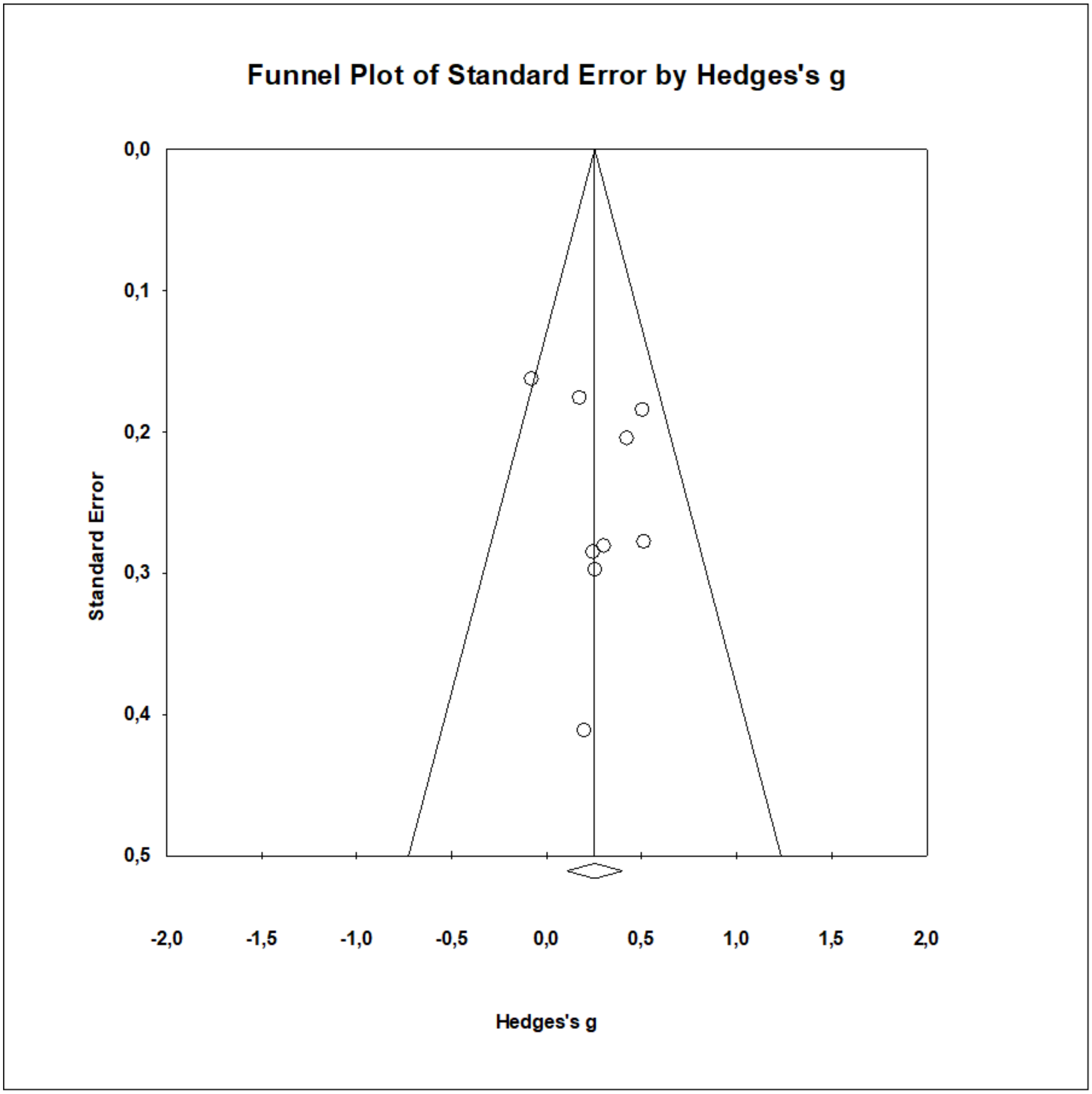

(e) Cognitive bias

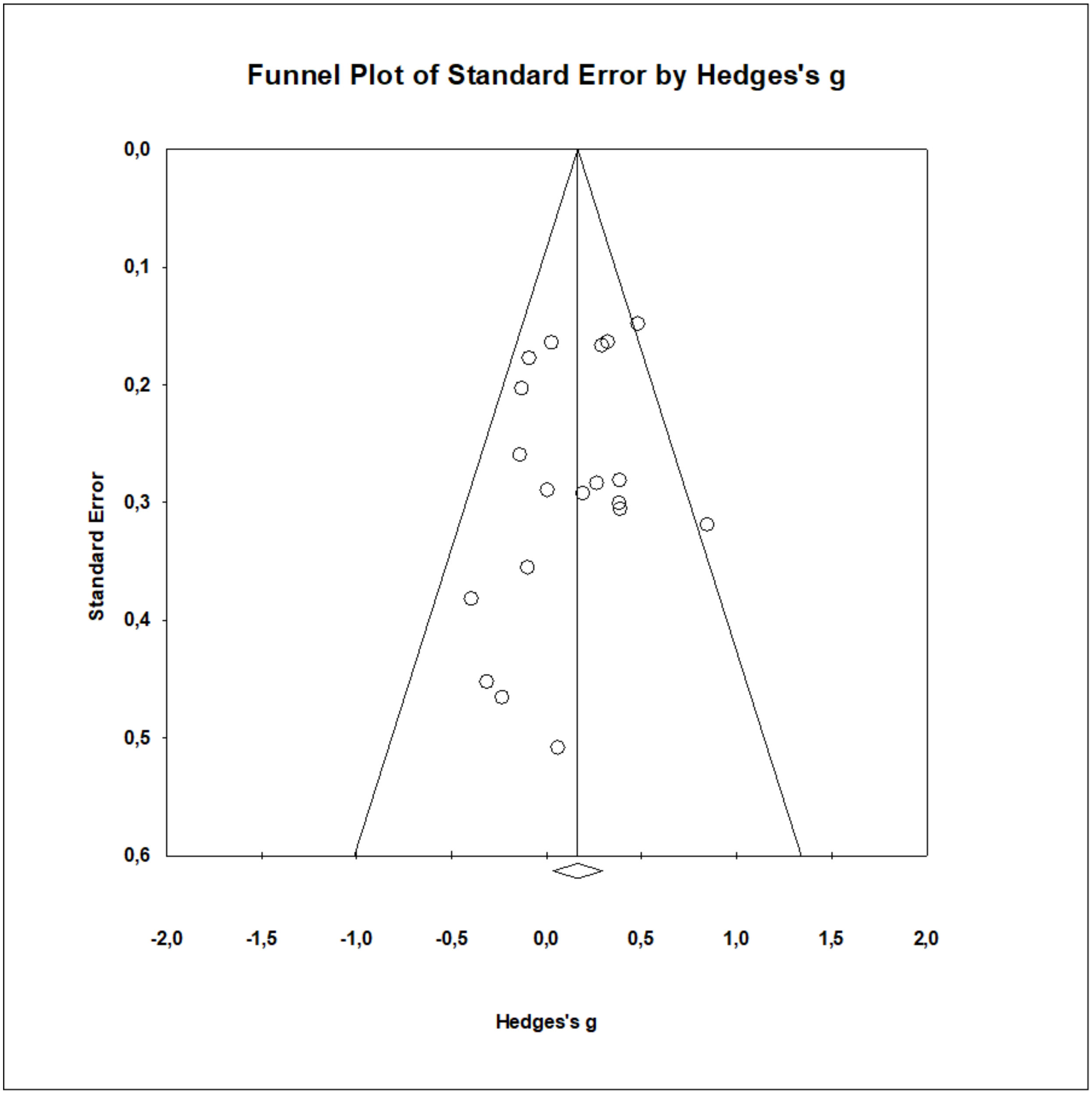

(f) Distal outcomes

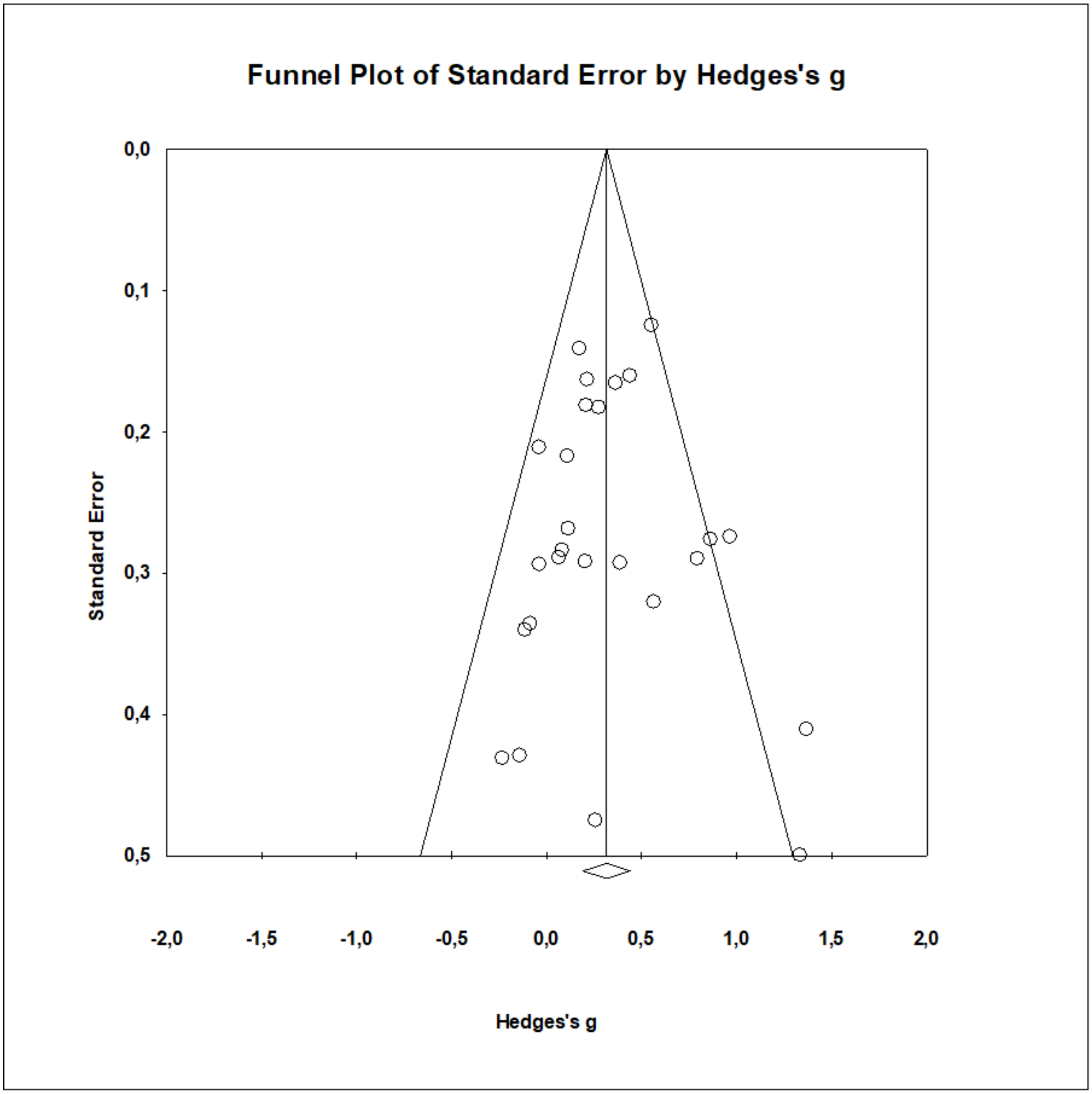

(g) Negative symptoms

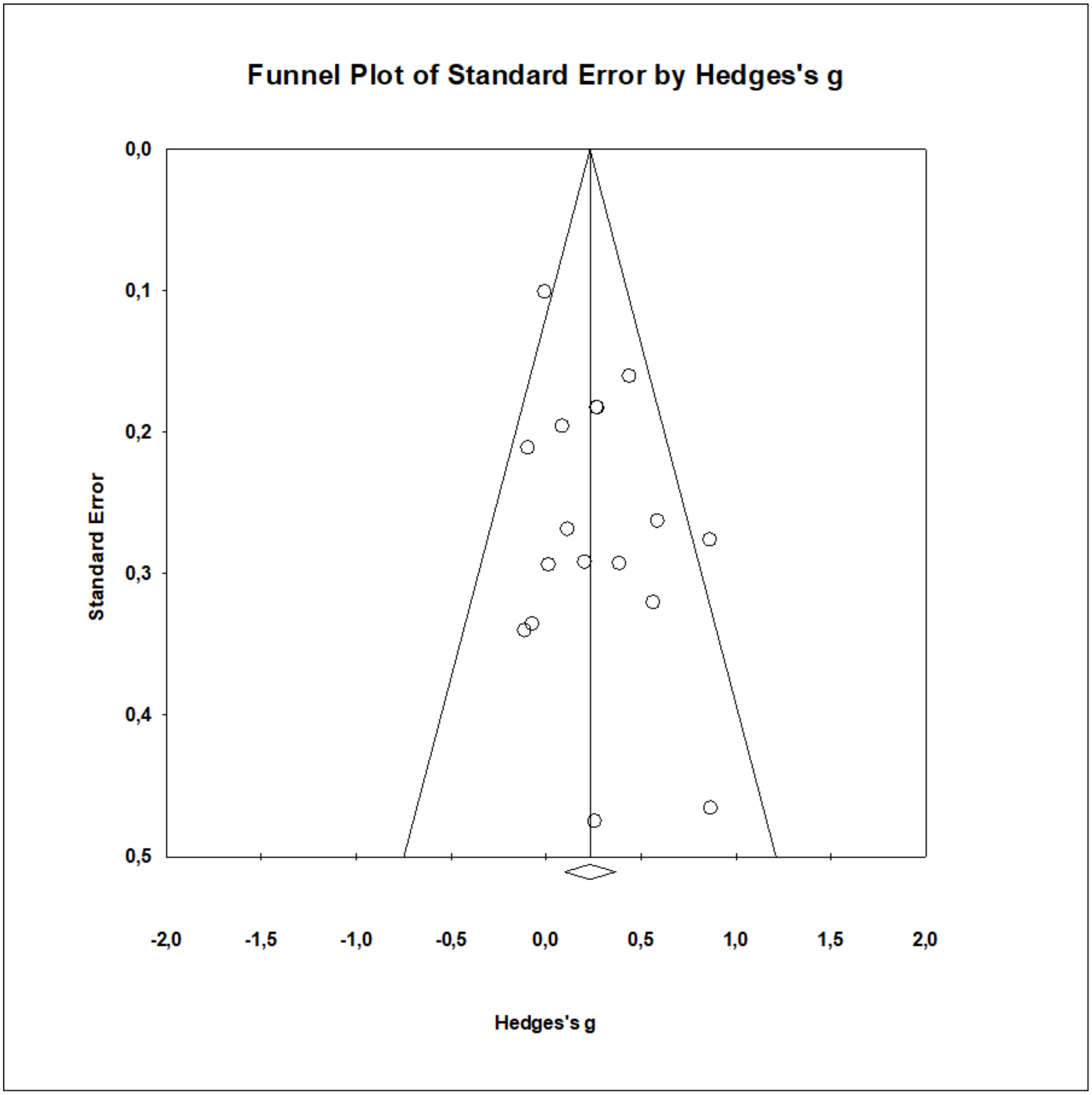

(h) Self-esteem

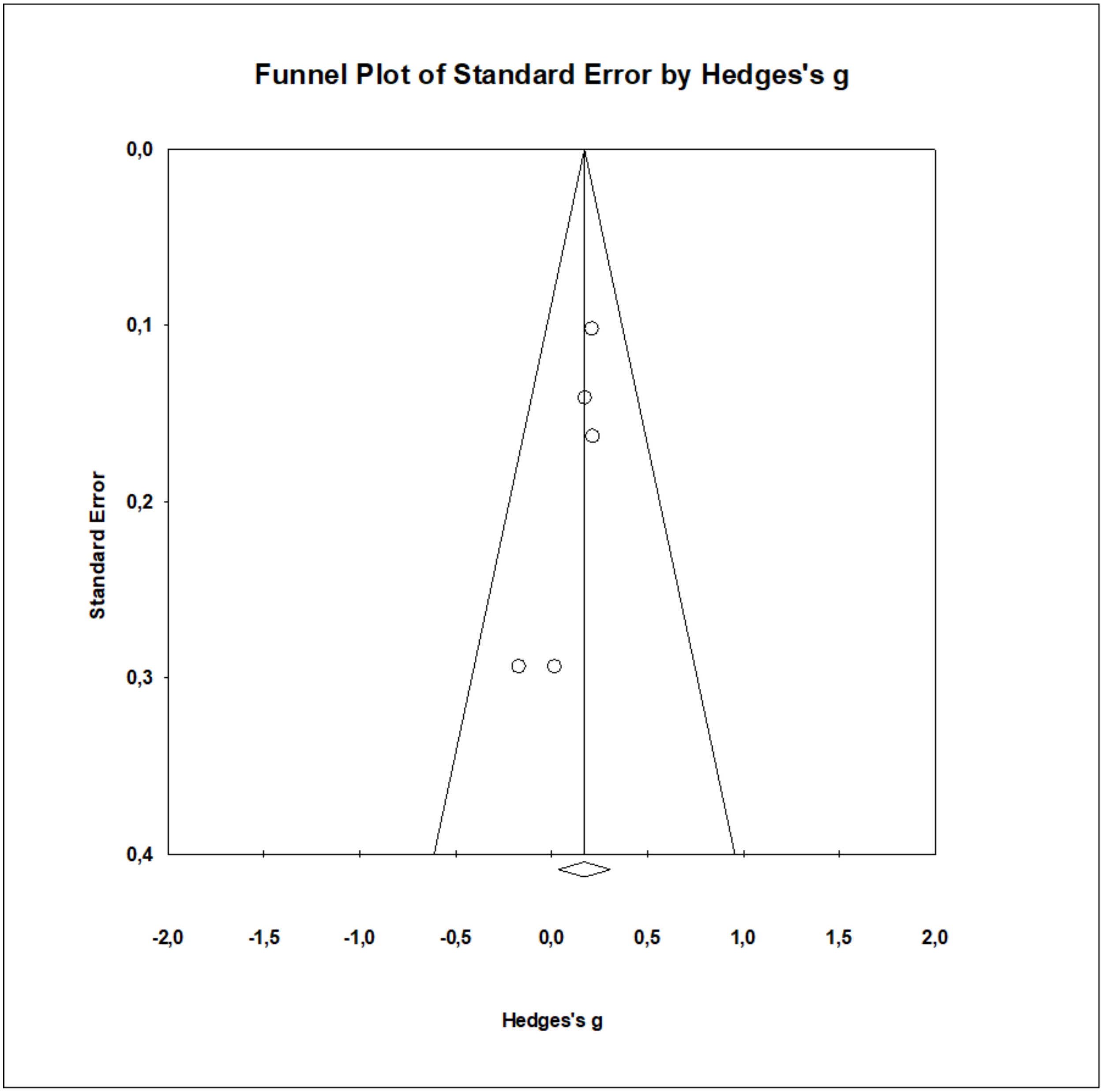

(i) Quality of life

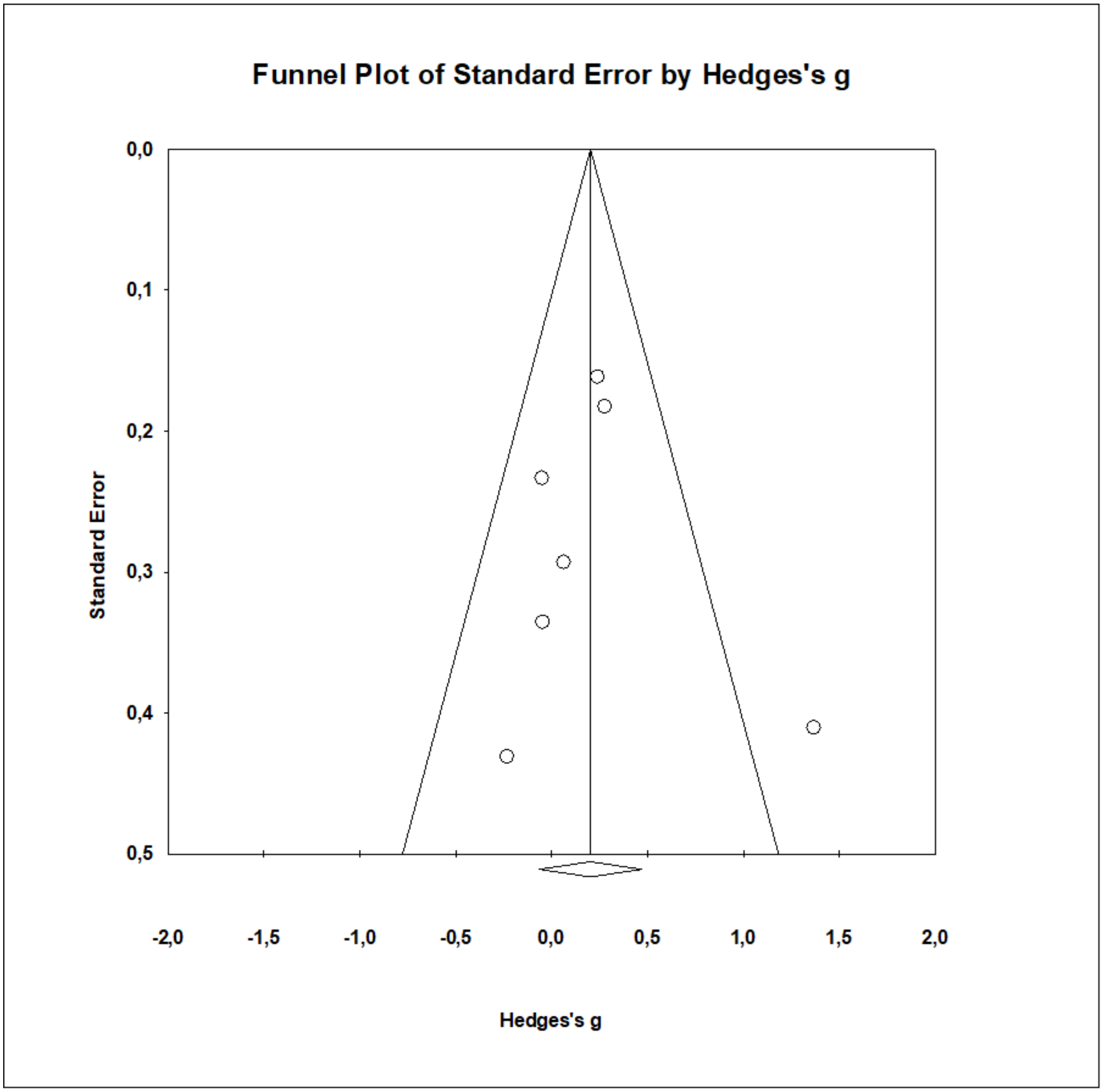

(j) Functioning

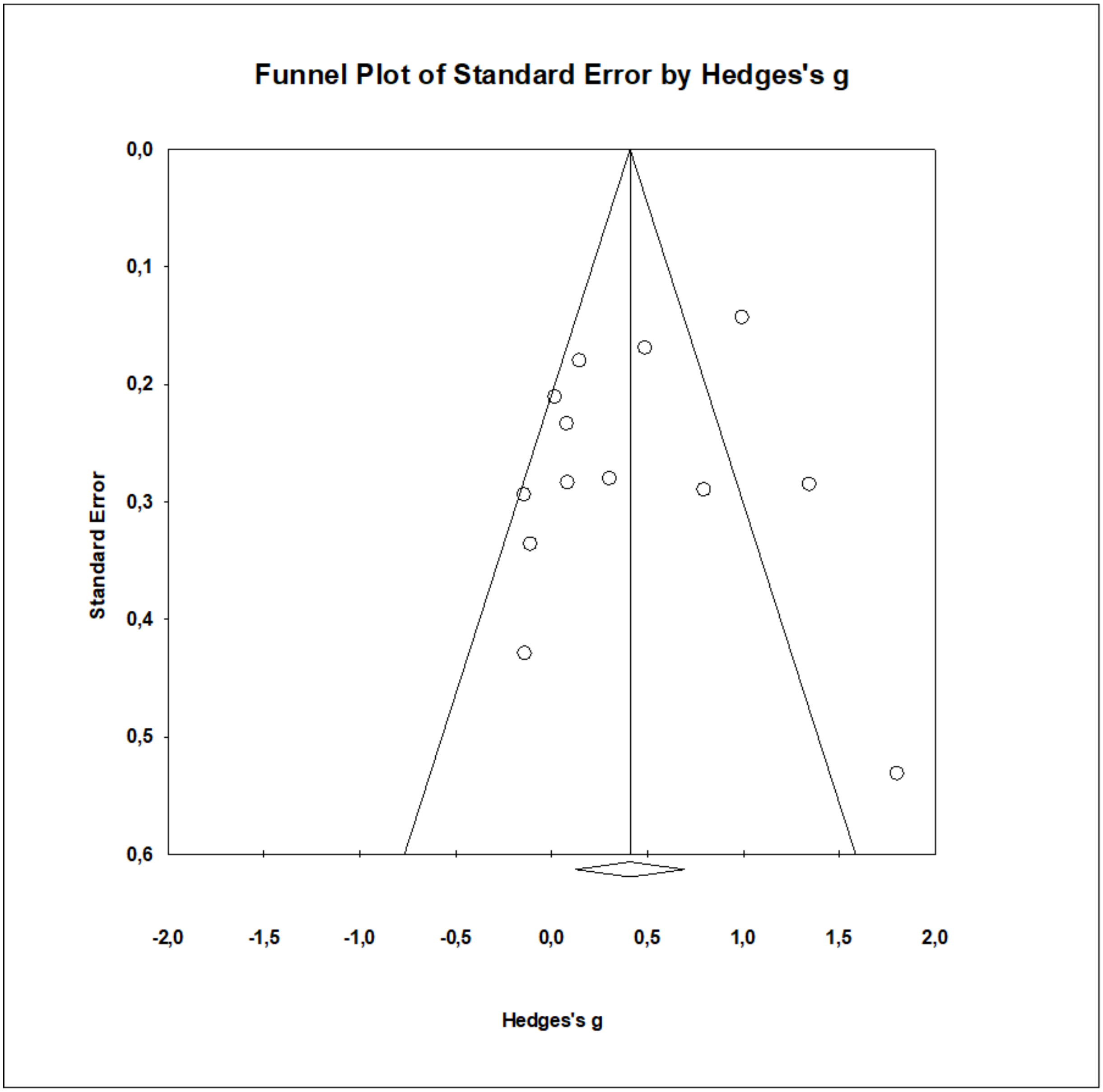

**eTable 13. Sensitivity analyses for proximal and distal outcomes measured at pre- and post-intervention.**

| Correlation between timepoints | Outcome | Nb studies | Nb participants | Estimate (*g*) | Lower 95% CI | Upper 95% CI | *p*-value |
|---|---|---|---|---|---|---|---|
| 0.5 | Proximal | 38 | 1717 | 0.34 | 0.21 | 0.46 | 0.00 |
| 0.5 | Distal | 26 | 1180 | 0.28 | 0.17 | 0.38 | 0.00 |
| 0.7 | Proximal | 38 | 1717 | 0.39 | 0.25 | 0.53 | 0.00 |
| 0.7 | Distal | 26 | 1180 | 0.32 | 0.19 | 0.44 | 0.00 |
| 0.9 | Proximal | 38 | 1717 | 0.53 | 0.38 | 0.69 | 0.00 |
| 0.9 | Distal | 26 | 1180 | 0.41 | 0.26 | 0.56 | 0.00 |

*Note.* CI = Confidence Intervals; *g* = Hedges’ g; Nb = number.

**eFigure 4. Forest plots by outcome for the pre-post comparisons including only randomized clinical trials.** **Effect sizes (Hedges' g) and their 95% confidence intervals for each study, with positive values favoring MCT and negative values favoring the control condition.**

(a) Proximal outcomes

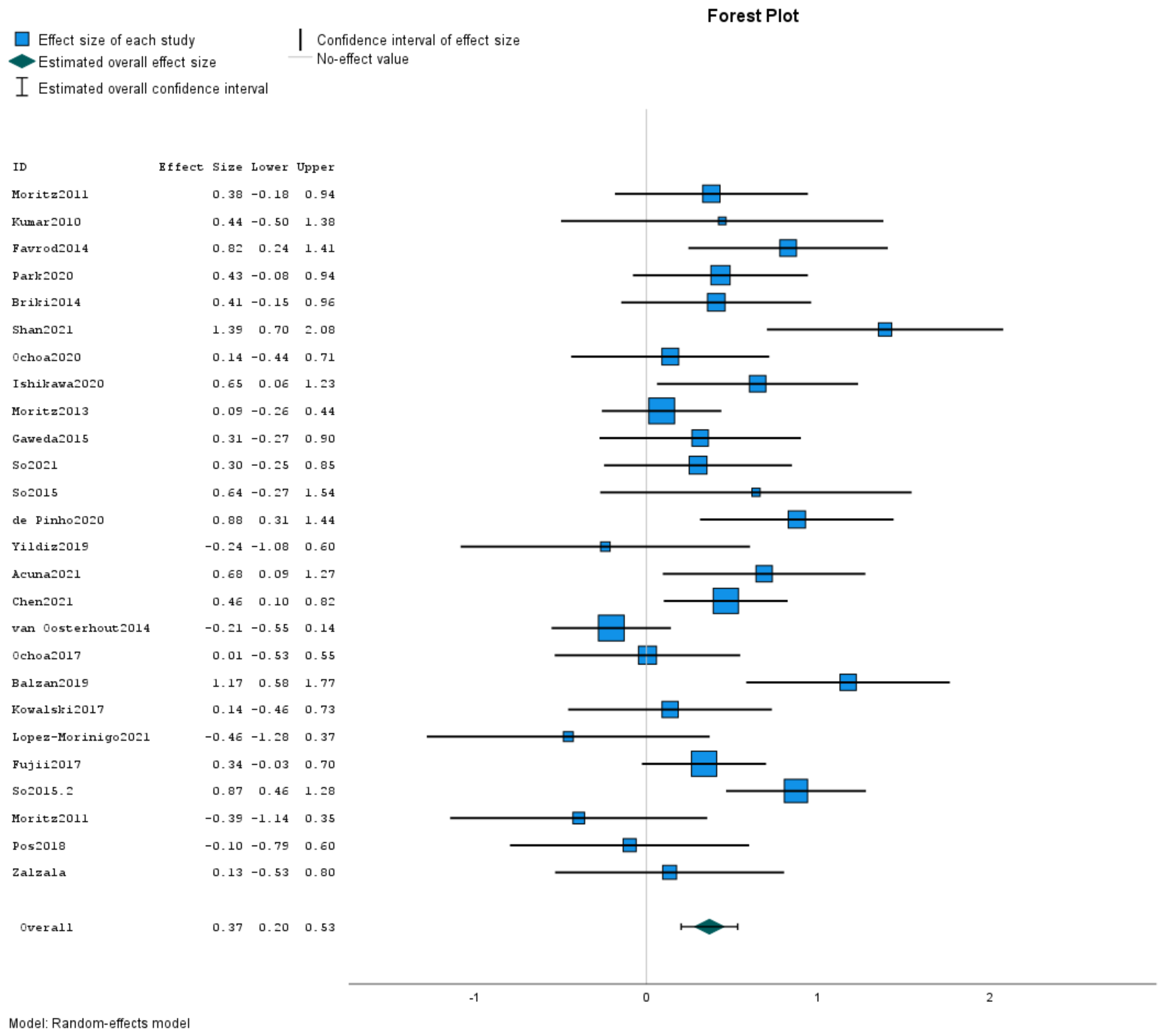

(b) Positive symptoms

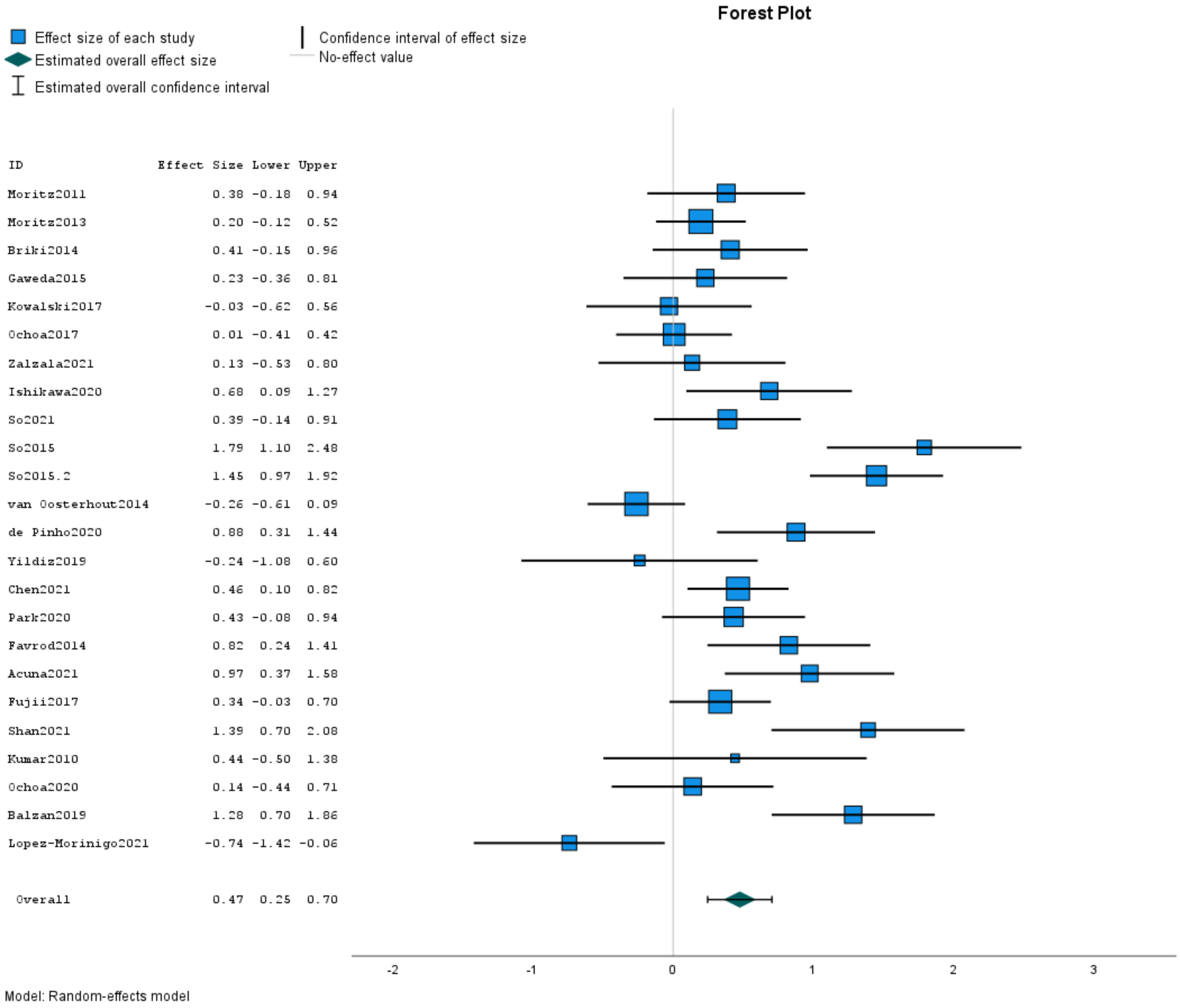

(c) Delusions

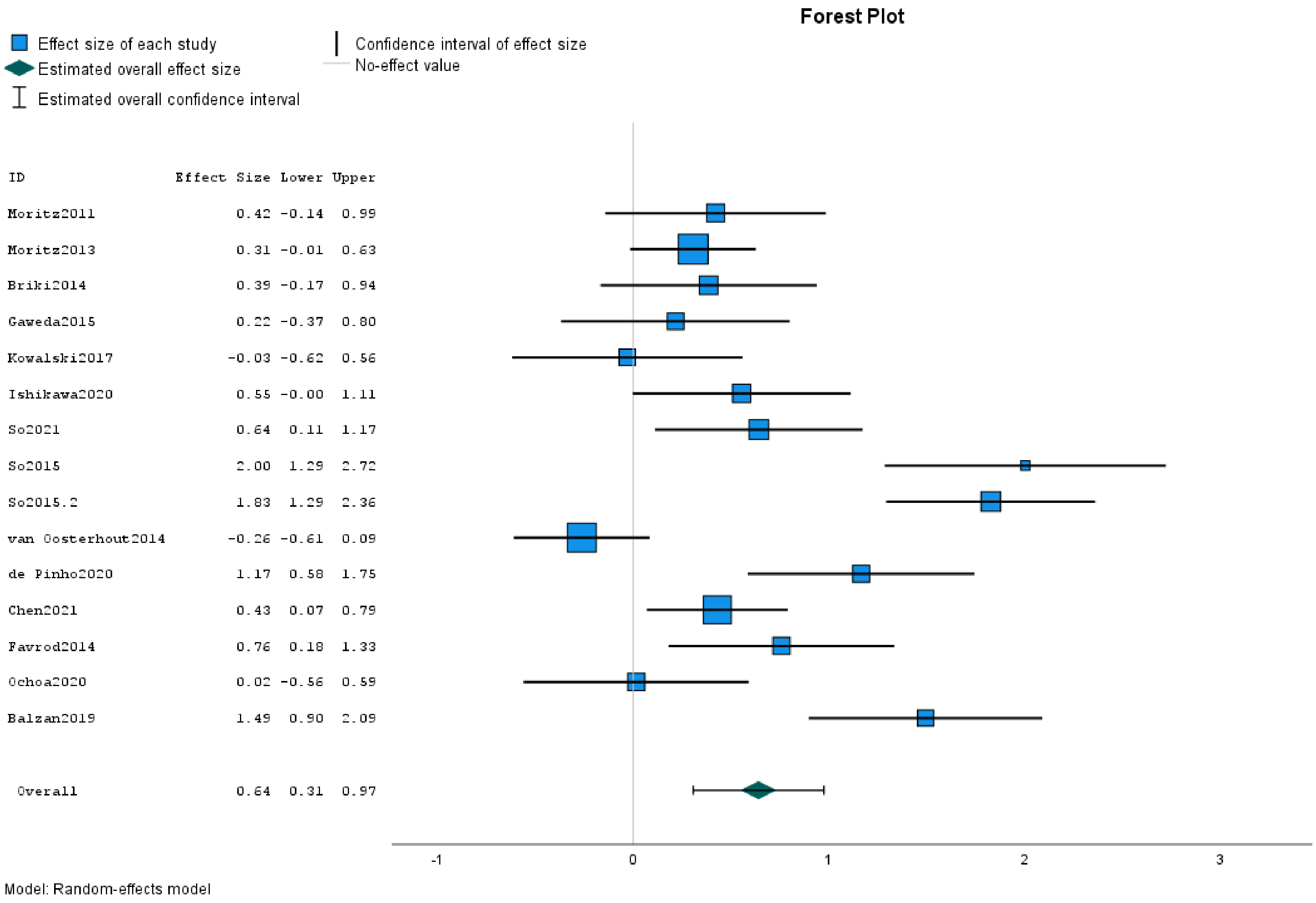


© 2022 Penney D et al. *JAMA Psychiatry*. 

(d) Hallucinations

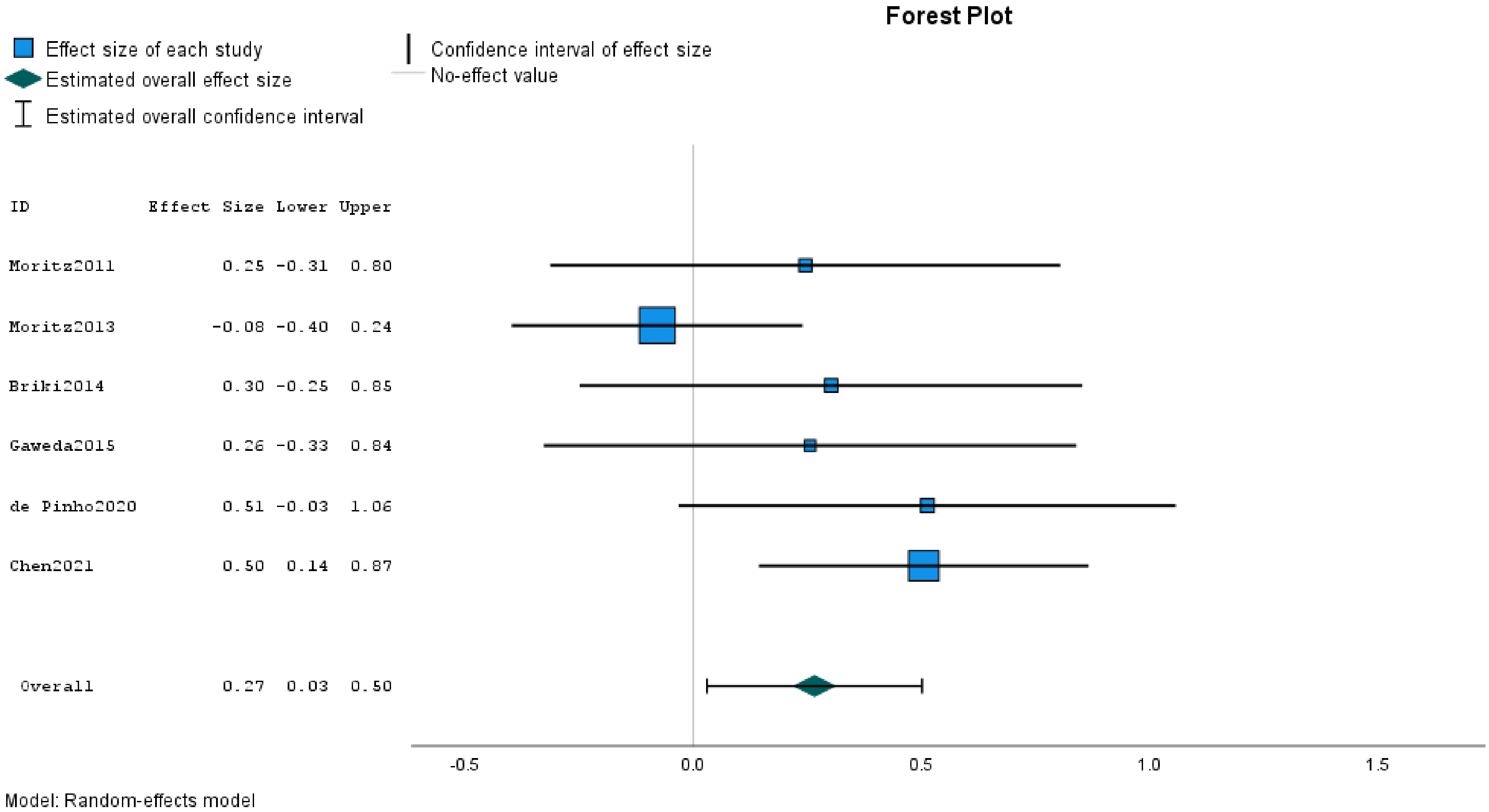

## (e) Cognitive biases

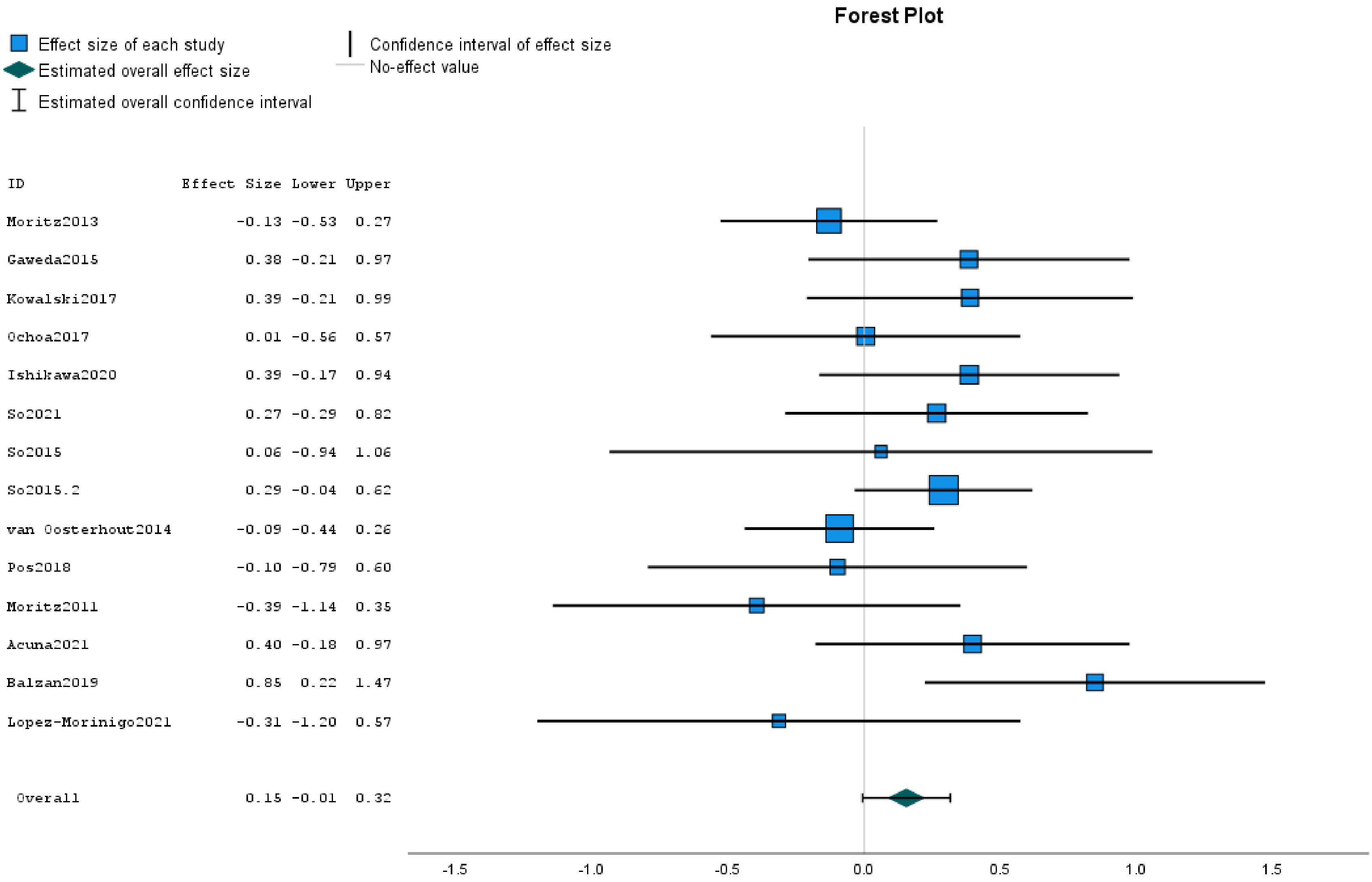


| ID | Effect Size | Lower | Upper |
|---|---|---|---|
| Moritz2013 | -0.13 | -0.53 | 0.27 |
| Gaweda2015 | 0.38 | -0.21 | 0.97 |
| Kowalski2017 | 0.39 | -0.21 | 0.99 |
| Ochoa2017 | 0.01 | -0.56 | 0.57 |
| Ishikawa2020 | 0.39 | -0.17 | 0.94 |
| So2021 | 0.27 | -0.29 | 0.82 |
| So2015 | 0.06 | -0.94 | 1.06 |
| So2015.2 | 0.29 | -0.04 | 0.62 |
| van Oosterhout2014 | -0.09 | -0.44 | 0.26 |
| Pos2018 | -0.10 | -0.79 | 0.60 |
| Moritz2011 | -0.39 | -1.14 | 0.35 |
| Acuna2021 | 0.40 | -0.18 | 0.97 |
| Balzan2019 | 0.85 | 0.22 | 1.47 |
| Lopez-Morinigo2021 | -0.31 | -1.20 | 0.57 |
| Overall | 0.15 | -0.01 | 0.32 |

Model: Random-effects model

(f) Distal outcomes

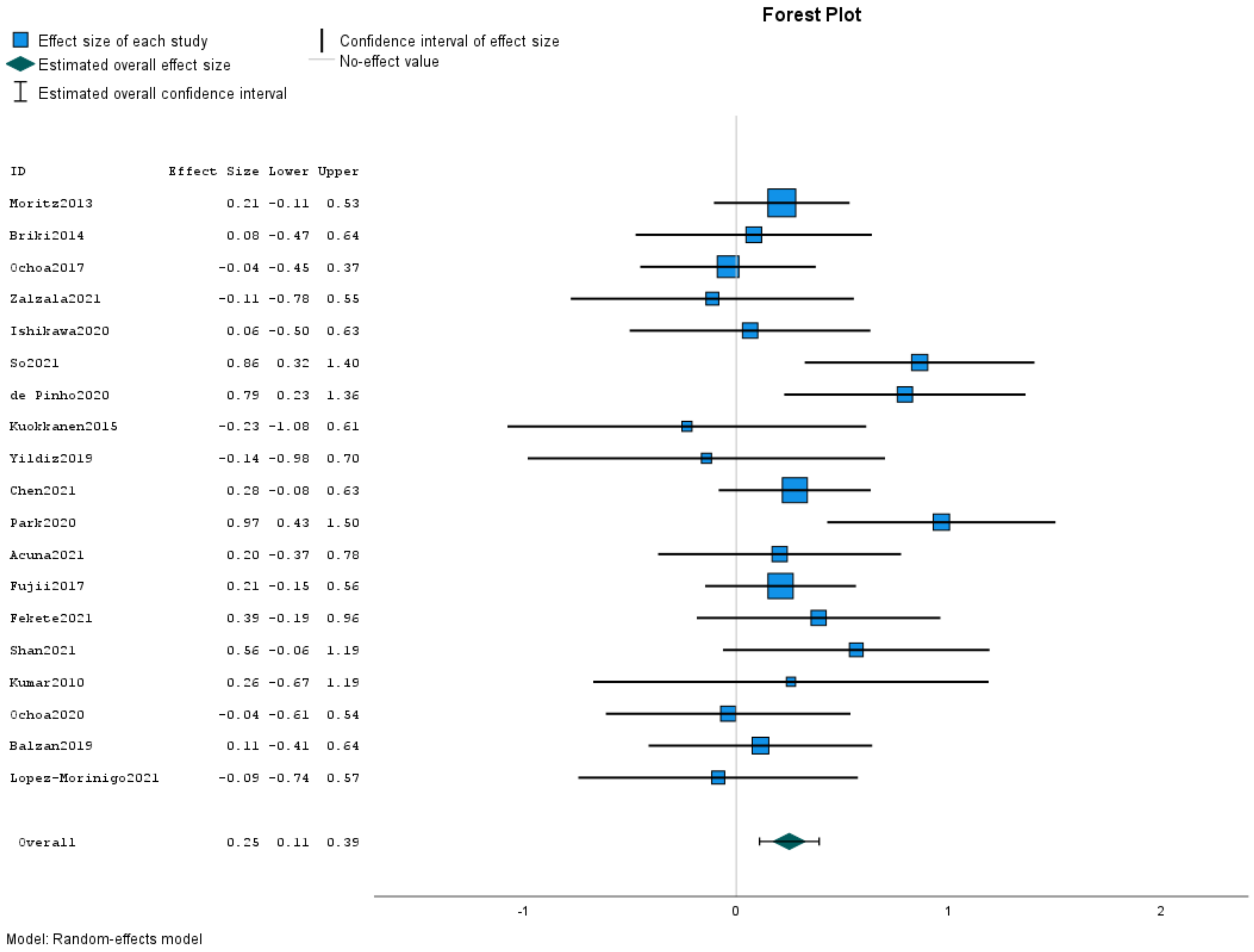

(g) Negative symptoms

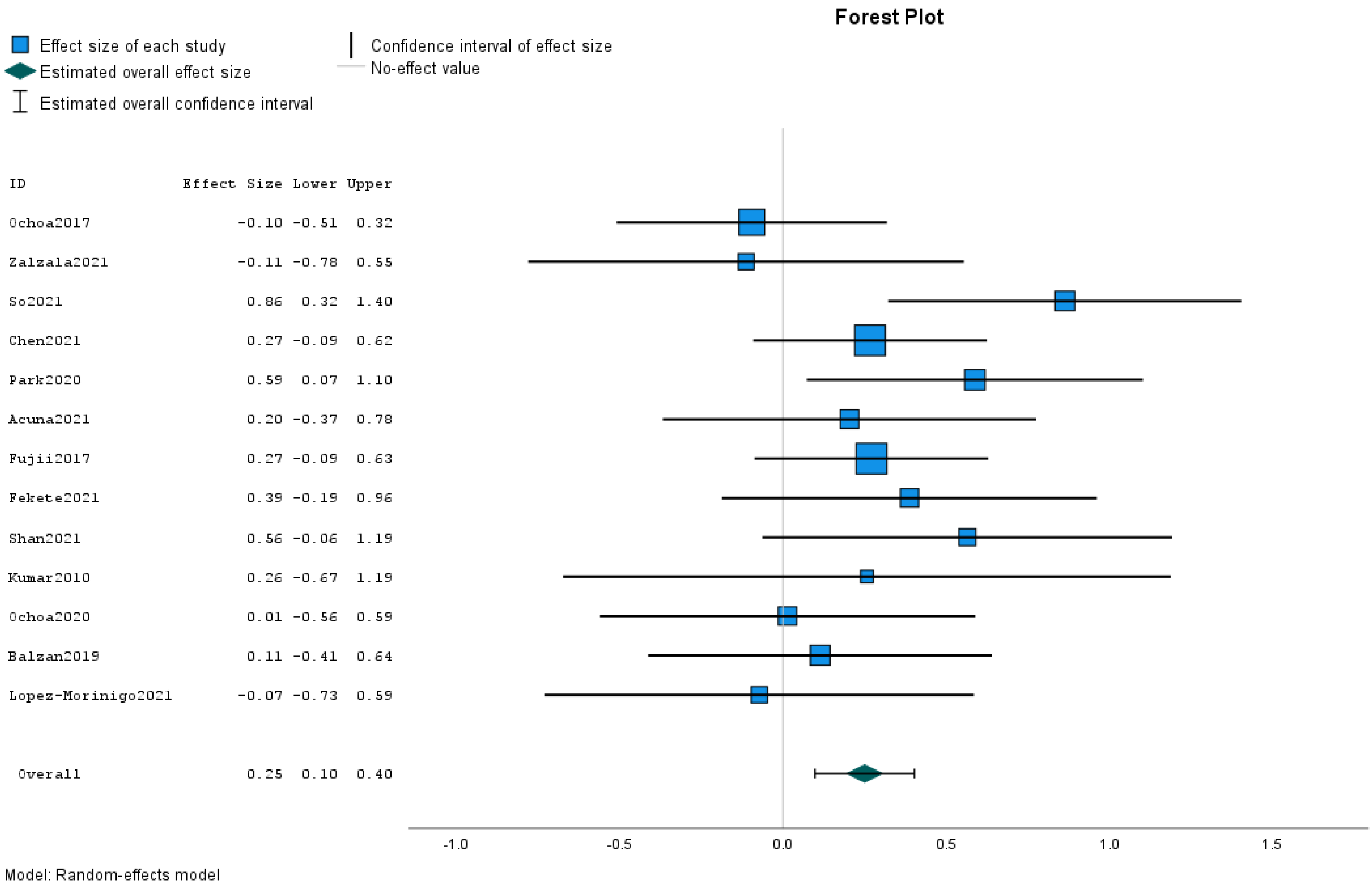

(h) Self-esteem

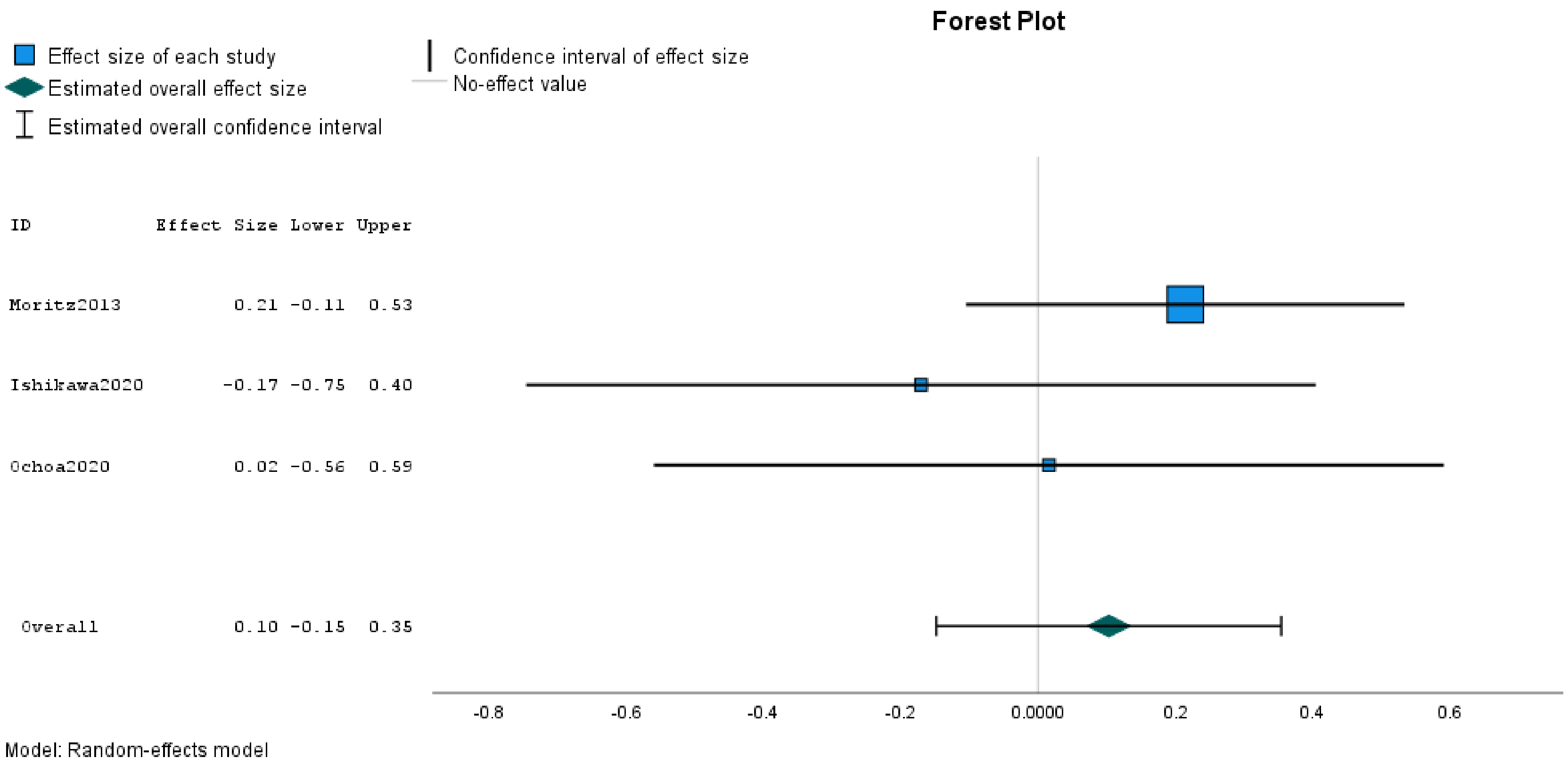

(i) Quality of life

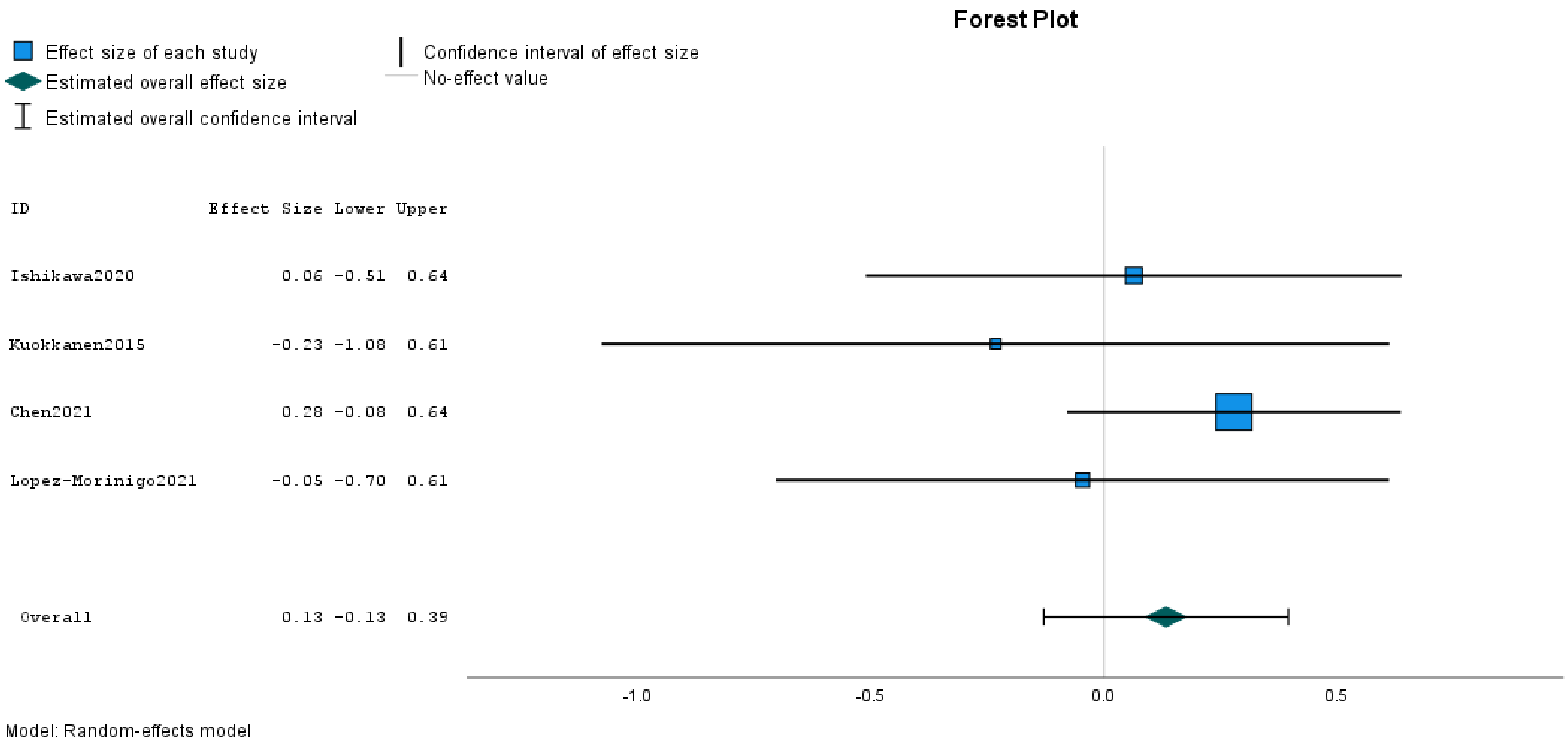

(j) Functioning

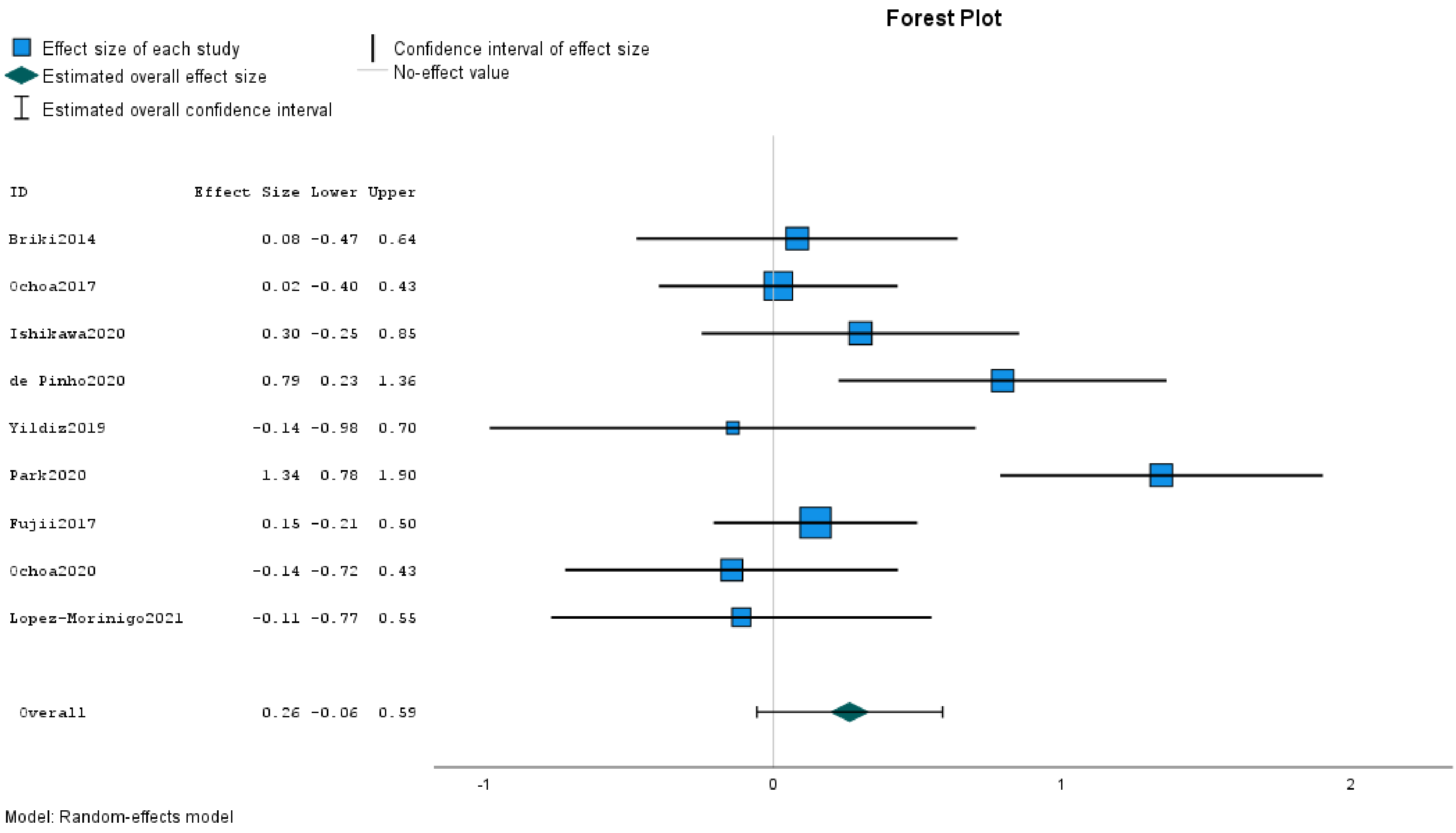

eTable 14. Effect size comparisons between all study designs and RCT-only meta-analyses.

| Outcome | Effect sizes for all study designs | | | | | Effect sizes for RCTs only | | | | |
|---|---|---|---|---|---|---|---|---|---|---|
| | *N reports* | *ES* | *Lower CI* | *Upper CI* | *p-value* | *N reports* | *ES* | *Lower CI* | *Upper CI* | *p-value* |
| **Proximal** | 38 | 0.39 | 0.25 | 0.53 | 0.001 | 26 | 0.37 | 0.20 | 0.53 | 0.001 |
| Positive symptoms | 36 | 0.50 | 0.34 | 0.67 | 0.001 | 24 | 0.47 | 0.25 | 0.69 | 0.001 |
| Delusions | 23 | 0.69 | 0.45 | 0.93 | 0.001 | 15 | 0.64 | 0.32 | 0.96 | 0.001 |
| Hallucinations | 9 | 0.26 | 0.11 | 0.40 | 0.001 | 6 | 0.26 | 0.04 | 0.48 | 0.02 |
| Cognitive biases | 19 | 0.16 | 0.03 | 0.29 | 0.001 | 14 | 0.15 | -0.01 | 0.31 | 0.06 |
| **Distal** | 26 | 0.31 | 0.19 | 0.44 | 0.001 | 19 | 0.25 | 0.11 | 0.39 | 0.001 |
| Self-esteem | 5 | 0.17 | 0.03 | 0.31 | 0.01 | 3 | 0.10 | -0.15 | 0.35 | 0.42 |
| Negative symptoms | 17 | 0.23 | 0.10 | 0.37 | 0.001 | 13 | 0.25 | 0.10 | 0.40 | 0.001 |
| Functioning | 13 | 0.41 | 0.12 | 0.69 | 0.005 | 9 | 0.26 | -0.05 | 0.57 | 0.09 |
| Quality of life | 7 | 0.20 | -0.07 | 0.47 | 0.14 | 4 | 0.13 | -0.13 | 0.39 | 0.32 |

*Note.* Reports Hedges' g effect sizes; pre-post comparisons; RCTs = randomized controlled trials.
